# Noncanonical atherosclerosis as the driving force in tricuspid aortic valve associated aneurysms - A trace collection

**DOI:** 10.1016/j.jlr.2023.100338

**Published:** 2023-02-01

**Authors:** Christian Doppler, Barbara Messner, Teresa Mimler, Bruno Schachner, Marlene Rezk, Clara Ganhör, Christian Wechselberger, Marina Müller, Spela Puh, Johannes Pröll, Barbara Arbeithuber, Thomas Müller, Andreas Zierer, David Bernhard

**Affiliations:** 1 Division of Pathophysiology, Institute of Physiology and Pathophysiology, Medical Faculty, Johannes Kepler University Linz, Linz, Austria; 2 Department of Cardiac Surgery, Cardiac Surgery Research Laboratory, Medical University of Vienna, Vienna, Austria; 3 Department of Cardiothoracic and Vascular Surgery, Kepler University Hospital, Medical Faculty, Johannes Kepler University Linz, Linz, Austria; 4 Experimental Gynecology, Obstetrics and Gynecological Endocrinology, Kepler University Hospital Linz, Johannes Kepler University Linz, Linz, Austria; 5 Center for Medical Research, Medical Faculty, Johannes Kepler University Linz, Linz, Austria; 6 Institute of Organic Chemistry, Faculty of Natural Sciences, Leopold-Franzens University Innsbruck, Innsbruck, Austria

**Keywords:** lipidomics, lysophosphatidylcholine, cholesterol, smooth muscle cells, matrix-assisted laser desorption ionization mass spectrometry, AC, acylcarnitins, BAV, bicuspid aortic valve, HMGCR, 3-hydroxy-3-methylglutaryl coenzyme A reductase, LOQ, Limit of Quantification, LPC, lysophosphatidylcholine, MALDI, matrix-assisted laser desorption ionization, PC, phosphatidylcholines, SMC, smooth muscle cell, TAA, thoracic aortic aneurysm, TAV, tricuspid aortic valve

## Abstract

Pathogenic mechanisms in degenerative thoracic aortic aneurysms (TAA) are still unclear. There is an ongoing debate about whether TAAs are caused by uniform or distinct processes, which would obviously have a major impact on future treatment strategies. Clearly, the ultimate outcome of TAA subgroups associated with a tricuspid aortic valve (TAV) or a bicuspid aortic valve (BAV) is the same, namely a TAA. Based on results from our own and others' studies, we decided to compare the different TAAs (TAV and BAV) and controls using a broad array of analyses, i.e., metabolomic analyses, gene expression profiling, protein expression analyses, histological characterization, and matrix-assisted laser desorption ionization imaging. Central findings of the present study are the presence of noncanonical atherosclerosis, pathological accumulation of macrophages, and disturbances of lipid metabolism in the aortic media. Moreover, we have also found that lipid metabolism is impaired systemically. Importantly, all of the above-described phenotypes are characteristic for TAV-TAA only, and not for BAV-TAA. In summary, our results suggest different modes of pathogenesis in TAV- and BAV-associated aneurysms. Intimal atherosclerotic changes play a more central role in TAV-TAA formation than previously thought, particularly as the observed alterations do not follow classical patterns. Atherosclerotic alterations are not limited to the intima but also affect and alter the TAV-TAA media. Further studies are needed to i) clarify patho-relevant intima-media interconnections, ii) define the origin of the systemic alteration of lipid metabolism, and iii) to define valid biomarkers for early diagnosis, disease progression, and successful treatments in TAV-TAAs.

Thoracic aortic aneurysms (TAA) pose a serious health threat to the general population with an incidence of 5–10 cases per 100,000 persons per year (1). During an asymptomatic progression that can last decades, pathogenesis includes a gradual increase in aortic diameter and consequent weakening of the aortic wall. Due to its silent and hidden progression, TAAs are usually discovered only by chance or at an advanced stage of the disease when specific symptoms occur. Untreated, the weakened aortic wall can ultimately rupture which represents an immediate life-threatening condition with a 30 days lethality of up to 35% (2). To date, TAA pathogenesis is still rather poorly understood and there is no disease-specific pharmacological treatment available. Because TAAs are often detected late, surgical interventions, such as aortic replacement, are often the only remaining treatment options. Although there is some evidence for a role of genetics in initiation and progression, the true reasons for TAA development remain largely unclear (3, 4).

Based on disease etiology, TAAs are generally divided into syndromic, familial nonsyndromic, and degenerative sporadic forms. Because of the heterogeneity of form and origin of these diseases, mechanistic studies to uncover the pathophysiology are complex. All these forms have the development of an aneurysm in common but the underlying mechanisms are multifactorial and as yet only partially understood, if at all. (5). Classic syndromic forms include Marfan, Loeys-Dietz, and Ehlers-Danlos syndrome, whereas familial forms are associated with a wide range of genetic variants. TAAs associated with a congenital bicuspid aortic valve (BAV, only two leaflets are present instead of the three leaflets found physiologically) can occur in, e.g., Marfan patients as well as in the general population (incidence up to 2% in the general population) (6). Among persons bearing a BAV, 35%–80% develop a TAA in the course of their lifespan. Little is known about the pathophysiology of BAV-associated aneurysm, although some mutations or hemodynamic factors have been discussed in recent years (7, 8). Apart from the lack of clarity regarding the pathophysiology of BAV-associated aneurysms, the underlying signaling pathways of other sporadic forms associated with a normal tricuspid valve (with three leaflets) have also not been resolved in detail.

Despite a similar pathological appearance, the enlargement of the aortic diameter in tricuspid aortic valve (TAV)-TAAs and BAV-TAAs, there is more and more evidence arising that the basis of disease pathogenesis may differ fundamentally. For example, while the aortic diameter in BAV-TAAs increases much faster and starts significantly earlier in life, histological wall structures cannot be discriminated from healthy aortas. In contrast, TAV-TAAs exhibit massive alterations in aortic wall architecture, e.g., a thickening of the tunica intima, a thinning of the tunica media, and the display of significant signs of atherosclerosis. Further, on the cellular level, an accumulation of DNA damage and a loss of cell contractility is observed in TAV-TAAs only (9, 10, 11). Although not previously linked, Stern *et al.* showed that atherosclerotic changes are more frequent in TAV-TAAs than previously thought (11). No such association has yet been established for BAV-TAAs. In addition, it is already known that atherosclerotic changes in the intima have far-reaching consequences for the entire vessel. As early as 1933, van der Wal *et al.* reported that atherosclerosis in coronary arteries and the descending aorta leads to a decrease in medial thickness with simultaneous immune-infiltration and formation of vessels (12). The data on this was summarized by Milutinović *et al.* in 2020 (13). Aside from immune infiltrations, deposition of lipids is central for atherosclerosis development. Already in the year 1980 it was hypothesized that increased lipid amounts in the media originate from the atherosclerotic lesions of the intima (14).

During recent years, metabolomic analyses have become a very useful tool in medical research. It enables the understanding of disease-inducing and disease-induced changes in metabolism and allows for the development of highly sensitive risk assessment and disease progression markers. Particularly also in cardiovascular diseases, e.g., during late-stage heart failure, oxidized cholesterol was found to accumulate in blood plasma. In addition, increased levels of ceramides in blood plasma have been demonstrated to be associated with an increased risk for cardiovascular diseases in general. Plasma concentrations of various phosphatidylcholines (PC) and lysophosphatidylcholines (LPC) are also found to positively correlate with the risk for cardiovascular diseases (15, 16).

In a previous study we demonstrated alterations in the lipid composition in the aortic tissue from TAA patients, with increased concentrations of sphingomyelins compared to non-aneurysmal tissue (17). Together with the results from Stern *et al.* regarding pathological changes in the intima and media of TAA patients, our previous findings prompted us to perform a more holistic approach to better characterize and describe the role of lipids, lipid metabolism relevant proteins and genes in the course of TAA development. Importantly, the data presented in this work not only cover tissues but also examine in detail isolated primary aortic smooth muscle cells (SMCs) and patient blood plasma and use a variety of technologies, including targeted metabolomics and matrix-assisted laser desorption ionization (MALDI) imaging.

## MATERIALS AND METHODS

### Study design and experimental setup

The design of this study was approved by the Ethics Committees of the Medical University of Innsbruck (EK1 09.11.12), the Medical University of Vienna (EK Nr: 1183/2012 and EK Nr: 1840/2021), and the Johannes Kepler University Linz (EK Nr: 1111/2018). All patients included in this study gave their written informed consent. These studies conform to the Declaration of Helsinki. Following matching for age and sex, sample types (as described below) from ascending TAA patients and organ donors were subjected to gene expression analysis, metabolomic profiling, MALDI imaging as well as histological and immunofluorescence-based stainings. The aneurysm samples originated from patients undergoing surgery for thoracic aorta replacement. Control samples were obtained from organ donors showing no evidence of aneurysm or valvular disease, as well as from cardiology patients undergoing routine examination, if an aneurysm could be excluded by echocardiography. For a detailed description of the analyzed samples, we would like to refer to Table 1. Regarding the clinical characteristics, no significant differences in age, sex, and preexisting conditions were found between the three experimental groups. Patients suffering from inflammatory aortic disease or systemic connective tissue disorders (e.g., Marfan syndrome or Ehlers Danlos syndrome) were excluded from the study. A detailed list on patient characteristics is given in Table 2.

**Table 1:** Analyses and number of samples

	Controls	BAV-TAA	TAV-TAA
Plasma analyses	26	27	27
Isolated primary smooth muscle cell lines	8	10	14
MALDI analyses	8	5	7
Histological/immunofluorescence based staining	17	17	17

BAV, bicuspid aortic valve; TAA, thoracic aortic aneurysm; TAV, tricuspid aortic valve.

**Table 2:** Clinical information on control and aneurysm patients

				*P*		
	C	BAV	TAV	C versus BAV	C versus TAV	BAV versus TAV
Number	n = 33	n = 34	n = 34	ns	ns	ns
Age (years)	61.7 ± 9.0	61.0 ± 9.7	62.9 ± 6.1	ns	ns	ns
Gender (male)	57.6%	61.8%	58.8%	ns	ns	ns
Aortic diameter (mm)	nd	49.9 ± 5.2	52.5 ± 7.2	nd	nd	ns
Current smoker	18.2%	14.7%	26.5%	ns	ns	ns
Coronary heart disease	12.1%	17.6%	17.6%	ns	ns	ns
Hypertension	45.5%	44.1%	61.8%	ns	ns	ns
Hyperlipidemia	30.3%	52.9%	50.0%	ns	ns	ns
Diabetes	18.2%	5.9%	17.6%	ns	ns	ns
Aortic stenosis	nd	44.1%	23.5%	nd	nd	ns
Aortic regurgitation	nd	47.1%	52.9%	nd	nd	ns
Total cholesterol	nd	188.0 ± 38.2	186.7 ± 24.1	nd	nd	ns
HDL	nd	68.9 ± 15.4	63.8 ± 23.4	nd	nd	ns
LDL	nd	101.7 ± 19.3	108.4 ± 22.3	nd	nd	ns
Triglycerides	nd	136.9 ± 24.3	131.4 ± 25.6	nd	nd	ns

Data are presented as mean and standard deviation or percent.

BAV, bicuspid aortic valve; C. controls; nd, not determined; ns, not significant; TAA, thoracic aortic aneurysm; TAV, tricuspid aortic valve.

### Histological and immunofluorescence-based staining on tissue sections

Fixation and dehydration of aortic tissue samples and subsequent histological and immunofluorescence-based staining was performed as described elsewhere (11). The Elastica van Gieson staining (Sigma, #HT-25) allows detecting and visualizing the layers of the aorta, media degeneration, as well as atherosclerotic changes. Histological staining on 3 μm tissue sections was performed according to the manufacturer's protocol.

For the classification of atherosclerotic changes in the aortic tissue, an adapted American Heart Association classification was used, as described elsewhere (11). In brief, aortic tissue samples were classified into six grades of atherosclerotic changes: grade 0 having no intimal thickening; grade I with obvious visible thickening of the intimal layer; grade II showing intimal thickening with isolated foam cells; grade III with more than 20 cell layers of the intima including foam cells and leukocyte infiltrates; grade IV displaying an enhanced thickening of the intima with foam cell infiltration and accumulation of cholesterol crystals; and grade V showing an severe atherosclerotic plaque. Additionally, Elastica van Gieson stained tissue sections were used to assess the degree of medial degeneration of samples as described by Stern *et al.* and Roberts *et al.* (11, 18). In detail, the degree of degeneration was divided into five grades: no media degeneration (completely intact elastic fibers), minimal media degeneration (isolated areas showing fiber breaks and fiber losses), intermediate media degeneration (certain areas with complete degeneration of the elastic fibers), advanced media degeneration (loss of elastic fibers at several sites in the media), and severe media degeneration (complete loss of elastic fibers in large areas of the media).

For the immunofluorescence-based staining, 5 μm aortic tissue sections were used. Prior to staining, antigen retrieval and subsequent permeabilization using 0.2% TritonX-100 was performed. Nonspecific binding sites were blocked by incubating the tissue sections with 10% goat serum and 1% BSA in TBS. Sections were incubated with different primary antibodies (diluted in 1% BSA/TBS) at 4°C overnight. The following antibodies were used: Mouse anti-CD68 antibody [1:150, Thermo Fisher Scientific, Austria, #MA5-13324, to detect macrophages], rabbit anti-3-hydroxy-3-methylglutaryl coenzyme A reductase (HMGCR) antibody [1:100, Novus biologicals,United States, #NBP1-91996], mouse anti-MCP-1 antibody [monocyte chemoattractant protein 1; 1:500, Thermo Fisher Scientific, Austria, #MA5-17040], rabbit anti-LDL-R antibody [1:400, Proteintech, United States, #10785-1-AP], rabbit anti-SREBF1 antibody [1:100, Proteintech, #14088-1-AP]. In addition to the above epitopes, SMC actin was also stained in the sections to visualize SMCs (with rabbit anti-alpha SMC actin antibody [1:2,500, Proteintech, #14395-1-AP] or mouse anti-alpha SMC actin antibody [1:50, Dako, #M0851]). Suitable fluorescent labelled goat anti-rabbit or anti-mouse antibodies were used as secondary antibodies (1:800, Alexa Fluor 546 or Alexa Fluor 488-conjugated, Invitrogen) which were incubated for 60 min at room temperature. The nuclei were stained with Hoechst 34,580 (1:10,000, MedChemExpress, #HY-15560B), and sections were subsequently mounted using ProLong Gold antifade reagent (Invitrogen, #P36930). Images were acquired using a Nikon Ti Eclipse confocal microscope at magnifications of 40× and 60× at random spots in the intimal and medial layer of the sample. Representative images of Elastica van Gieson stained tissue sections for determination of atherosclerotic changes and media degeneration were taken at a magnification of 20× (large image of two pictures for atherosclerosis). Large images of Elastica van Gieson stained sections as a comparison for the MALDI images were taken at a magnification of 10× or 40×. Image analysis and quantification of the positive signals was performed using NIS elements AR software (https://www.microscope.healthcare.nikon.com/de_EU/products/software/nis-elements).

### MALDI imaging

Frozen tissue samples were cut into 8 μm thick sections using a cryotome (Leica Biosystems, Wetzlar, Germany), followed by thaw mounting onto indium-tin-oxide coated glass slides (Bruker Daltonics, Bremen, Germany). Slides were dried at room temperature under nitrogen atmosphere in a desiccator, followed by application of the MALDI matrix using an automatic vibration vaporization system (ImagePrep, Bruker Daltonics, Germany). 2,5-Dihydroxybenzoic acid (50 mg/ml in Acetonitrile:H_2_O 1:1) was sprayed at following system settings: 60 cycles; 20% spray power; 30% spray modulation; 2 s spray time; 30 s incubation time; and 60 s dry time (N_2_ flow provided at 2×10^5^ Pa). MALDI mass spectrometry imaging was performed using a MALDI-TOF/TOF mass spectrometer (Autoflex speed, Bruker Daltonics, German) equipped with a 1 kHz Nd:YAG smartbeam laser. All analyses were performed in positive mode after an external calibration with a peptide calibration standard (Bruker Daltonics, Germany) containing angiotensin II, angiotensin I, substance P, bombesin, ACTH clip 1–17, ACTH clip 18–39, and somatostatin 28. MS spectra were acquired in the mass range of m/z (mass to charge ratio) 200–2000 Da in reflector mode with 200 laser shots at 35% power with no random walking. In all experiments, the laser diameter value was set to “minimum.” Mass spectrometry imaging images were generated with FlexImaging 3.0 software package (Bruker Daltonics, Germany, https://bruker-daltonics-fleximaging.software.informer.com/) and SCiLS Lab software (SCILS GmbH, Germany, https://www.bruker.com/en/products-and-solutions/mass-spectrometry/ms-software/scils-lab.html), where data was normalized using Total Ion Count. Monoisotopic masses of protonated lipids ([M+H]^+^) and sodium adducts ([M+Na]^+^) of metabolites were used for PC and LPC identification, where distribution of protonated and sodium adducts needed to be equal with higher intensities of sodium adducts in order for a compound to be considered identified. Mass spectrums were checked if the isotopologue pattern fit the described lipid (i.e., peaks only stemming from isotopologue of different monoisotopic masses were excluded) and if only one m/z peak was present in the respective range of m/z ± 0.125%.

### Cell isolation and cultivation

Primary SMCs were isolated from aneurysmal and control tissue of the ascending aorta as described in more detail elsewhere (11, 19). Cells were cultivated in specific SMC medium (Medium 231 supplemented with SMC Growth Supplement; Gibco, Thermo Fisher Scientific, Austria) at 37°C until reaching passage 3 before being used for experiments. All SMC samples were tested for contamination with endothelial cells and fibroblasts by Western blot analyses using an anti-CD90 antibody for fibroblasts (Dianova, Germany, #DIA-100) and an anti-CD31 antibody for endothelial cells (Dako, United States, #M082301).

For metabolomic analyses, trypsinized cells were counted and 1x10^6^ cells per sample were added to 100 μl ice cold ultrapure absolute Ethanol (Carl Roth, Karlsruhe, Germany) for cell lysis. Samples were sonicated (15 intervals, cycles: 10%, power: MS72/; Sonopuls HD 200; Bandelin, Berlin, Germany) and all solid components were separated by centrifugation at 10,800 *g* at 4°C for 10 min. Supernatants were collected and stored at −80°C for further analyses.

### Gene expression analyses of isolated primary SMCs

Total RNA was extracted from isolated aortic cells (n=3 for controls, BAV-TAA, and TAV-TAA) using TRIzol reagent (Life Technologies, Carlsbad, CA) and purified with the RNeasy mini kit (Qiagen, Valencia, CA) according to the manufacturers’ instructions. The quality and quantity of RNA samples were determined with a UV–Visible spectrophotometer (Thermo Fisher Scientific, NanoDrop 2000) at 260 nm/280 nm absorbance. The long non-coding ribonucleic acid and messenger ribonucleic acid transcription profiles of the samples were determined by Clariom D solutions for the Affymetrix Gene Chip (Santa Clara, CA). The microarray analysis was performed using Transcriptome Console Software (Thermo Fisher Scientific, Version 1.2.1, https://www.thermofisher.com/at/en/home/life-science/microarray-analysis/microarray-analysis-instruments-software-services/microarray-analysis-software/affymetrix-transcriptome-analysis-console-software.html). Primary data (CEL files) were normalized at the transcript level using the robust multiarray average method. The median summarization of transcript expressions was calculated according to the instructions. Signal scanning was performed using Affymetrix equipment (Affymetrix Gene Chip Operating Software, https://www.affymetrix.com/support/technical/datasheets/gcos_datasheet.pdf). Genes involved in lipid metabolism that showed at least a 2-fold change in their expression between the two groups were defined as deregulated. According to the publication of Jung K. *et al.*, we decided to consider a 2-fold change in gene expression as relevant and used this parameter instead of a *P*-value (20).

### Plasma sampling

Blood was drawn using K3EDTA tubes (Greiner Bio One, Austria) and stored at room temperature for no longer than 30 min. Plasma was obtained via centrifugation at 2,500 *g* at room temperature for 10 min. Aliquots were stored at −80 °C until metabolomic analysis.

### Metabolomic analyses of isolated primary SMCs and plasma samples

Plasma and cell extracts were analyzed using the MxP Quant 500 and AbsoluteIDQ1 p150 kit (BIOCRATES Life Sciences AG, Innsbruck, Austria), respectively. Metabolite quantification was done according to the manufacturer’s protocol; a detailed description can be found in the Online Supplement. In brief, 10 μl of samples were dried, amino acids, and biogenic amines were derivatized and metabolites were extracted. Flow injection analysis using tandem mass spectrometry was utilized for the detection of acylcarnitines (AC), lipids, and hexose, ultra performance liquid chromatography-MS/MS was used for all other metabolite classes. Measurements were carried out on a ultra performance liquid chromatography I-class PLUS (Waters, United States) system coupled to a SCIEX QTRAP 6500+ mass spectrometer (AB Sciex Deutschland GmbH, Darmstadt, Germany). Cell culture samples were analyzed as described previously (17). Data were generated using the Analyst software suite (Sciex, https://sciex.com/products/software/analyst-software) and transferred to the MetIDQ software (Biocrates, https://biocrates.com/webidq/) for further data processing and analyses, where metabolites were identified and quantified. For further details, see the Online Supplement.

### Statistical analyses

Statistical analyses were performed using IBM SPSS Statistics (Version 26) and Graph Pad Prism (Version 8) software (https://www.graphpad.com/scientific-software/prism/). A Chi square test was used to check for differences in atherosclerosis grade and grade of media degeneration between the three sample groups. For the correlation of atherosclerosis with media degeneration among each group, a Spearman correlation test was performed. Differences in protein expression levels between controls, the BAV group, and the TAV group were analyzed using a Kruskal-Wallis test (after Gaussian distribution revealed nonparametric distribution). If a metabolite was below the LOQ (Limit of Quantification) in more than 50% of the samples from one group, it was defined as not quantifiable. As soon as this was the case for the corresponding metabolite in all three groups investigated, the metabolite was excluded. However, if the metabolite in one of the groups reached a value above the LOQ in more than 50% of the samples, the values of all three groups were included, as it could be a significant difference between the groups. Concentrations below LOQ were replaced by the respective limit of detection value. Outliers were detected and removed in GraphPad Prism (Version 7.03) using ROUT method (Q=1%). Data were normalized using z-scores in order to rule out overestimation of highly abundant or underestimation of less abundant metabolites. The sums of normalized concentrations for different metabolite classes were calculated. After detecting normal distribution of data, one-way ANOVA with Bonferroni correction was performed for normally distributed samples to detect significantly upregulated or downregulated metabolites. A *P*-value of <0.05 was seen as significant difference. Box plots with whiskers for graphical illustration were generated using GraphPad Prism.

## RESULTS

### Clear signs of intimal proatherosclerotic alterations and media degeneration in TAV-TAAs but not in BAV-TAAs

**Fig. 1:**
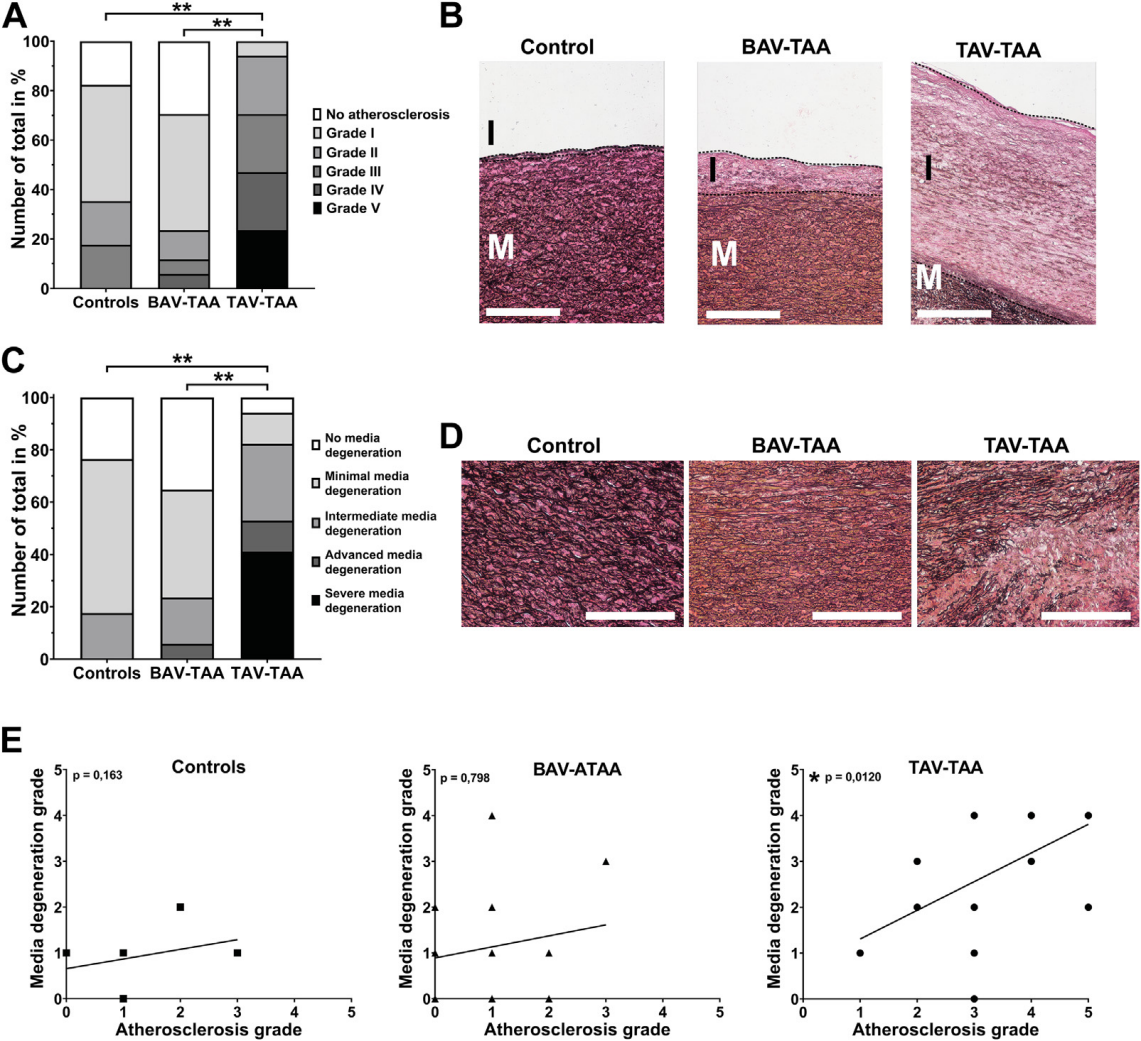
Atherosclerosis and a degenerated media are typical sign for TAV-TAAs but not for BAV-TAAs. A: Quantification of atherosclerotic changes in controls, BAV-TAAs, and TAV-TAAs, evaluated using an adopted American Heart Association grading scheme. Data are shown as number of total in percent. In (B), representative images of Elastica van Gieson stained tissue sections used for determination of atherosclerotic changes are shown (large image, images taken at 20x magnification; I = intima; M = media; black dotted lines show the borders of intima/intimal hyperplasia). In (C) the quantification of media degeneration using the described grading system is shown for aneurysmal as well as for control tissue. Data are shown as number of total in percent. In (D) representative images of Elastica van Gieson stained tissue sections used for the determination of media degeneration are shown. Red/pink color indicates collagen, black color stains elastic fibers, and light-yellow color indicates muscle (images were taken at a magnification of 20×; I = intima; M = media.). Scale bar indicates 250 μm. E: Correlation analyses of atherosclerosis with media degeneration of each sample group are shown. Number of samples analyzed per group are control = 17, BAV-TAA = 17, TAV-TAA = 17. ∗...indicates a *P*-value < 0.05; ∗∗…indicates a *P*-value < 0.01. TAV, tricuspid aortic valve; BAV, bicuspid aortic valve; TAA, thoracic aortic aneurysm.

While there are no reports at all available describing atherosclerotic processes to occur in BAV-TAA pathogenesis, a few studies provide such data for TAV-TAA pathogenesis. In the present study, we can confirm these, still rare, reports. As shown in Fig. 1A, the aneurysmal aorta of the TAV-TAAs has significantly more severe atherosclerotic changes than the control group and the BAV-TAA group (representative images in Fig. 1B). With regard to atherosclerotic changes in the media, this evaluation additionally shows how similar the BAV-TAA group and the control group are (see supplemental Table S1). Apart from atherosclerosis, other processes occur in the aortic media being central for aneurysms development in both, the TAV-TAAs and the BAV-TAAs. It has been proposed for decades that an aneurysm is characterized by degeneration of the aortic media. Previous data and our own data demonstrate that this is the case for TAV-TAAs but not for BAV-TAAs (Fig. 1C with representative images in 1D). In order to test for potential interrelations between media degeneration and atherosclerosis correlation analyses were performed. These analyses indicate that media degeneration and the degree of atherosclerosis correlate significantly in the TAV-TAA group, a result being indicative of such interrelations. No such correlations could be found in the control group and in the BAV-TAA group (Fig. 1E). Detailed information on the degree of atherosclerosis and media degeneration of each sample is provided in the supplemental Table S1.

### Accumulation of macrophages in the media is characteristic for TAV-TAAs but not for BAV-TAAs

**Fig. 2:**
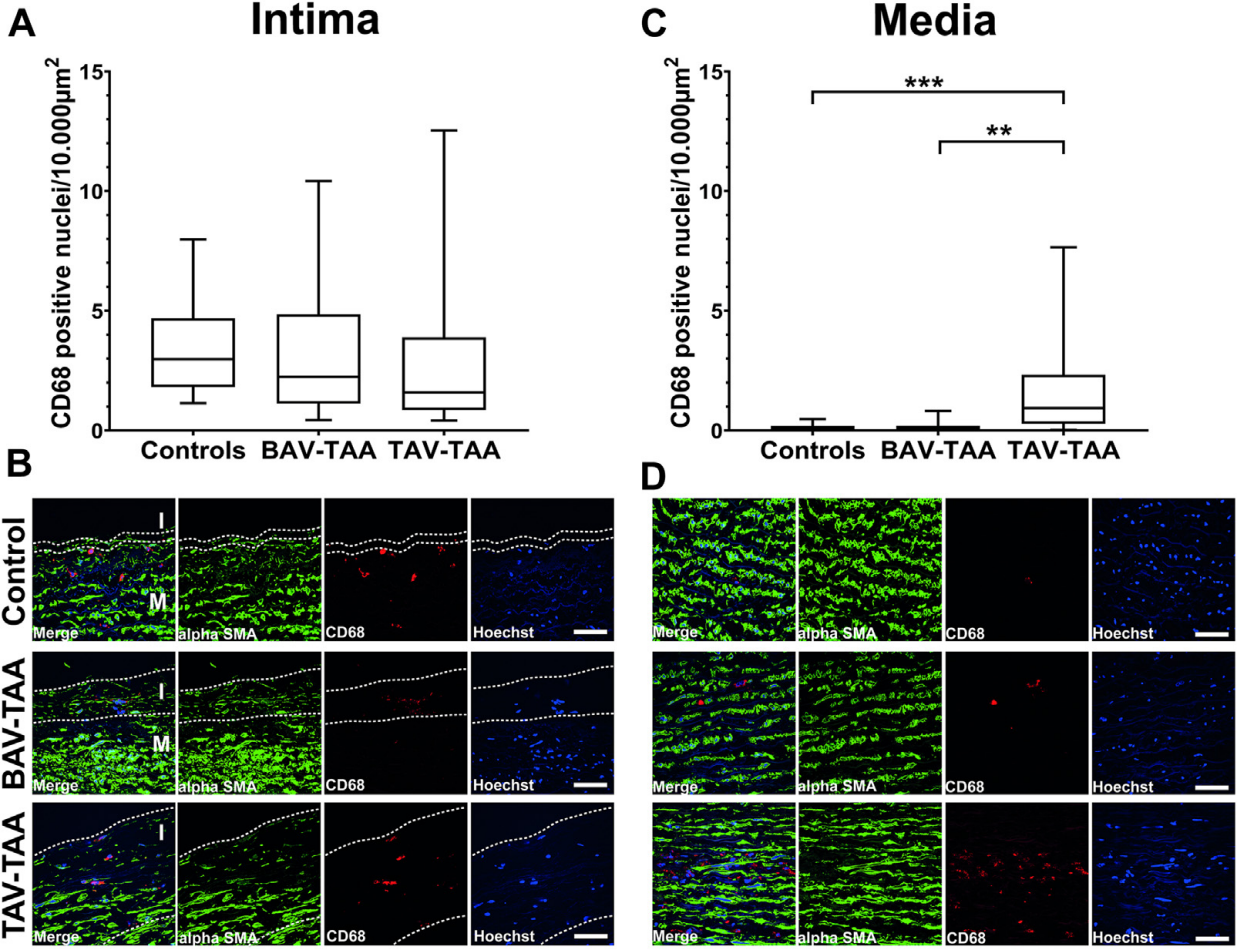
Massive accumulation of macrophages in the media occurs exclusively in TAV-TAAs. A: Quantification of the number of CD68 positive nuclei per 10.000 μm^2^ in the intimal layer of controls, BAV-TAAs, and TAV-TAAs. B: Depicts representative images of the intima CD68 staining from (A); green color indicates alpha smooth muscle actin, red indicates CD68 positive cells, and blue color indicates cell nuclei stained using Hoechst (white dotted lines are indicating the border of the intimal layer). C: Quantification of the number of CD68 positive nuclei per 10.000 μm^2^ in the medial layer of controls, BAV-TAA, and TAV-TAA. D: Shows representative images of the medial CD68 staining from (C); green color indicates alpha smooth muscle actin, red indicates CD68 positive cells, and blue color indicates cell nuclei stained using Hoechst. White line within the representative images indicates a scale bar of 50 μm. Data are shown as box plots with whiskers. Alpha SMA = alpha smooth muscle actin; I = intimal layer; M = medial layer. Number of samples are control = 17, BAV-TAA = 17, TAV-TAA = 16. ∗∗…indicates a *P*-value < 0.01; ∗∗∗…indicates a *P*-value < 0.001. TAV, tricuspid aortic valve; BAV, bicuspid aortic valve; TAA, thoracic aortic aneurysm.

Atherosclerosis is characterized by infiltrating macrophages (CD68 positive cells) and an inflammatory response, which significantly affects the atherosclerotic disease status. As the disease progresses, further macrophages infiltrate the intimal atherosclerotic lesion, absorb lipids, transform into foam cells, and remain within the tissue due to their reduced ability to migrate (21). In contrast to the general believe of macrophage accumulation in advancing intimal atherosclerotic disease, our analysis indicates that increased atherosclerosis in TAV-TAAs was not accompanied by an increased number of macrophages within intimal hyperplasia/atherosclerotic lesions (Fig. 2A and representative images in 2B). In contrast, however, our analyses also show that there was a significant accumulation of macrophages in the media of TAV-TAAs when compared to the control group and the BAV-TAAs (Fig. 2C and representative images in 2D). As a scan through the media demonstrates (see supplemental Fig. S1), these macrophages were found throughout the media and are not located to specific areas. Interestingly, increased expression of MCP-1, the major chemoattractant of macrophages, could not be detected neither in the intima nor in the media (see supplemental Fig. S2), which may rather argue for residing macrophages.

### Isolated SMCs from TAV-TAA exhibit significantly increased levels of LPCs and ACs

**Fig. 3:**
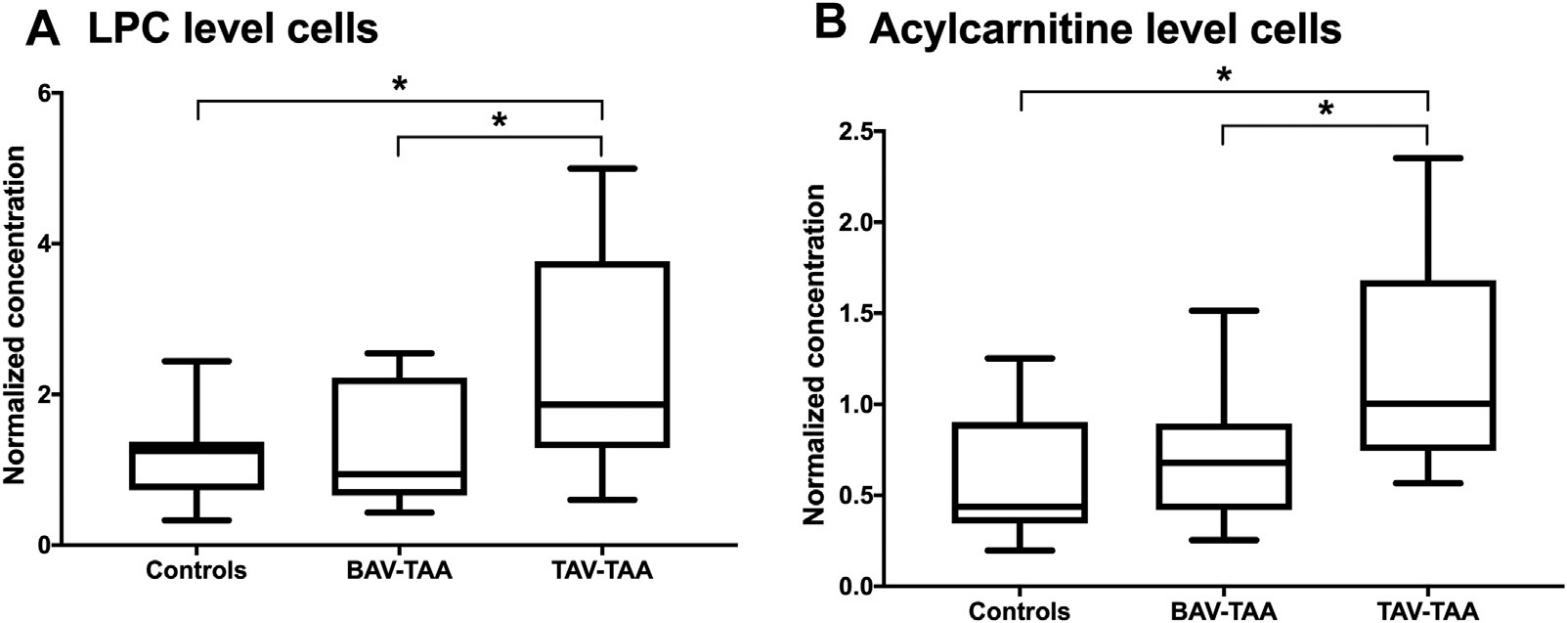
Isolated SMCs from TAV-TAAs exhibit significantly increased levels of lysophosphatidylcholines and acylcarnitines. A: Shows normalized concentrations of LPCs in isolated SMCs from controls, BAV-TAAs, and TAV-TAAs. B: Provides normalized concentration of acylcarnitines in isolated primary SMCs from. Number of cell lines included in these analyses are control = 8, BAV-TAA = 10, TAV-TAA = 14. Data are presented as box blots with median and whiskers. ∗...indicates a *P*-value < 0.05. BAV, bicuspid aortic valve; SMC, smooth muscle cell; TAA, thoracic aortic aneurysm; TAV, tricuspid aortic valve.

Atherosclerotic alterations in the aortic intima and media are characterized by altered lipid profiles and inflammation. Consequently, an in-depth analysis of lipids and lipid profiles of primary isolated aortic SMCs was performed. Tunica media SMCs from controls, TAV-TAAs, and BAV-TAAs were isolated and propagated under identical conditions and then analyzed. When classifying individual lipids into lipid classes, we found significant differences in lipid class concentrations between controls, BAV-TAAs, and TAV-TAAs. In detail, SMCs isolated from TAV-TAA samples showed higher levels of LPCs (*P* = 0.013) and AC (*P* = 0.010) than in controls. Interestingly, the lipid concentrations of BAV-TAA and control SMCs were very similar and showed no differences in lipid class concentrations between the groups. Clearly, also the concentration of LPCs (*P* = 0.034) and ACs (*P* = 0.015) was significantly higher in the TAV-TAA cells than in the BAV-TAA group (see Fig. 3A, B). In order to exclude that very abundant lipids are overestimated or less abundant lipids are underestimated, the data were analyzed in a normalized form. Consequent analyses with nonnormalized data (absolute concentrations) gave the same results. The data for nonnormalized analyses are presented in the online supplement (see supplemental Fig. S3). Principal component analysis allows separation of control and BAV groups from the TAV-TAA group based on the variance described by lipid concentrations (for details see supplemental Fig. S4).

### MALDI imaging provides characteristic lipid profiles for aneurysmal aortas

**Fig. 4:**
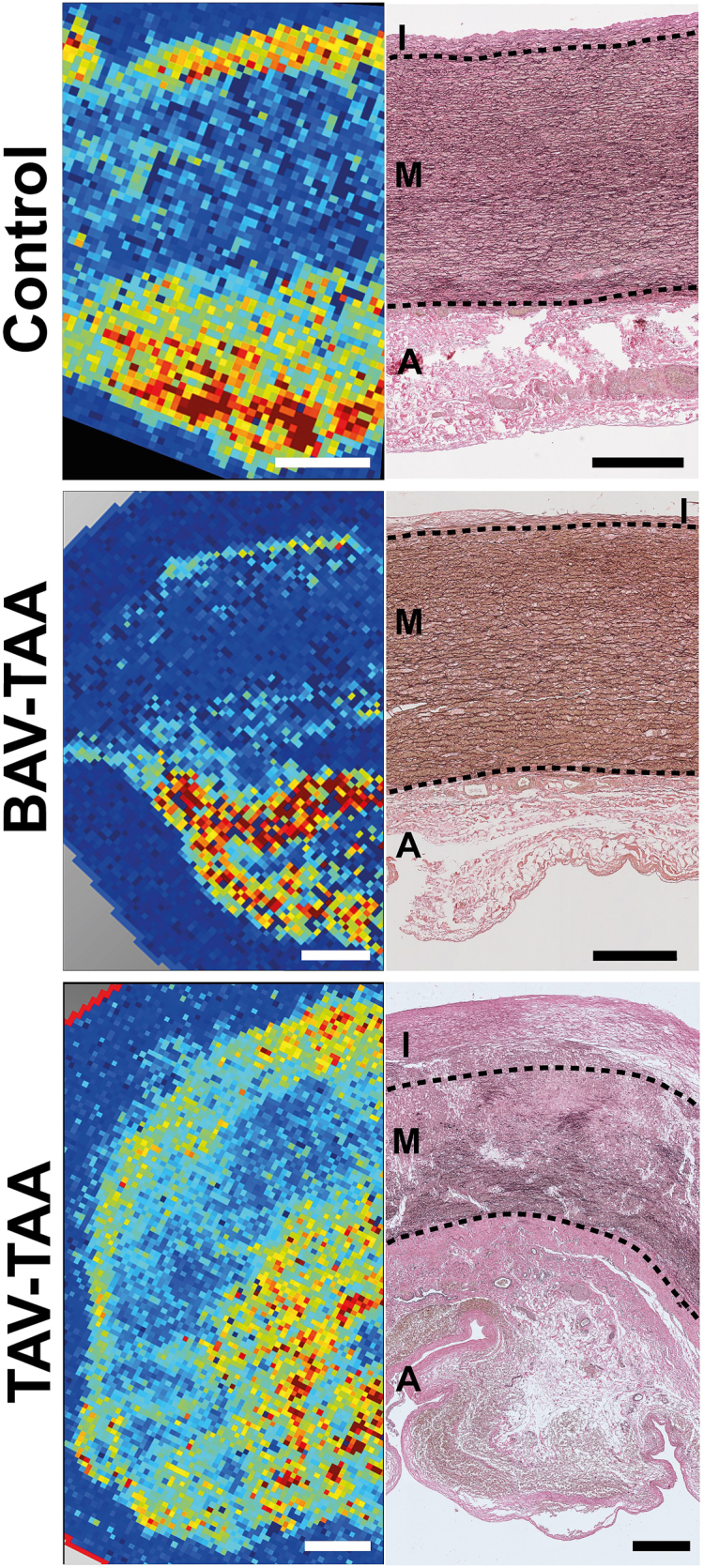
MALDI analyses show specific metabolic patterns that only partly match classical histological structures. The left column of images shows representative MALDI image data of cryo-tissue sections of control aorta, BAV-TAA, and TAV-TAA. Data presented refer to the following molecule/s PC(34:2) (C_42_H_80_NO_8_P), identified as protonated molecule (m/z = 769.562 ± 0.125%) and sodium adduct (m/z = 780.551 ± 0.125%). The concentrations of the lipid are color-coded: red indicating the high concentrations, yellow/green intermediate concentrations, and blue low concentration/not detectable. Scale bar (white) indicates 750 μm. To get an overview of the structure of the aorta, the right column contains the corresponding Elastica van Gieson staining to identify individual layers. Red/pink color indicates collagen, black color indicates elastic fibers, and light-yellow color indicates muscle (I = intima; M = media; A = adventitia). Black dotted lines are indicating the borders of the vessel layers. Scale bar (black) indicates 500 μm. Please note that there is a reuse of images of Figure 4 in supplemental Figs. S1and S6. The rationale for the reuse of images is to allow for a better comparison of different analyses and stainings. BAV, bicuspid aortic valve; MALDI, matrix-assisted laser desorption ionization; TAA, thoracic aortic aneurysm; TAV, tricuspid aortic valve.

Based on the quantitative differences in SMC lipid concentrations and profiles between the groups we decided for a MALDI imaging approach to generate more qualitative data. Based on the analyses of cells the imaging approach was also focused on PCs and LPCs, as these lipid species showed up the highest number of detectable metabolites which are often complexed with cholesterol (22). In detail, PCs and LPCs were identified using their monoisotopic masses for m/z ratios of sodium adducts ([M+Na]^+^) or protonated lipids ([M+H]^+^). Sodium adducts showed higher signal intensities than in protonated PCs or LPCs across all investigated species of these lipid classes. Distribution pattern of these two adducts had to be identical in order for a lipid to be considered identified. MALDI imaging revealed a robust and similar lipid signal within the metabolically highly active adventitial layer in all three groups (see Fig. 4). Further lipid accumulations were observed in the intimal layer in all three groups, roughly yielding similar amounts of lipids. Regarding lipid distribution in the tunica media, BAV-TAAs showed the lowest PC concentration, followed by the control group, which showed an only slightly higher concentration. In contrast, a considerable accumulation of PCs was observed in the medial layer of TAV-TAAs compared to the other two groups. This result is consistent with the significantly increased lipid levels in SMCs of the media of TAV-TAAs (Fig. 3). Unfortunately, we could not identify a single PC or LPC that was increased only in the media. Based on limitations in spatial resolution of MALDI imaging, the cellular monolayer of the tunica intima could not be analyzed and depicted properly. More MALDI image analyses are given in the online supplement (see supplemental Fig. S5). These images are showing high lipid concentrations within the intimal hyperplasia and adventitial layer of all three groups. Moreover, these images are showing clearly an increased concentration in the medial layer of TAV-TAA samples compared to the BAV-TAA group.

### Gene expression profiling confirms perturbation in TAV-TAA lipid metabolism

Based on the results from lipid analysis of isolated cells and tissue analyses by MALDI imaging, we focused on the analysis of SMC gene expression. For this approach the same cells that were previously used for lipid analyses were also used for gene expression studies. All genes with an at least 2-fold difference in expression between groups were classified as differentially expressed. As shown in Table 3, 20 genes show trends of being differentially expressed between the TAV-TAA and the control group. Interestingly, all of these genes were overexpressed in TAV-TAAs compared to the control. Based on these results, seven specific pathways have been identified that may play a central role in causing the different, and potentially disease relevant, phenotypes between the groups. Our analyses showed that in the TAV-TAA group, particularly the cholesterol and lipid metabolism differed significantly from healthy control SMCs and the BAV-TAA SMCs (see supplemental Table S2). Seven genes central to cholesterol metabolism and six genes central to lipid metabolism were found to be overexpressed in TAV-TAA SMCs compared to control cells. Of the genes found to be more than 2-fold dysregulated, *LDLR* (4.1-fold overexpression, *P*-value = 0.04) and *HMGCR* (2.1-fold overexpression, *P*-value = 0.88) have been described to play a central role in lipid and cholesterol metabolism. For an overview, all genes identified as dysregulated more than 2-fold and those with a *P*-value < 0.05 are listed in the online supplement (see supplemental Table S3). In summary, the highest number of differentially expressed genes were found between TAV-TAA and BAV-TAA, and the lowest number of differentially expressed genes were observed between control group and BAV-TAA.

**Table 3:** List of deregulated metabolic pathways and number of deregulated genes in TAAs

Metabolic Pathway	C versus TAV-TAA	C versus BAV-TAA	BAV-TAA versus TAV-TAA
Cholesterol metabolism	6	1	3
Lipid metabolism	5	1	2
Glycerophospholipid metabolism	1	−	−
Synthesis of PC	1	−	−
Acyl chain remodelling of PC	3	1	1
Carnitine metabolism	1	−	−
Alpha-linolenic and linoleic acid metabolism	3	2	2
Total number of deregulated gene	n|N = 20	n|N = 5	n|N = 8

BAV, bicuspid aortic valve; C. controls; PC, phosphatidylcholine; TAA, thoracic aortic aneurysm; TAV, tricuspid aortic valve.

### HMGCR protein expression is significantly increased in the intima of TAV-TAAs

**Fig. 5:**
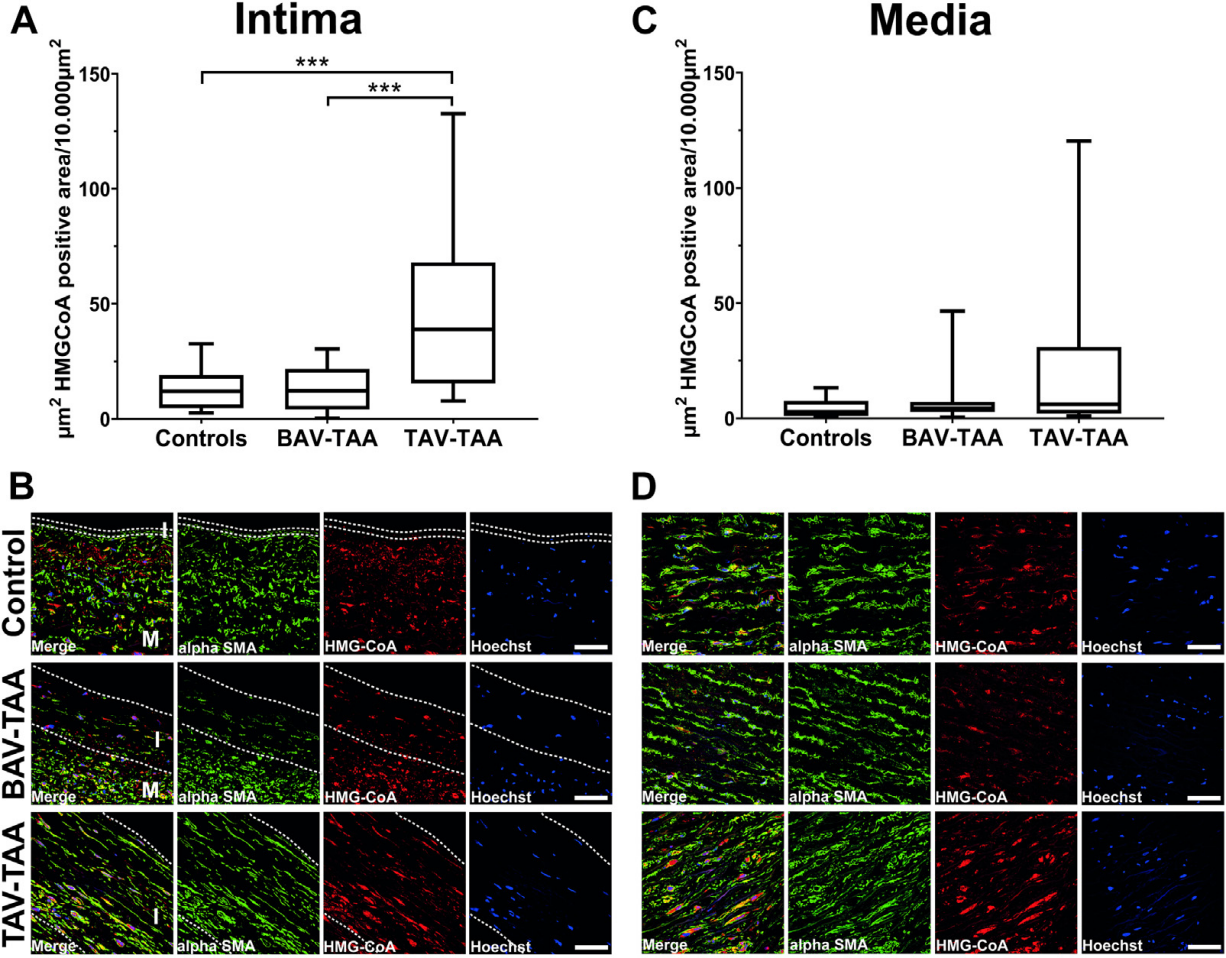
In the intimal layer a higher expression of HMGCR was observed in TAV-TAAs compared to controls and BAV-TAAs. A: Quantification of HMGCR positive area (μm^2^ per 10.000 μm^2^) within the intimal layer of controls, BAV-TAAs, and TAV-TAAs. In (B) representative images of anti-HMGCR staining within the intimal layer is shown; green staining shows alpha smooth muscle actin, red HMGCR staining, and cell nuclei in blue are stained with Hoechst (white dotted lines are indicating the borders of the intima). C: Quantification of HMGCR positive area (μm^2^ per 10.000 μm^2^) in the medial layer of the three samples groups analyzed. D: Depicts representative images of anti-HMGCR staining in the medial layer. White line within the representative images indicates a scale bar of 50 μm. Data are shown as box plots with whiskers. Alpha SMA = alpha smooth muscle actin; I = intimal layer; M = medial layer. Number of different samples analyzed per group are control = 16, BAV-TAA = 17, TAV-TAA=17. ∗∗∗…indicates a *P*-value < 0.001. BAV, bicuspid aortic valve; HMGCR, 3-hydroxy-3-methylglutaryl coenzyme A reductase; TAA, thoracic aortic aneurysm; TAV, tricuspid aortic valve.

HMGCR is a well-known rate-limiting enzyme in cholesterol biosynthesis, which is a major driving force behind atherogenesis and plaque formation. Quantification of protein expression revealed a highly significant increase of HMGCR in the aortic intima of TAV-TAAs. No such accumulation was observed in the control group and in the BAV-TAA group (Fig. 5A and representative images in 5B). Although the TAV-TAA group contains more patients with advanced atherosclerosis, the evaluation per area suggests that this increased expression may be associated with an atypical potentially specific form of atherosclerosis in the TAV-TAAs. In the media, HMGCR expression seems to be increased in the TAV-TAAs but not on a level considered to be significant (control vs. TAV-TAA *P*-value 0.118; BAV-TAA vs. TAV-TAA *P*-value 0.322; see Fig. 5C, D). Nonetheless, this nonsignificant increased expression, together with the MALDI data, showing an accumulation of PCs (see supplemental Fig. S6) in the media, and the detection of increased concentrations of lipids in isolated medial SMCs supports the hypothesis of an impaired lipid metabolism in TAV-TAAs. When comparing the above results, it becomes obvious that patterns on almost all levels of different analytes in BAV-TAAs were very similar compared to the nonaneurysmal controls. In contrast to the different expression levels of HMGCR between groups, protein expression of, e.g., LDL-R and SREBF1 was not altered (see supplemental Figs. S7 and S8).

### Blood plasma lipid concentrations are elevated in TAV-TAAs patients - potential role as a biomarker and target for therapeutic interventions

**Fig. 6:**
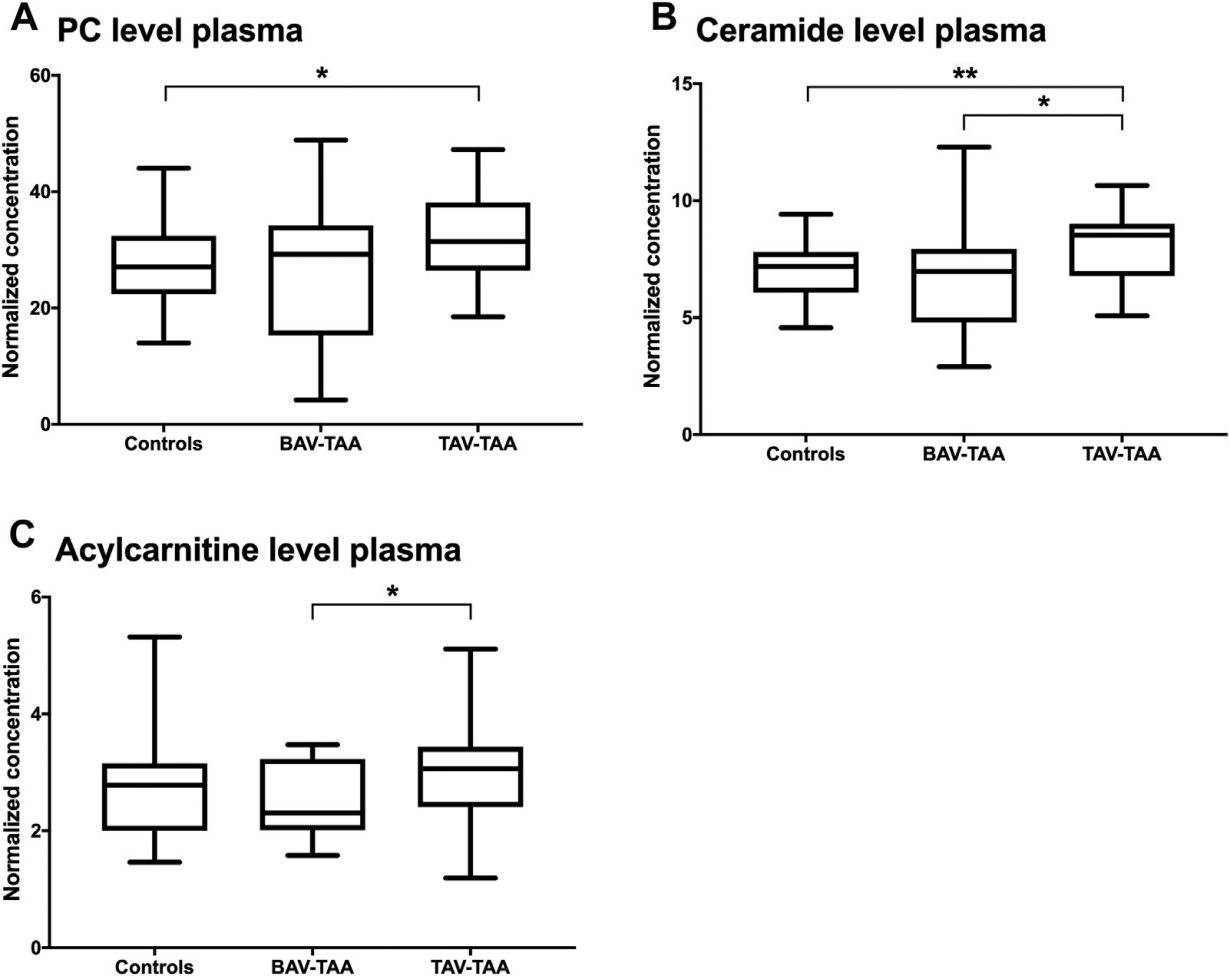
Blood plasma lipids are significantly increased in TAV-TAAs. A: Quantification of PC concentrations in blood plasma of control, BAV-TAA, and TAV-TAA samples (data are shown as normalized concentration). B: Quantification of ceramide concentrations within blood plasma samples collected from the three study groups. C: Quantification of acylcarnitine concentrations within the plasma of control, BAV-TAA, and TAV-TAA samples. Number of samples analyzed per group was control=26, BAV-TAA = 27, TAV-TAA = 27. Data are presented as box blots with median and whiskers. ∗…indicates a *P*-value < 0.05, ∗∗…indicates a *P*-value < 0.01. BAV, bicuspid aortic valve; PC, phosphatidylcholines; TAA, thoracic aortic aneurysm; TAV, tricuspid aortic valve.

Aside from local lipid concentrations, changes in systemic concentrations may serve as a basis for biomarker development and may even constitute a possible target for therapeutic interventions. In order to test for such changes, plasma samples were taken from healthy controls, BAV-TAA, and TAV-TAA patients and subjected to lipid analyses. Plasma from TAV-TAA patients showed, in comparison to controls and BAV-TAA plasma, a significant increase in systemic lipid concentrations. In detail, TAV-TAA plasma samples feature higher levels of PCs (*P* = 0.049) and higher levels of ceramides (*P* = 0.006) than in controls (see Fig. 6A, B). We could also observe higher levels of ACs (*P* = 0.021) in TAV-TAAs plasma than in BAV-TAAs plasma (see Fig. 6C). In summary, in TAV-TAAs elevated levels of lipid concentrations were observable locally in tissue, in isolated medial SMCs, and systemically in plasma. In contrast, no such changes were detected in BAV-TAAs. Plasma lipid analyses have also been performed additionally using nonnormalized data (see supplemental Fig. S9). Of note, plasma content of PCs, ceramides, and ACs do not correlate with LDL, HDL, total cholesterol, cholesterol ratio, and triglyceride levels in the plasma of the patients.

## DISCUSSION

Despite significant clinical improvements in the treatment of TAAs, most therapies still consist of classical conservative treatments and surgical options. This is mainly due to a still significant lack of knowledge about the exact pathomechanisms of these diseases. Without doubt, important elements of disease progression have already been identified. However, the "holy grails," i.e., the disease initiating events, the earliest possible time point of diagnosis and also the therapy to stop disease progression still remains enigmatic. Many renowned researchers have revealed patho-mechanisms (mainly end-stage processes) and biomarkers during recent years, yet many issues regarding the TAA are still unclear (23).

It is the hypothesis of the researchers of this study that the pathogenesis of the large group of degenerative TAAs is not based on a unitary process. Based on previously published data and new data from this study, it is likely that within the group of degenerative aneurysms there are at least two (rather more) distinct groups with different types of pathogenesis. As shown previously, and supported by data from the present study, it seems possible that atherosclerosis is one of the main factors in the development and progression of TAV-TAA. Previously published data already indicated the presence of degenerative/atherosclerotic processes in the TAV-TAA vessel wall: increased numbers of fibroblasts, decreased proliferation, and a lower number of cell nuclei. Analyses of primary isolated aneurysm SMCs revealed similar alterations (11, 19, 24). Further support for this hypothesis stems from correlation analyses between the extent of aortic wall alteration (atherosclerosis) and disease severity (media degeneration) shown in the present study. Importantly, the above applies only to TAV-associated aneurysms, and clearly not to degenerative forms associated with a BAV. In the field of abdominal aortic aneurysms, the role of atherosclerotic changes has long been the subject of intense debate. At present, it is still not entirely clear whether atherosclerosis is a relevant factor in abdominal aortic aneurysm development or whether it is only a different/additional result of the presence of the same risk factors. Nevertheless, antiatherosclerotic therapy represents the gold standard in the treatment of abdominal aortic aneurysms today (24, 25). Similarly, there is also still no consensus on the role of atherosclerosis in the development of TAA. Important previous studies, e.g., by Achneck *et al.*, found that ascending aneurysms were associated with a reduced prevalence of atherosclerotic changes (26). In contrast, Albini *et al.* and Leone *et al.* report on an interconnection between the occurrence of atherosclerosis and the development of TAAs (27, 28). These are just a few examples demonstrating the still controversial situation in the field. Clearly, further research and more studies will help clarify and perhaps diversify this area of research.

The major processes, mechanisms, cell types, and genes involved in atherogenesis, particularly in the coronary arteries, are known today (29, 30, 31). However, a comparison of known atherosclerotic processes with those occurring in TAV-TAA atherosclerosis revealed major differences. For example, there is little accumulation of macrophages, a hallmark of classic atherosclerosis, in the hyperplastic intima of TAV-TAAs. In contrast, a significant accumulation of macrophages in the media of TAV-TAAs could be detected, accompanied by media degeneration, a typical characteristic feature of aneurysms. There are several hypotheses possible regarding the relevant signaling pathways that may cause this nonfocal accumulation of macrophages.

The accumulation of lipids/oxidized lipids represents a characteristic hallmark of atherosclerosis and atherosclerotic lesions. Apart from the well-known intimal accumulation of lipids, we detected, for the first time, an accumulation of LPCs and ACs in medial SMCs isolated from TAV-aneurysmal tissue. These data suggest that atherosclerosis is not only a process occurring in the intima of TAV-TAAs but might also be supported/driven by, potentially SMC inherent, proaneurysmal processes, like lipid synthesis and accumulation in the media. These alterations could then serve as a chemoattractant stimulus for macrophages as has already been reported previously (32). Importantly, the well-known macrophage attractor MCP-1 is rather unlikely to play a role in macrophage attraction to the media. Finally, also the transdifferentiation processes of SMCs to macrophages may be induced by lipids (33). Aside from the source of media residing macrophages, in the sense of classical atherosclerosis, it was already described that macrophages take up oxidized LDL, which induces their differentiation to foam cells. Uncontrolled uptake of oxidized lipids by foam cells may consequently lead to their necrotic cell death and the release of cytosolic constituents into the environment (i.e., the aortic tunica media) (34, 35), followed by inflammatory and degenerative processes. This vicious cycle may potentially drive atherosclerosis and degeneration of the media of TAV-TAAs.

Relevant data supporting the concept of lipid accumulation in the media of TAV-TAAs came from MALDI analyses, which demonstrated that alterations in lipid metabolism do not only occur in TAV-TAA in a quantitative manner in tissues and cells but also in a qualitative manner. Furthermore, MALDI analyses revealed the formation of new metabolic structures or patterns characteristic for the aortas of TAV-TAAs. A surprising result of MALDI imaging was an increased concentration of lipids in the media of TAV-TAAs compared to BAV-TAAs and controls. Previously, Sinapius reported that the presence of lipids in the intima is associated with the occurrence of lipids also in the media. These findings led Sinapius to suggest that this interconnection may be based on a transport process in the arterial vessel wall from the intima to the media (14). Our data on lipid concentrations and distribution may argue for the presence of such a transport system. Clearly, there are many and highly different ways how lipids can be transported in the human body. Linking a yet unknown intra-aortic wall transport system to TAV-TAA formation is highly speculative. In general, it can be stated that the lipid metabolism of the TAV-TAA aorta and in particularly that of the TAV-TAA media seems to be perturbed. Nevertheless, a classical chicken and egg issue remains: either is an alteration of the TAV-TAA wall metabolism the driver and inducer of atherosclerosis or vice versa.

In order to further investigate and confirm a potential lipid transport and accumulation scenario we studied gene expression profiles of medial isolated SMCs. The analysis of isolated SMCs in culture, being completely disconnected from systemic influences, justifies the statement that a cell intrinsic alteration in TAV-TAAs derived SMCs elicits a proatherosclerotic phenotype including lipid accumulation and a deregulated gene expression profile in favor of classical proatherosclerotic genes. These results at least partially argue against the model in which macrophages infiltrate the media. Among the proatherosclerotic genes found to be dysregulated in TAV-TAA SMCs, the genes of the intracellular cholesterol sensors *SREBP* (3.0-fold compared with controls) and *INSIG1* (2.3-fold compared with controls) were upregulated. When the intracellular cholesterol concentration is low, INSIG1 detaches from the SREBP/SCAP complex and the complex migrates to the Golgi apparatus. SREBP is subsequently processed and then functions as a nuclear transcription factor and activates cholesterol synthesis by inducing *HMGCR* (2.1-fold compared to controls) (36, 37). Importantly, also the LDL receptor gene was significantly increased in TAV-TAA SMCs (4.1-fold compared with controls). The LDL receptor plays an essential role in the uptake of cholesterol-rich LDL from blood into cells. Excess cholesterol is stored in this context in the form of lipid droplets within the cell (38, 39). However, it has been shown that nonphysiological overexpression of the *LDLR* gene can lead to increased uptake of LDL and intracellular formation of crystalline lipid/cholesterol deposits in vitro and in vivo (40, 41).

One of the most important enzymes for the formation of cholesterol/cholesterol complexes and thus a target for antiatherosclerotic therapies is HMGCR (42). Interestingly, intimal hyperplasia or atherosclerosis in TAV-TAAs is characterized by significantly higher HMGCR expression than with nonaneurysmal and BAV-TAA atherosclerosis. Apart from the absence of macrophage accumulation in intimal hyperplasia/atherosclerosis, the accumulation of this enzyme is another indication of the occurrence of a noncanonical atherosclerosis in TAV-TAAs. Together with the above-mentioned proatherosclerotic perturbation of gene expression in the isolated SMCs, the slightly increased expression of HMGCR is further evidence for the existence of a noncanonical atherosclerosis accompanied by a pathological lipid metabolism in the media of TAV-TAAs.

Finally, and as a central element of the present study, we also collected plasma samples from the patients enrolled in this study. The main goal of this approach was to also test for possible metabolic changes at the systemic level. We were pleased to see that blood plasma metabolic analyses also showed significant, and proatherosclerotic, lipid disturbances in the plasma of TAV-TAA patients. The observed elevated levels of PCs and ceramides in TAV-TAAs might therefore serve as potential new markers for the TAV-TAA or as a starting point for new therapeutic interventions. However, and maybe even more important, these results combined with metabolomic data from tissue and isolated cells can be used to identify new pathogenic principles, not only in the TAV-TAA.

## LIMITS OF THE STUDY AND CONCLUSION

The data that we report in this manuscript are of descriptive nature and need further confirmation. In addition, the lack of appropriate animal model systems is still a major drawback in TAA research, which also hampers the elucidation of TAA pathophysiology. Nevertheless, by combining a broad spectrum of analyses we could reveal novel conditions existing and coinciding in TAV-TAA disease and progression. Based on own data and data from other experts in the field we are sufficiently convinced to state the following hypothesis for further investigation: BAV-TAAs and TAV-TAAs are diseases with a similar outcome but differ massively in their pathogenesis (hardly any disease processes / phenotypes observed in TAV-TAAs were observed in BAV-TAAs). In TAV-TAAs, lipid-mediated and noncanonical atherosclerotic alterations in both the intima and the media may play relevant roles in disease progression. Of note, particularly patients suffering from TAV-TAAs may profit from new antiatherosclerotic treatments against new, yet unknown TAV-TAA relevant targets.

## Data availability

All data are available upon request by Univ.-Prof. Dr David Bernhard, head of the Division of Pathophysiology, Institute of Physiology and Pathophysiology, Medical Faculty, Johannes Kepler University Linz, Linz, Austria; e-mail: David.Bernhard@jku.at. The gene data set of this study is freely available in GEO NCBI [GSE219204].

## Supplemental data

This article contains supplemental data.

## Conflict of interest

The authors declare that they have no conflicts of interest with the contents of this article.
