# Supplementary material for: Noncanonical atherosclerosis as the driving force in tricuspid aortic valve associated aneurysms - A trace collection
Source: J Lipid Res. 2023 Feb 1;64(3):100338. doi: 10.1016/j.jlr.2023.100338 (PMC10009546; doi:10.1016/j.jlr.2023.100338)
Supplement: Supplemental data [file mmc1.docx]

**Online Supplement to**

**Non-canonical atherosclerosis as the driving force in tricuspid aortic valve-associated aneurysm – a trace collection**

By Doppler C. et al.

**Material and Methods**

Metabolomic analyses of isolated primary cells and plasma samples

Plasma and cell extracts were used for metabolomic analysis. In total, 630 metabolites covering 14 small molecule classes and 12 different lipid classes were analyzed using the MxP Quant 500 kit (Biocrates) following the manufacturer’s protocol. In brief, 10 µl of human plasma were pipetted on a 96 well-plate containing internal standards and dried under a nitrogen stream using a positive pressure manifold. 50µl of a 5% phenyl isothiocyanate (PITC) solution were added to each well to derivatize amino acids and biogenic amines. After 1h incubation time at room temperature, the plate was dried again. To extract the metabolites 300µl 5mM ammonium acetate in methanol were pipetted onto a filter and incubated for 30min. The extract was eluted into a new 96-well plate using positive pressure. FIA-MS/MS was used for acylcarnitines, lipids and hexose, UPLC-MS/MS was used for all other metabolite classes.

For further LC-MS/MS analyses 150 µl of the extract was diluted with an equal volume of water. For FIA-MS/MS analyses 10 µl extract was diluted with 490 µl of FIA solvent (provided by Biocrates). After dilution, LC-MS/MS and FIA-MS/MS measurements were performed. For chromatographical separation an UPLC I-class PLUS (Waters) system was used coupled to a SCIEX QTRAP 6500+ mass spectrometry system in electrospray ionization (ESI) mode. Water with 0.2% formic acid (A) and acetonitrile with 0.2% formic acid were used as eluents. Data was generated using the Analyst (Sciex) software suite and transferred to the MetIDQ software (Biocrates) which was used for further data processing and analysis. All metabolites were identiﬁed using isotopically-labeled internal standards and multiple reaction monitoring (MRM) using optimized MS conditions as provided by Biocrates. For quantification, either a seven-point calibration curve or one point calibration was used depending on the metabolite class.

**Results**

**OLS Table 1: Information on the degree of atherosclerosis and media degeneration of each sample.** Overall, the TAV-TAA group exhibited increased media degeneration as well as increased atherosclerotic changes compared to the BAV-TAA and control groups.

| **Controls** | | |  | **BAV-TAA** | | |  | **TAV-TAA** | | |
| --- | --- | --- | --- | --- | --- | --- | --- | --- | --- | --- |
| **Sample number** | **Grade of media degeneration** | **Grade of atherosclerosis** |  | **Sample number** | **Grade of media degeneration** | **Grade of atherosclerosis** |  | **Sample number** | **Grade of media degeneration** | **Grade of atherosclerosis** |
| C 1 | No | I |  | B 1 | Intermediate | 0 |  | T 1 | Severe | IV |
| C 2 | No | I |  | B 2 | No | I |  | T 2 | No | III |
| C 3 | Intermediate | II |  | B 3 | Minimal | 0 |  | T 3 | Minimal | I |
| C 4 | Minimal | III |  | B 4 | Minimal | I |  | T 4 | Advanced | IV |
| C 5 | Minimal | I |  | B 5 | No | I |  | T 5 | Severe | IV |
| C 6 | Minimal | I |  | B 6 | Advanced | III |  | T 6 | Severe | V |
| C 7 | Minimal | III |  | B 7 | Minimal | I |  | T 7 | Advanced | II |
| C 8 | No | I |  | B 8 | Minimal | I |  | T 8 | Intermediate | V |
| C 9 | Minimal | 0 |  | B 9 | Minimal | IV |  | T 9 | Intermediate | III |
| C 10 | Minimal | I |  | B 10 | No | II |  | T 10 | Intermediate | II |
| C 11 | No | I |  | B 11 | Minimal | II |  | T 11 | Intermediate | II |
| C 12 | Intermediate | II |  | B 12 | No | I |  | T 12 | Minimal | III |
| C 13 | Minimal | 0 |  | B 13 | No | 0 |  | T 13 | Severe | IV |
| C 14 | Minimal | I |  | B 14 | Intermediate | I |  | T 14 | Severe | IV |
| C 15 | Minimal | III |  | B 15 | Minimal | I |  | T 15 | Severe | III |
| C 16 | Intermediate | II |  | B 16 | Intermediate | 0 |  | T 16 | Intermediate | II |
| C 17 | Minimal | 0 |  | B 17 | No | 0 |  | T 17 | Severe | IV |

**
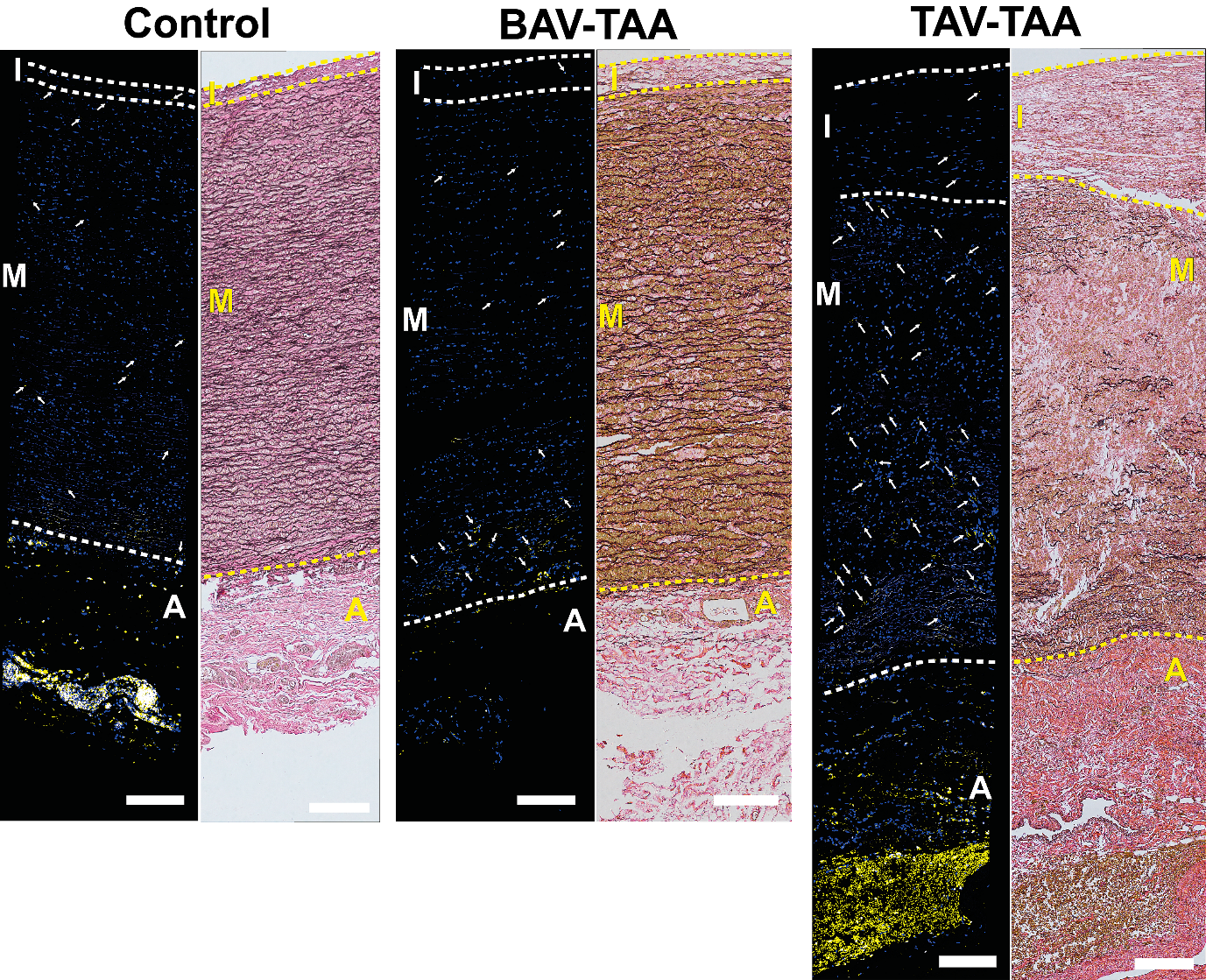
**

**OLS Figure 1 – Macrophage infiltrations are evenly distributed across the media in the TAV-TAA samples.**

The left image of each group (control, BAV-TAA, TAV-TAAA) shows a large image of anti CD68 stained tissues (white arrows indicate CD68 positive cells; white dotted lines are indicating the wall layer borders). The corresponding right image shows Elastika van Gieson stained samples, which allows an easier orientation regarding the different layers (red/pink color indicates collagen, black color indicates elastic fibers and light yellow color indicates muscle; yellow dotted lines are indicating the wall layer borders). The images show the increased presence of macrophages in the aortic media of TAV-TAA samples, compared to control samples and BAV-TAAs. Of note, macrophages are evenly distributed within the layers of the aorta. Scale bar indicates 200µm. Please note, that there is a reuse of images of OLS Figure 1 in Figure 4 and OLS Figure 6. The rationale for the reuse of images is to allow for a better comparison of different analyses and stainings. (I … intima; M … media; A … adventitia)

**OLS Figure 2 – No difference in MCP-1 protein amount between the groups.**


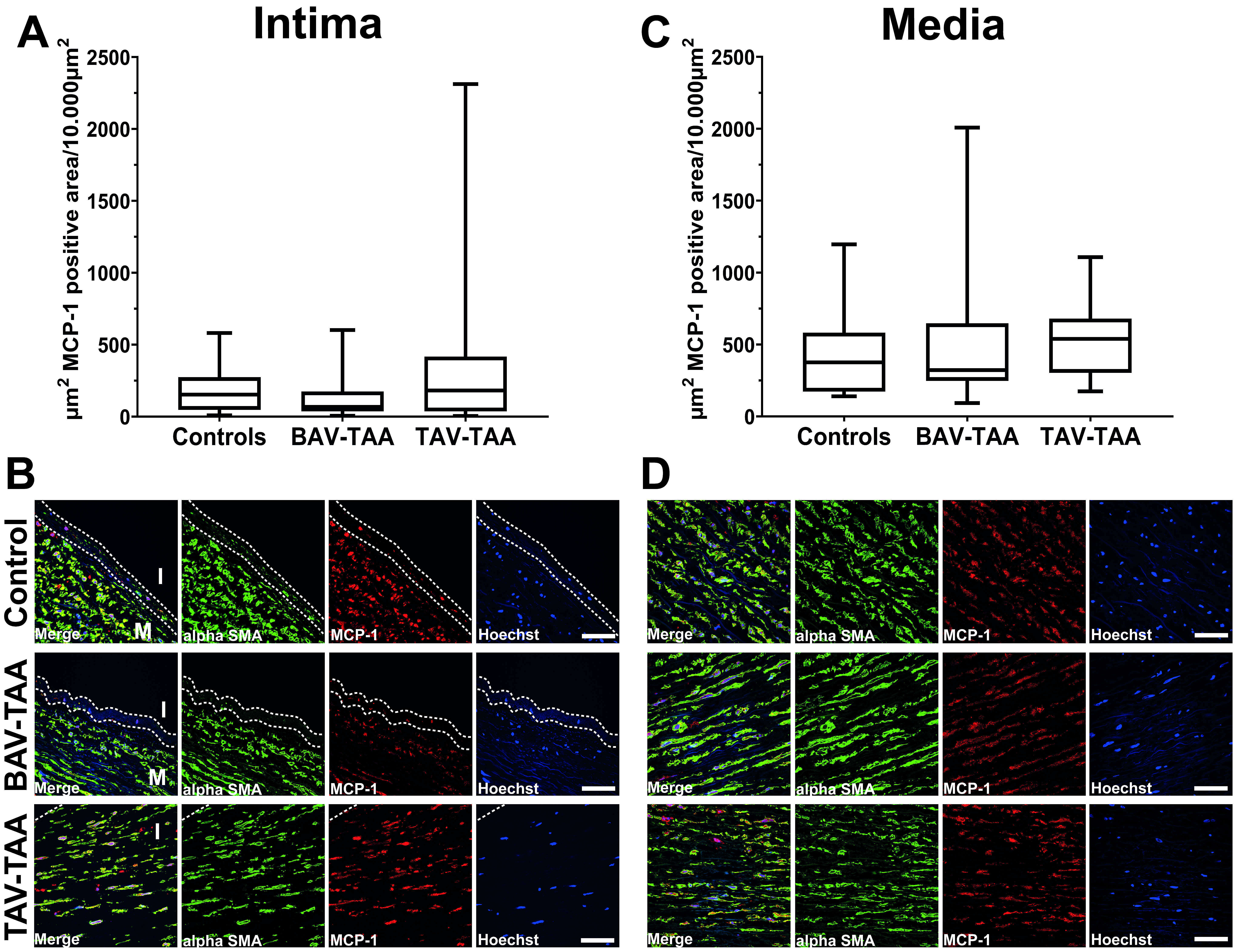


**(A)** Quantification of MCP-1 positive area (µm^2^ per 10.000µm^2^) within the intimal layer of controls, BAV-TAAs and TAV-TAAs. In **(B)** representative images of anti-MCP-1 staining within the intima is shown. In green alpha smooth muscle actin is shown, in red the MCP-1 staining and in blue the cell nuclei stained using Hoechst (alpha SMA = alpha smooth muscle actin; I = intimal layer; M = medial layer). White dotted lines are indicating the borders of the intima. **(C)** Quantification of MCP-1 positive area (µm^2^ per 10.000µm^2^) within the aortic media of the three study groups. In **(D)** representative images of the anti-MCP-1 staining within the aortic media are presented (alpha SMA = alpha smooth muscle actin; I = intimal layer; M = medial layer). White dotted lines are indicating the borders of the intima. Data are shown as box plots with median and whiskers. Number of samples analyzed per group are control=16, BAV-TAA=17, TAV-TAA=17. Scale bar indicates 50µm.

**
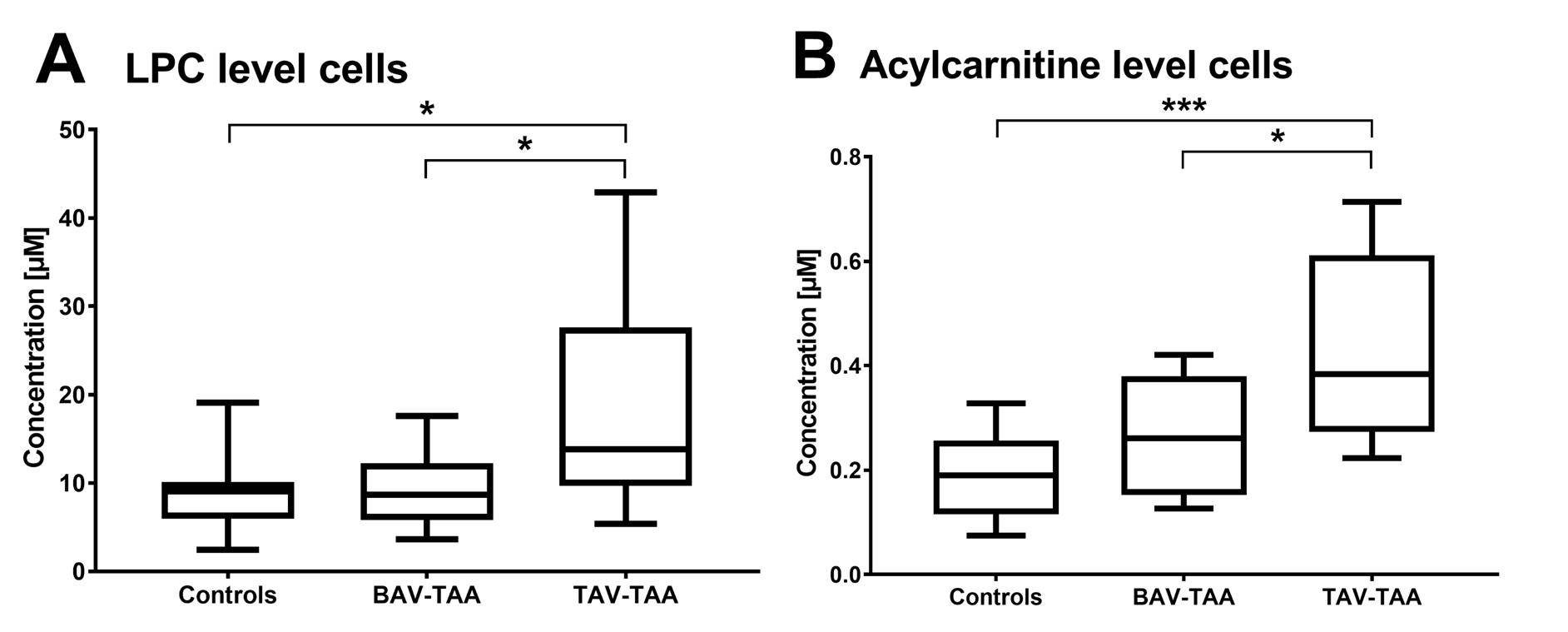
** **OLS Figure 3 – Isolated SMCs from TAV-TAAs hold significantly increased amounts of lysophosphatidylcholines and acylcarnitines compared to BAV TAAs and controls.**

**(A)** Determination of the concentration of lysophosphatidylcholines (LPCs) in isolated SMCs from controls, BAV-TAAs and TAV-TAAs. **(B)** Determination of the concentration of acylcarnitines in isolated smooth muscle cells of the three study groups. Non-normalized data are given. Number of cell lines used for the analysis: control=8, BAV-TAA=10, TAV-TAA=14. Data are given as box blots with median and whiskers. * indicates a p-value <0.05, *** indicates a p-value <0.001.


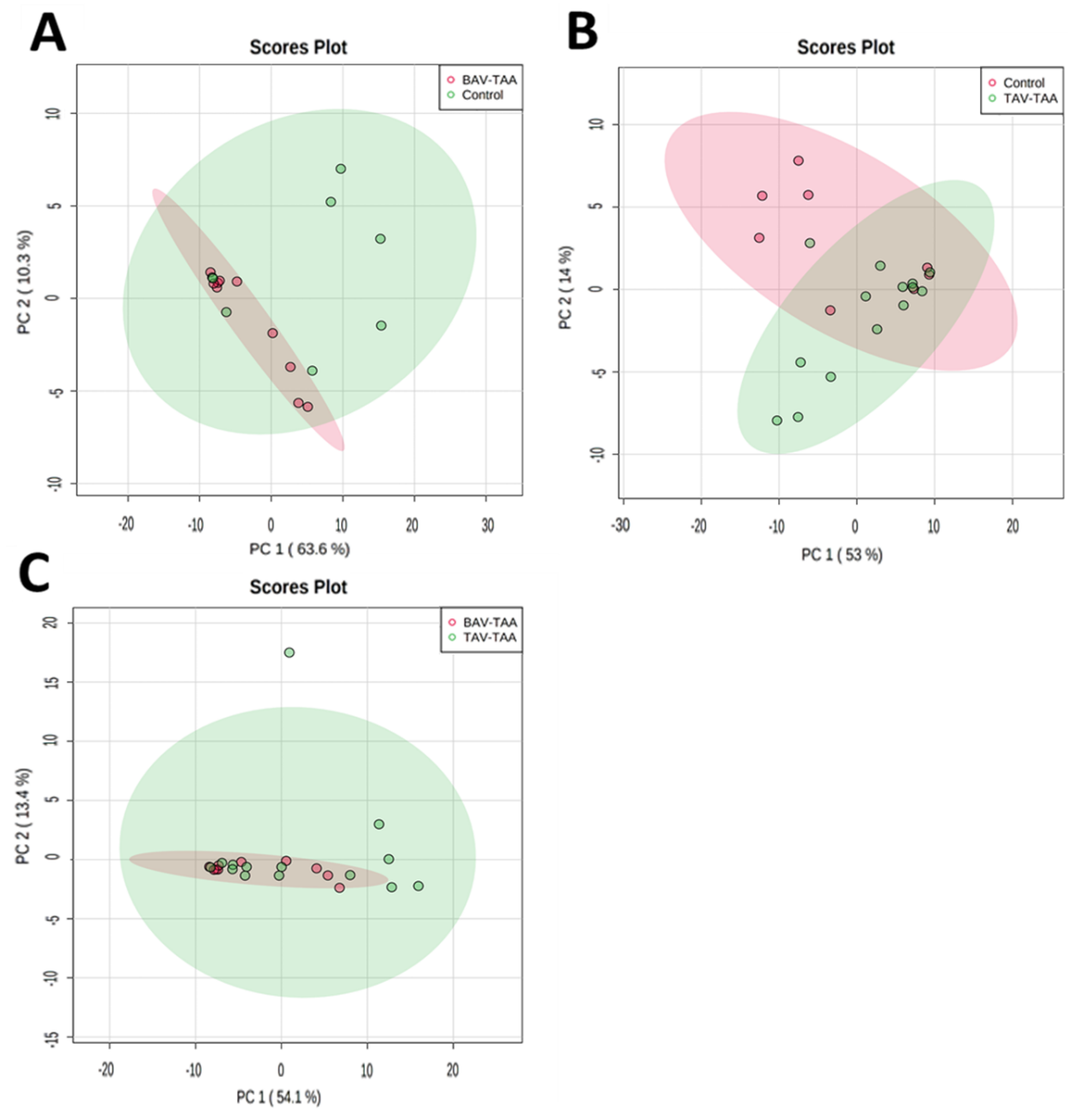


**OLS Figure 4 – Principal component analysis (PCA) allows for the separation of control and BAVs from TAV-TAAs based on variances in lipid concentrations.**

To test for variations in metabolomic profiles between the groups, metabolites were quantified using AbsoluteIDQ1 p150 kit and were grouped into compound classes (i.e. lipids, amino acids). The variance of respective metabolite concentrations between groups was analyzed using PCA (MetaboAnalyst 4.0 was used for PCA of major metabolite classes as lipids, amino acids). Principal components 1 and 2 in datasets containing all lipids described a majority of data variance: 73.9% for control vs. BAV-TAA (see OLS Figure 4A), 67.0% for control vs. TAV-TAA (see Figure OLS 4B) and 67.5% for BAV-TAA vs. TAV-TAA (see Figure OLS 4C).


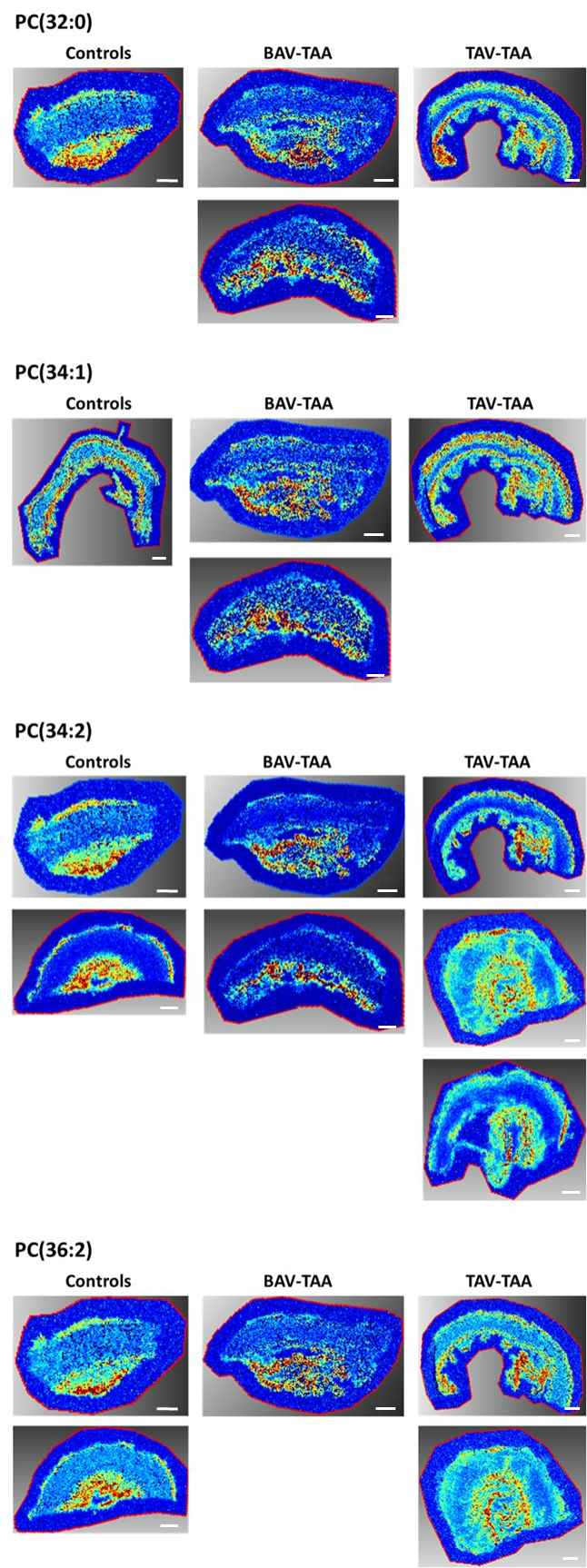


**OLS Figure 5 – MALDI imaging shows specific metabolic patterns in TAV-TAAs allowing for a differentiation from controls and BAV TAAs.**

OLS Figure 5 shows a selection of MALDI images of different analytes of the three groups. MALDI images show that whereas controls and BAV-TAAs have highly similar metabolic patterns throughout the aortic wall, TAV-TAAs show massive perturbations of the aortic wall metabolism and the formation of complex metabolic patterns. The following analytes are depicted: PC(32:0) (C_40_H_80_NO_8_P), PC(34:1) (C_42_H_82_NO_8_P), PC(34:2) (C_42_H_80_NO_8_P), and PC(36:2) (C_44_H_84_NO_8_P). These analytes were identified as protonated molecules and sodium adducts. Lipid concentrations are color-coded with red indicating the highest concentration, blue indicating the lowest/zero concentration. Scale bar indicates 1mm.

**OLS Table 2: List of deregulated genes and metabolic pathways**

OLS Table 2 shows that in the TAV-TAAs a significant number of genes and pathways are deregulated when compared to the control and the BAV-TAA group. Generally, a two-fold change was defined as deregulated. Cholesterol and lipid metabolism were severely impaired in the TAV-TAA group.

| **TAV-TAA vs Control** | | | |
| --- | --- | --- | --- |
| **Gene** | **Metabolic pathway** | **Fold Change** | **p-value** |
| **LPCAT3** | Acyl Chain remodelling of PC | **2,1** | 0,105 |
| **PLA2G4A** | Acyl Chain remodelling of PC | **2,6** | 0,791 |
| **LPCAT4** | Acyl Chain remodelling of PC | **2,2** | 0,392 |
| **CHKB** | Synthesis of PC | **2,2** | 0,025 |
| **SLC22A5** | Carnitine metabolism | **3,2** | 0,078 |
| **FADS1** | alpha-linolenic and linoleic acid metabolism | **2,9** | 0,917 |
| **FADS2** | alpha-linolenic and linoleic acid metabolism | **12,0** | 0,193 |
| **ELOVL1** | alpha-linolenic and linoleic acid metabolism | **2,3** | 0,275 |
| **FDPS** | Cholesterol Metabolism | **2,7** | 0,105 |
| **HMGCR** | Cholesterol Metabolism | **2,1** | 0,088 |
| **PLTP** | Cholesterol Metabolism | **3,2** | 0,298 |
| **LDLR** | Cholesterol Metabolism | **4,1** | 0,044 |
| **NCEH1** | Cholesterol Metabolism | **2,4** | 0,639 |
| **VAPA** | Cholesterol Metabolism | **2,1** | 0,013 |
| **EBP** | Lipid metabolism | **2,5** | 0,165 |
| **FDPS** | Lipid metabolism | **2,7** | 0,105 |
| **HMGCR** | Lipid metabolism | **2,1** | 0,088 |
| **IDI1** | Lipid metabolism | **3,1** | 0,314 |
| **SCD** | Lipid metabolism | **3,9** | 0,132 |
| **PLPP1** | Glycerophospholipid metabolism | **2,3** | 0,676 |
|  |  |  |  |
| **TAV-TAA vs BAV-TAA** | | | |
| **Gene** | **Metabolic pathway** | **Fold Change** | **p-value** |
| LPCAT3 | Acyl Chain remodelling of PC | 1,6 | 0,220 |
| PLA2G4A | Acyl Chain remodelling of PC | 1,6 | 0,612 |
| **LPCAT4** | Acyl Chain remodelling of PC | **4,6** | 0,040 |
| CHKB | Synthesis of PC | 1,5 | 0,072 |
| SLC22A5 | Carnitine metabolism | 1,4 | 0,243 |
| FADS1 | alpha-linolenic and linoleic acid metabolism | 1,2 | 0,439 |
| **FADS2** | alpha-linolenic and linoleic acid metabolism | **3,8** | 0,570 |
| **ELOVL1** | alpha-linolenic and linoleic acid metabolism | **2,9** | 0,014 |
| FDPS | Cholesterol Metabolism | 1,6 | 0,181 |
| HMGCR | Cholesterol Metabolism | 1,6 | 0,094 |
| **PLTP** | Cholesterol Metabolism | **2,0** | 0,250 |
| **LDLR** | Cholesterol Metabolism | **2,2** | 0,450 |
| **NCEH1** | Cholesterol Metabolism | **2,5** | 0,477 |
| VAPA | Cholesterol Metabolism | 1,6 | 0,009 |
| **EBP** | Lipid metabolism | **2,8** | 0,089 |
| FDPS | Lipid metabolism | 1,6 | 0,181 |
| HMGCR | Lipid metabolism | 1,6 | 0,094 |
| IDI1 | Lipid metabolism | 1,1 | 0,480 |
| **SCD** | Lipid metabolism | **2,1** | 0,508 |
| PLPP1 | Glycerophospholipid metabolism | 1,2 | 0,689 |
|  |  |  |  |
|  |  |  |  |
| **BAV-TAA vs Control** | | | |
| **Gene** | **Metabolic pathway** | **Fold Change** | **p-value** |
| LPCAT3 | Acyl Chain remodelling of PC | 1,2 | 0,475 |
| PLA2G4A | Acyl Chain remodelling of PC | 1,6 | 0,222 |
| **LPCAT4** | Acyl Chain remodelling of PC | **-3,2** | 0,386 |
| CHKB | Synthesis of PC | 1,2 | 0,139 |
| SLC22A5 | Carnitine metabolism | 1,8 | 0,311 |
| **FADS1** | alpha-linolenic and linoleic acid metabolism | **2,6** | 0,257 |
| **FADS2** | alpha-linolenic and linoleic acid metabolism | **3,2** | 0,184 |
| ELOVL1 | alpha-linolenic and linoleic acid metabolism | 1,1 | 0,907 |
| FDPS | Cholesterol Metabolism | 1,5 | 0,212 |
| HMGCR | Cholesterol Metabolism | 1,6 | 0,466 |
| PLTP | Cholesterol Metabolism | 1,7 | 0,958 |
| **LDLR** | Cholesterol Metabolism | **2,0** | 0,046 |
| **NCEH1** | Cholesterol Metabolism | **5,8** | 0,212 |
| VAPA | Cholesterol Metabolism | 1,4 | 0,282 |
| EBP | Lipid metabolism | -1,1 | 0,797 |
| FDPS | Lipid metabolism | 1,5 | 0,212 |
| HMGCR | Lipid metabolism | 1,6 | 0,466 |
| **IDI1** | Lipid metabolism | **2,7** | 0,013 |
| SCD | Lipid metabolism | 1,4 | 0,304 |
| PLPP1 | Glycerophospholipid metabolism | 1,9 | 0,471 |


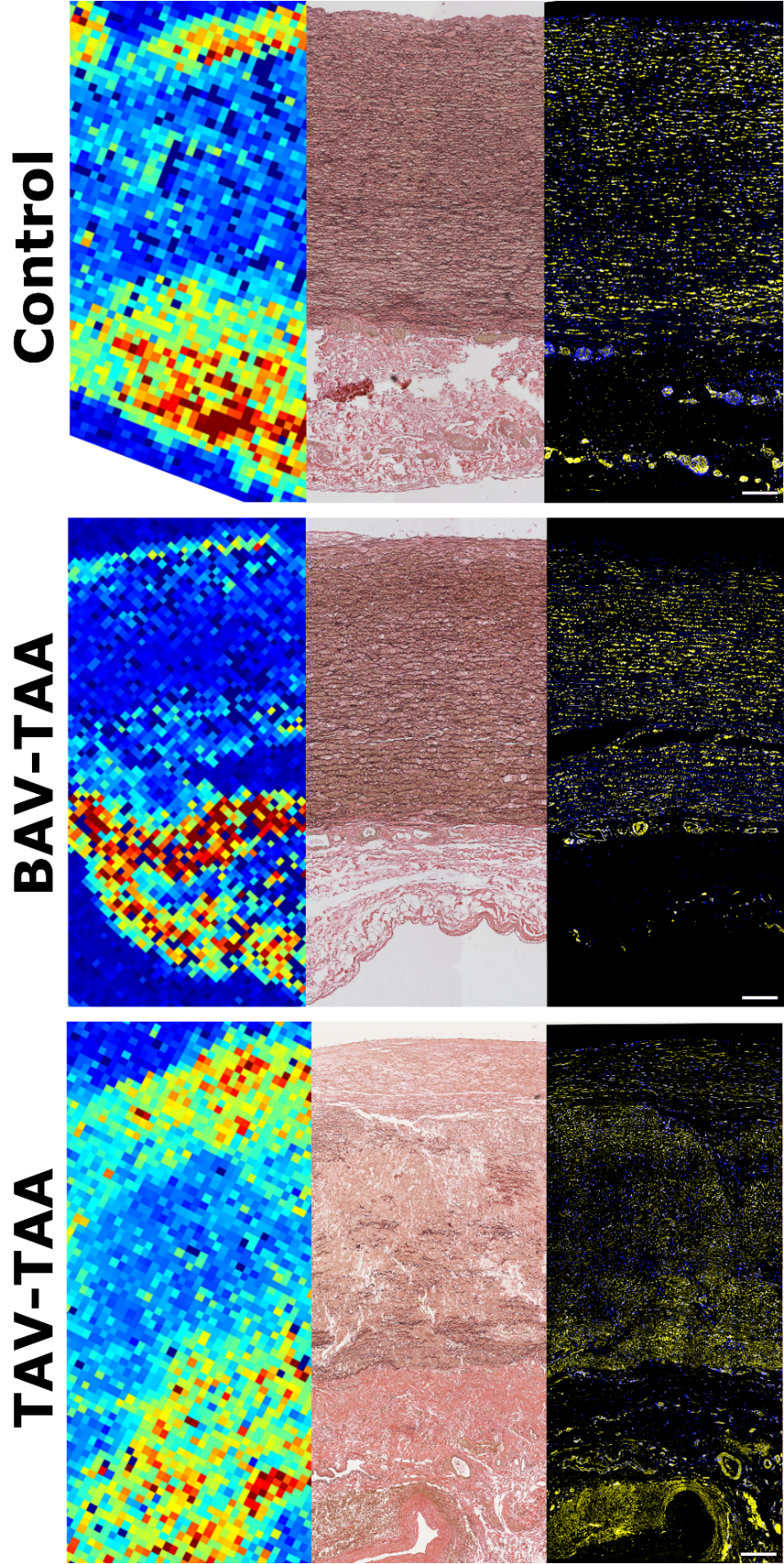


**OLS Figure 6 – Comparison of MALDI images with histological structures and HMGCR protein expression**

The images in the left column show MALDI images of PC(34:2) (C_42_H_80_NO_8_P) identified as protonated molecule (m/z = 769.562 ± 0.125%) and sodium adduct (m/z = 780.551 ± 0.125%, shown here) of control, BAV-TAA and TAV-TAA. Lipid concentrations are color-coded with red indicating the highest concentration, blue indicating the lowest/zero concentration. The middle column shows the corresponding tissue sections stained with Elastica van Gieson to display the layers of the aortic wall. Red/pink color indicates collagen, black color indicates elastic fibers and light yellow color indicates muscle. The right column shows large images of tissue sections stained for HMG-CoA. Yellow color indicates HMG-CoA expression signal; blue color indicates cell nuclei (stained with Hoechst). Large images of HMGCR staining are composed of 5x9 pictures for control and BAV-TAA sections, and 5x11 images for the TAV-TAA group as the thickness of the aortic wall is larger in the TAV group compared to the other two groups. Scale bar indicates 500µm. Please note, that there is a reuse of images of OLS Figure 6 in Figure 4 and OLS Figure 1. The rationale for the reuse of images is to allow for a better comparison of different analyses and stainings.


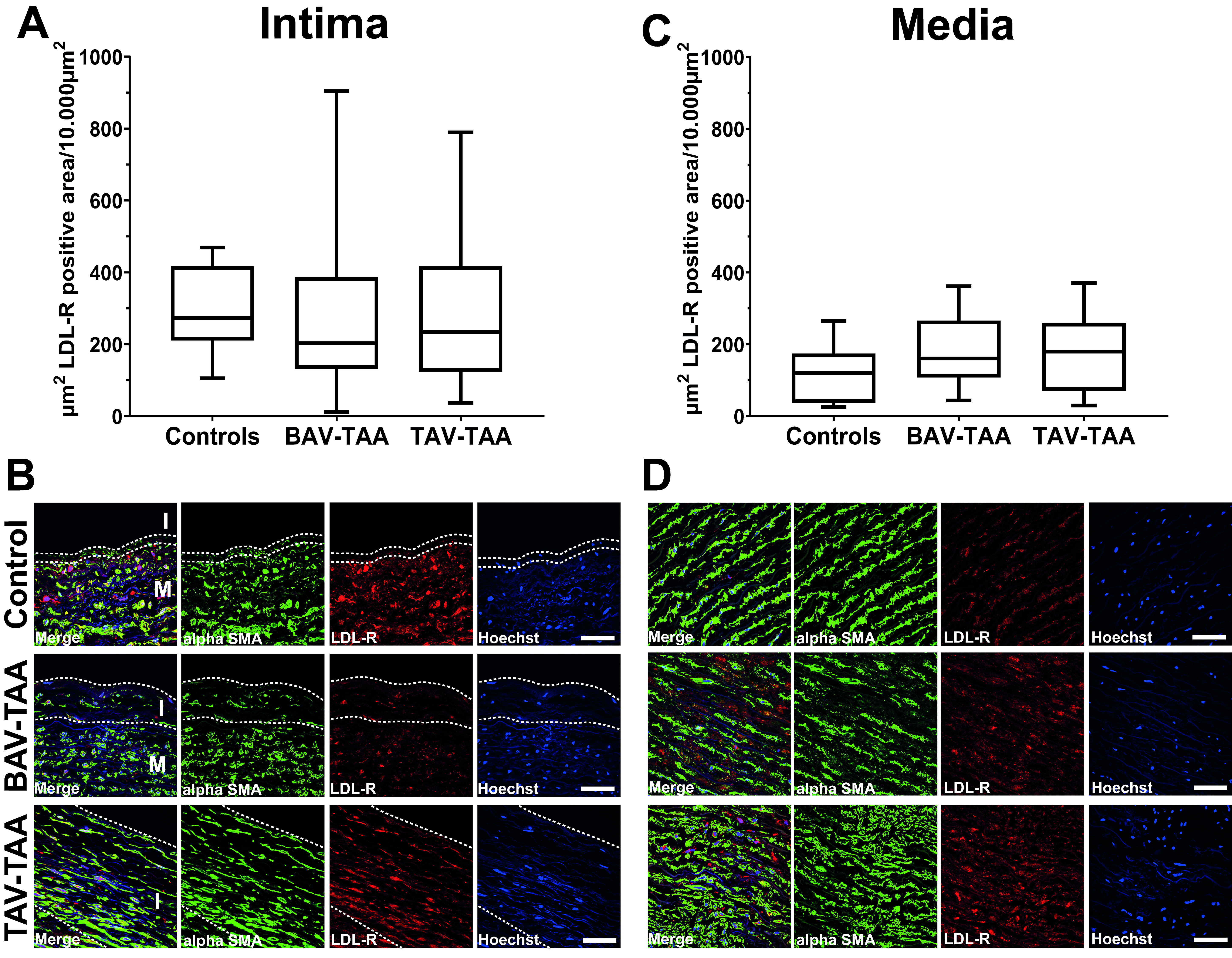


**OLS Figure 7 – In contrast to gene expression, LDL-R protein levels do not differ between the groups**

**(A)** Quantification of LDL-R positive area (µm^2^ per 10.000µm^2^) within the intimal layer of controls, BAV-TAAs and TAV-TAAs. In **(B)** representative images of anti-LDL-R staining within the intima is shown. In green alpha smooth muscle actin is shown, in red the LDL-R expression and in blue the cell nuclei stained using Hoechst (alpha SMA = alpha smooth muscle actin; I = intimal layer; M = medial layer). **(C)** Quantification of LDL-R positive area (µm^2^ per 10.000µm^2^) within the aortic media of the three study groups. In **(D)** representative images of the anti-LDL-R staining within the aortic media are presented (alpha SMA = alpha smooth muscle actin; I = intimal layer; M = medial layer). White dotted lines are indicating the borders of the intima. Data are shown as box plots with median and whiskers. Number of samples analyzed per group are control=16, BAV-TAA=17, TAV-TAA=17. Scale bar indicates 50µm.


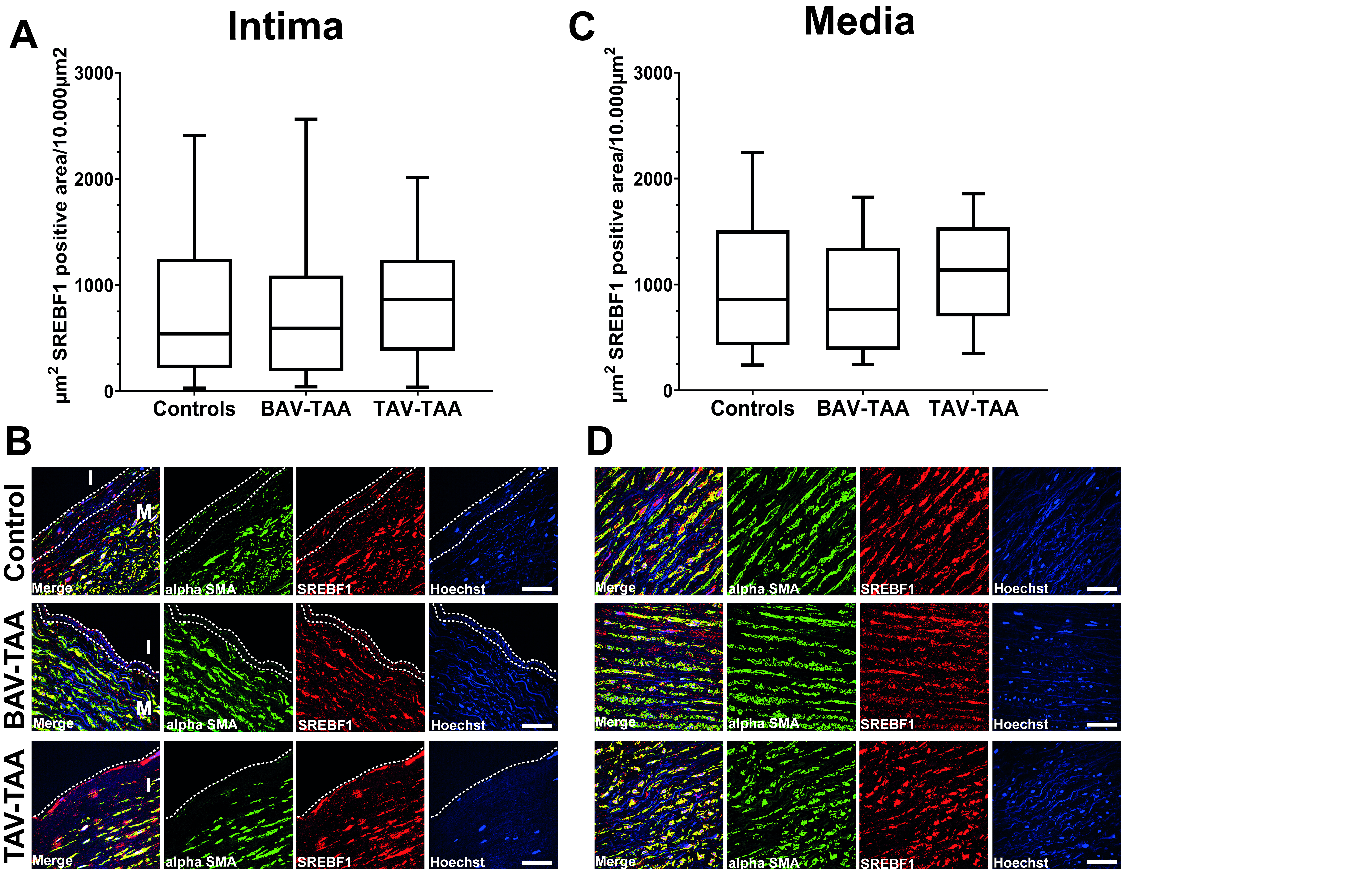


**OLS Figure 8 – In contrast to gene expression, SREBF1 proteins levels do not differ between the groups**

**(A)** Quantification of SREBF1 positive area (µm^2^ per 10.000µm^2^) within the intimal layer of controls, BAV-TAAs and TAV-TAAs. In **(B)** representative images of anti-SREBF1 staining within the intima is shown. In green alpha smooth muscle actin is shown, in red the SREBF1 expression and in blue the cell nuclei stained using Hoechst (alpha SMA = alpha smooth muscle actin; I = intimal layer; M = medial layer). **(C)** Quantification of SREBF1 positive area (µm^2^ per 10.000µm^2^) within the aortic media of the three study groups. In **(D)** representative images of the anti-SREBF1 staining within the aortic media are presented (alpha SMA = alpha smooth muscle actin; I = intimal layer; M = medial layer). White dotted lines are indicating the borders of the intima. White line within the representative images indicates a scale bar of 50µm. Data are shown as box plots with median and whiskers. Number of samples analyzed per group are control=16, BAV-TAA=17, TAV-TAA=17.


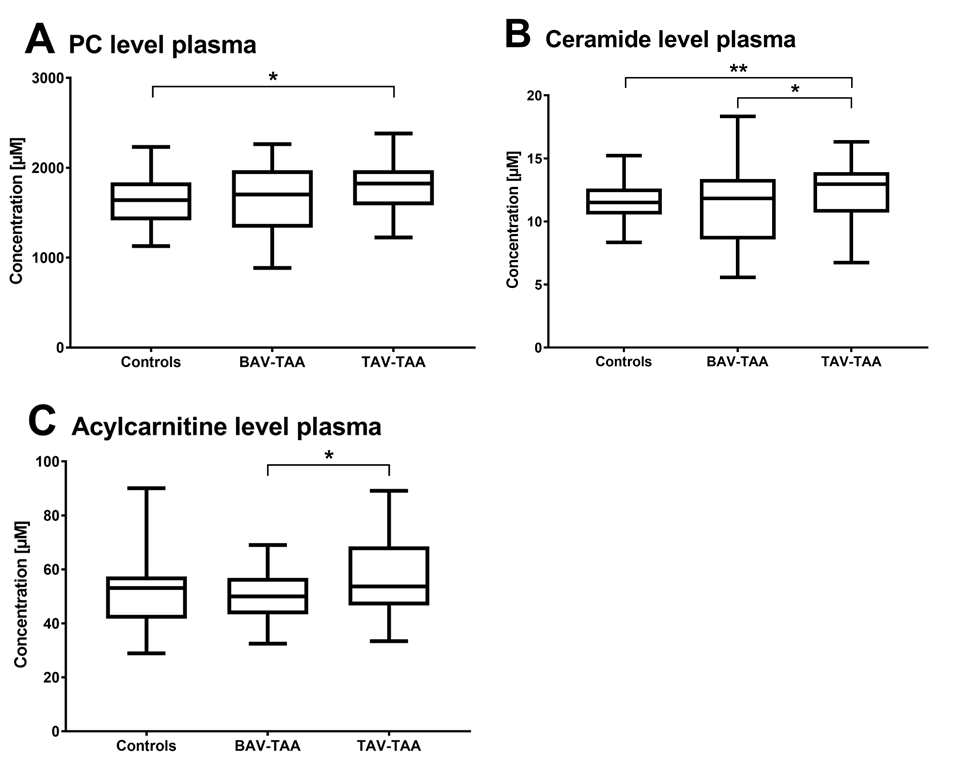


**OLS Figure 9 – Blood plasma from TAA-TAA patients contains significantly higher levels of phosphatidylcholines, ceramides and acylcarnitines in comparison to controls and BAV-TAAs** **patients**

**(A)** Quantification of PC concentrations in blood plasma of control, BAV-TAA, and TAV-TAA samples (data are shown as absolute concentration). **(B)** Quantification of ceramide concentrations within blood plasma samples collected from the three study groups. **(C)** Quantification of acylcarnitine concentrations within the plasma of control, BAV-TAA, and TAV-TAA samples. Number of samples analyzed per group was control=26, BAV-TAA=27, TAV-TAA=27. Data are presented as box blots with median and whiskers. *…indicates a p-value <0.05, **…indicates a p-value <0.01.

**OLS Table 3: List of genes with more than two-fold change in expression and the corresponding p-values**

| *C-BAV* | | | *C-TAV* | | | *BAV-TAV* | | |
| --- | --- | --- | --- | --- | --- | --- | --- | --- |
| **Gene symbol** | **Fold change** | ***p*-value** | **Gene symbol** | **Fold change** | ***p*-value** | **Gene symbol** | **Fold change** | **p-value** |
| A2M | 2,83 | 0,0619 | AADAC | -4,37 | 0,1363 | A2M | 2,64 | 0,1278 |
| AADAC | -2,26 | 0,0767 | AADACL3 | -1,31 | 0,0494 | AADAT | 3,37 | 0,0361 |
| AADACL3 | -1,25 | 0,0487 | AADAT | -3,31 | 0,0836 | AATF | -1,39 | 0,0493 |
| AAGAB | -2,12 | 0,0194 | AAK1 | 2,3 | 0,1270 | ABCC3 | -4,32 | 0,2393 |
| AARS | -2,09 | 0,9122 | AASDH | -2,35 | 0,0136 | ABCC9 | -2,94 | 0,888 |
| AASDH | -2,57 | 0,0007 | AASS | 1,84 | 0,0433 | ABCD4 | -2,25 | 0,3693 |
| ABCA6 | -2,1 | 0,3194 | ABCA8 | -2,21 | 0,7165 | ABHD2 | 2,36 | 0,5795 |
| ABCA8 | -2,03 | 0,5989 | ABCC10 | -2,08 | 0,2928 | ABI3BP | 2,45 | 0,151 |
| ABCC3 | -4,9 | 0,4062 | ABCC9 | -3,19 | 0,0175 | ABL2 | -1,71 | 0,0496 |
| ABCC5 | -1,48 | 0,0089 | ABCD4 | 3 | 0,2396 | ABLIM3 | 6,01 | 0,2139 |
| ABCC9 | -11,21 | 0,0218 | ABCG1 | 2,22 | 0,4649 | ACAD9 | -1,18 | 0,0411 |
| ABHD17AP4 | -1,72 | 0,0443 | ABHD17B | -2,28 | 0,0059 | ACAP2 | 1,73 | 0,0444 |
| ABHD17B | -3,01 | 0,0140 | ABHD17C | 1,69 | 0,0148 | ACE | -2,02 | 0,073 |
| ABHD2 | 2,31 | 0,6852 | ABHD6 | -2,02 | 0,7117 | ACOT13 | 1,96 | 0,0005 |
| ABHD5 | -1,98 | 0,0033 | ABI3BP | -2,1 | 0,0607 | ACOX2 | 2,23 | 0,0614 |
| ABLIM1 | 4,09 | 0,7560 | ABLIM1 | 2,23 | 0,7748 | ACRV1 | 1,31 | 0,0391 |
| ABLIM3 | 3,32 | 0,0289 | ABTB1 | 2,02 | 0,0221 | ACSF2 | 5 | 0,0566 |
| AC063956.1 | 1,61 | 0,0117 | ACAD9 | 1,94 | 0,0442 | ACSL5 | -1,48 | 0,0052 |
| ACAT1 | 1,81 | 0,0388 | ACAP2 | -2,04 | 0,0691 | ACTA2 | 5,49 | 0,7398 |
| ACIN1 | 2,28 | 0,2813 | ACD | 2,24 | 0,4128 | ACTG2 | 2,02 | 0,1499 |
| ACOX2 | 2,76 | 0,0406 | ACE | 2,13 | 0,6918 | ACTN4 | 2,47 | 0,4586 |
| ACSF2 | 2,23 | 0,4507 | ACP6 | -2 | 0,8690 | ACTRT1 | 1,44 | 0,0324 |
| ACSS3 | 2,25 | 0,2805 | ACSF2 | -2,22 | 0,1472 | ACVR2A | -2,14 | 0,1273 |
| ACTA2 | 4,7 | 0,4114 | ACVR2A | 2,47 | 0,6367 | ACYP2 | 3,29 | 0,0029 |
| ADAL | -1,64 | 0,0264 | ACYP2 | -2,22 | 0,0129 | ADAM12 | -3,62 | 0,9742 |
| ADAM19 | -3,4 | 0,3308 | ADAL | -2,36 | 0,0172 | ADAM17 | -2,03 | 0,197 |
| ADAMTS1 | 3,54 | 0,3510 | ADAM12 | 2,67 | 0,7826 | ADAM23 | 1,96 | 0,0209 |
| ADAMTS4 | -2,19 | 0,3297 | ADAM19 | -2,7 | 0,6093 | ADAM32 | 1,94 | 0,0129 |
| ADAMTS7 | 2,3 | 0,1408 | ADAM32 | -3,14 | 0,0267 | ADAMTS12 | 3,61 | 0,0977 |
| ADAMTS9 | -6,53 | 0,0016 | ADAMTS1 | 3,92 | 0,6595 | ADAMTS4 | -2,51 | 0,1311 |
| ADCY6 | 2,06 | 0,4866 | ADAMTS12 | -4,01 | 0,8698 | ADAMTS5 | 2,23 | 0,2245 |
| ADGRF5 | 5,05 | 0,5575 | ADAMTS15 | -2,27 | 0,6919 | ADAMTS7 | 2,52 | 0,2461 |
| ADGRG6 | -3,17 | 0,3709 | ADAMTS5 | -2,88 | 0,1285 | ADAP1 | 1,81 | 0,0066 |
| ADH1B | -9,77 | 0,2476 | ADAMTS9 | -17,92 | 0,5523 | ADARB1 | -2,68 | 0,05 |
| ADIPOR2 | 2,21 | 0,0068 | ADAP1 | -1,4 | 0,0402 | ADAT1 | -2,25 | 0,6517 |
| ADM | 2,58 | 0,2143 | ADAR | 2,2 | 0,2036 | ADGRA2 | -2,14 | 0,4187 |
| ADRBK1 | -2,05 | 0,9026 | ADCY3 | -2,44 | 0,5986 | ADGRE5 | -2,16 | 0,4437 |
| ADTRP | -1,52 | 0,0113 | ADCY9 | 1,82 | 0,0348 | ADGRF5 | 2,9 | 0,2212 |
| AEBP1 | 4,41 | 0,4375 | ADD3 | -1,49 | 0,0326 | ADGRG1 | -6,39 | 0,0638 |
| AEBP2 | 1,41 | 0,0196 | ADGRA2 | 2,86 | 0,4997 | ADGRG6 | -6,39 | 0,4917 |
| AFAP1 | -2,58 | 0,7269 | ADGRA3 | 1,58 | 0,0282 | ADH1B | -28,87 | 0,0086 |
| AFF2 | -4,02 | 0,1045 | ADGRD1 | 3,37 | 0,8481 | AEBP2 | 1,43 | 0,0148 |
| AGO4 | -2,11 | 0,3187 | ADGRG1 | 5 | 0,1897 | AGK | 1,78 | 0,032 |
| AGTR1 | 3,14 | 0,5235 | ADGRG6 | 2,07 | 0,9353 | AGPAT4 | -2,15 | 0,0233 |
| AHCTF1 | -2,66 | 0,1963 | ADGRL4 | -2,15 | 0,8458 | AGTR1 | 6,44 | 0,036 |
| AJUBA | 2,24 | 0,1864 | ADH1B | 3,13 | 0,1089 | AHCYL2 | 1,34 | 0,0235 |
| AK3 | 2,91 | 0,4747 | ADIPOR2 | 2,05 | 0,0141 | AIFM2 | -1,92 | 0,0135 |
| AK4P6 | 2,46 | 0,0246 | ADSSL1 | -2,15 | 0,4625 | AK3 | 2,3 | 0,2358 |
| AK5 | 4,74 | 0,2861 | AEBP1 | 10,37 | 0,8905 | AK4 | 2,44 | 0,159 |
| AKAP6 | 1,32 | 0,0397 | AFAP1 | -2,14 | 0,3993 | AK7 | 1,63 | 0,0499 |
| AKR1C3 | 2,62 | 0,1638 | AFF2 | -3,17 | 0,0599 | AKAP12 | 1,9 | 0,0428 |
| AL596220.1 | 1,39 | 0,0165 | AGBL3 | -1,42 | 0,0412 | AKAP6 | 2,44 | 0,0016 |
| ALCAM | 2,56 | 0,0488 | AGFG1 | 1,69 | 0,0235 | AKAP9 | 2,29 | 0,1494 |
| ALG10 | -3,55 | 0,0279 | AGPAT1 | -2,23 | 0,5883 | AKR1B10 | 2,12 | 0,0127 |
| ALG10B | -1,45 | 0,0327 | AGPAT4 | 2,53 | 0,0187 | AKR1B15 | 1,54 | 0,0251 |
| ANK3 | 2,16 | 0,9706 | AHNAK2 | 2,35 | 0,2422 | AKR1C1 | 2,6 | 0,2838 |
| ANKRD1 | 2,15 | 0,1849 | AICDA | -1,36 | 0,0139 | AKR1C2 | 2,08 | 0,1347 |
| ANKRD18B | 1,65 | 0,0485 | AIP | 2,02 | 0,4487 | ALCAM | 2,31 | 0,0645 |
| ANKRD2 | 1,38 | 0,0391 | AJUBA | 3,7 | 0,2612 | ALS2CR11 | 2,5 | 0,0377 |
| ANKRD28 | 2,35 | 0,6561 | AK5 | 3,2 | 0,4973 | AMMECR1L | -2,8 | 0,0897 |
| ANKRD29 | 2,27 | 0,3485 | AKAP12 | -1,93 | 0,0445 | AMTN | -1,46 | 0,0294 |
| ANKRD30B | 1,77 | 0,0392 | AKAP9 | -2 | 0,2268 | ANAPC13 | 2,06 | 0,1909 |
| ANLN | -2,93 | 0,7798 | AKR1C1 | -5,29 | 0,2089 | ANAPC7 | -1,57 | 0,0284 |
| ANOS1 | -2,53 | 0,1120 | AL161784.1 | 2,21 | 0,0949 | ANGPT1 | 2,11 | 0,152 |
| ANXA10 | -8,59 | 0,6055 | AL596220.1 | 1,67 | 0,0054 | ANGPTL2 | -2,22 | 0,1767 |
| AOX1 | 12,71 | 0,0572 | ALDH18A1 | -2,08 | 0,4111 | ANK1 | 2,09 | 0,0119 |
| AP1S3 | 2,18 | 0,0247 | ALG10 | -2,68 | 0,3137 | ANK2 | 2,19 | 0,2484 |
| AP3S1 | 1,36 | 0,0187 | ALKBH2 | -1,36 | 0,0314 | ANK3 | 2,57 | 0,5615 |
| APCS | -1,33 | 0,0366 | ALOX12 | -1,36 | 0,0202 | ANKRD13C | 1,45 | 0,0223 |
| APOBEC3C | 2,84 | 0,0916 | ALS2CR11 | -3,57 | 0,0373 | ANKRD2 | 1,41 | 0,0246 |
| APOBEC3F | 2,05 | 0,1394 | AMOT | 3,01 | 0,0381 | ANKRD49 | -1,27 | 0,0392 |
| AQP1 | 2,78 | 0,1692 | AMPD2 | -2,07 | 0,6024 | ANLN | -2,59 | 0,4623 |
| ARFGEF3 | 1,48 | 0,0229 | ANGEL1 | 2 | 0,3232 | ANPEP | -4,75 | 0,265 |
| ARHGAP11B | -3,14 | 0,3899 | ANGPTL2 | 3,41 | 0,0604 | ANXA10 | 2,21 | 0,4277 |
| ARHGAP32 | -2,33 | 0,4169 | ANK1 | -2,33 | 0,1997 | ANXA3 | -3,35 | 0,05 |
| ARHGDIB | 2,89 | 0,0381 | ANKAR | -1,32 | 0,0446 | AOX1 | 5,02 | 0,1135 |
| ARHGEF17 | 2,23 | 0,1846 | ANKEF1 | -1,41 | 0,0159 | AP2A2 | -1,81 | 0,0159 |
| ARHGEF25 | 3,03 | 0,4348 | ANKH | 2,06 | 0,3833 | AP3S1 | 2,12 | 0,0055 |
| ARHGEF6 | 2,01 | 0,6464 | ANKRD1 | 2,69 | 0,5238 | APBB1IP | -2,63 | 0,0498 |
| ARL2BP | 2,07 | 0,0180 | ANKRD13A | 2,11 | 0,2573 | APELA | -1,4 | 0,0421 |
| ARL3 | 1,38 | 0,0326 | ANKRD52 | 2,09 | 0,1782 | APH1A | -2,44 | 0,13 |
| ARL4A | -1,82 | 0,0306 | ANOS1 | -2,23 | 0,0072 | APLP1 | 3,43 | 0,3135 |
| ARL4AP2 | -2,36 | 0,0373 | ANPEP | 3,71 | 0,6228 | APOA4 | -1,78 | 0,0032 |
| ARMC6 | -2,01 | 0,8297 | ANXA10 | -14,57 | 0,5252 | APOBEC3B | -5,22 | 0,2451 |
| ARRDC1-AS1 | 2,63 | 0,1117 | ANXA3 | 2,3 | 0,2247 | APOBEC3C | 2,41 | 0,7001 |
| ARSEP1 | 1,26 | 0,0464 | AOX1 | 3,65 | 0,6473 | APOM | 1,49 | 0,0245 |
| ASAH1 | 2,18 | 0,0100 | AP1S3 | 3,23 | 0,4702 | AQP1 | 2,02 | 0,1958 |
| ASCC1 | 2,35 | 0,0457 | AP4E1 | -2,01 | 0,4172 | AQR | -2,06 | 0,0551 |
| ASNS | -3,85 | 0,1183 | APBA1 | -1,45 | 0,0234 | AR | 2,21 | 0,1115 |
| ATAD2B | -2,23 | 0,0939 | APC | 1,46 | 0,0294 | ARF4 | 1,64 | 0,0212 |
| ATAD5 | -3,42 | 0,5438 | APIP | -2,15 | 0,2679 | ARG2 | -3,42 | 0,0106 |
| ATE1 | 2,12 | 0,0948 | APLP1 | -2,69 | 0,4758 | ARHGAP1 | 2,07 | 0,2294 |
| ATF5 | -1,64 | 0,0296 | APOA1 | -1,79 | 0,0008 | ARHGAP11B | -3,45 | 0,2353 |
| ATG4D | -2,14 | 0,6477 | APOA4 | 1,78 | 0,0332 | ARHGAP19-SLIT1 | -3,06 | 0,1033 |
| ATP8B1 | 2,64 | 0,1840 | APOBEC3B | 2,84 | 0,3343 | ARHGAP20 | -3,71 | 0,1332 |
| ATR | -2,2 | 0,2891 | AQP11 | -1,41 | 0,0139 | ARHGAP31 | -2,07 | 0,0859 |
| AURKB | 2,01 | 0,7720 | AR | -2,25 | 0,8265 | ARHGAP33 | -1,68 | 0,0134 |
| AVPR1A | -1,28 | 0,0380 | AREG | -1,45 | 0,0247 | ARHGDIB | 2,66 | 0,6975 |
| AZIN1 | -2,69 | 0,0144 | ARG2 | 3,01 | 0,1411 | ARHGEF17 | 2,3 | 0,2286 |
| B3GALT2 | 6,06 | 0,2093 | ARHGAP20 | 6,83 | 0,0327 | ARHGEF25 | 6,41 | 0,359 |
| B4GALT2 | -2,0 | 0,9576 | ARHGAP33 | 1,65 | 0,0115 | ARHGEF3 | 2,39 | 0,8918 |
| B4GALT6 | -2,37 | 0,5102 | ARHGAP42 | -2,75 | 0,0320 | ARHGEF35 | 2,45 | 0,2102 |
| BANP | 3,8 | 0,5186 | ARHGAP5 | -2,09 | 0,2693 | ARHGEF39 | -1,92 | 0,0473 |
| BBS9 | 2,03 | 0,1747 | ARHGEF39 | 2,93 | 0,2585 | ARHGEF9 | 2,16 | 0,1486 |
| BCAR1 | 2,26 | 0,1694 | ARID5B | -2,52 | 0,5375 | ARL1 | 1,67 | 0,0013 |
| BCAS2 | -2,04 | 0,3542 | ARL1 | -1,57 | 0,0277 | ARL16 | 1,39 | 0,0278 |
| BCAT1 | -2,15 | 0,4273 | ARL14EP | -1,73 | 0,0305 | ARL3 | 1,29 | 0,0236 |
| BCKDHB | 2,32 | 0,0446 | ARL2BP | 1,42 | 0,0482 | ARL5A | 2,03 | 0,1598 |
| BCL11A | -2,35 | 0,2746 | ARL5A | -2,13 | 0,0449 | ARL6 | 2,02 | 0,2932 |
| BCL2L12P1 | -1,36 | 0,0295 | ARL6 | -2,53 | 0,1548 | ARL6IP1 | -2,5 | 0,2676 |
| BCL6 | 2,05 | 0,4609 | ARL6IP6 | 3,37 | 0,0460 | ARL6IP4 | -1,53 | 0,0364 |
| BDH2 | 2,53 | 0,1429 | ARMC10 | -1,39 | 0,0483 | ARL6IP6 | -5,39 | 0,0145 |
| BDKRB1 | 5,04 | 0,0553 | ARMC8 | 2,07 | 0,1698 | ARL8B | 1,32 | 0,0218 |
| BDNF | 2,72 | 0,0245 | ARRB1 | 4,89 | 0,2300 | ARL9 | 2,92 | 0,0001 |
| BEND6 | -3,05 | 0,0052 | ARRDC1-AS1 | 2,73 | 0,0397 | ARMC4 | 1,62 | 0,0107 |
| BEX1 | 3,13 | 0,1493 | ARRDC3 | 2,01 | 0,0525 | ARMC9 | 2,36 | 0,2978 |
| BID | 1,83 | 0,0306 | ASCC3 | -2,21 | 0,0484 | ARMCX2 | 2,38 | 0,9339 |
| BIRC6 | 2,05 | 0,0410 | ASF1B | 3,54 | 0,2627 | ARPP21 | 2,08 | 0,0588 |
| BLM | -2,5 | 0,4361 | ASPM | 2,81 | 0,9243 | ARRB1 | -3,04 | 0,1135 |
| BLOC1S3 | -2,1 | 0,3464 | ASPN | -2,56 | 0,0338 | ARRDC1 | -1,88 | 0,0028 |
| BMP6 | -2,79 | 0,5551 | ASTE1 | 1,68 | 0,0303 | ARRDC4 | 2,03 | 0,6352 |
| BMS1P15 | -2,3 | 0,0056 | ATE1 | 2,71 | 0,1387 | ARSB | 2,41 | 0,0324 |
| BMX | -1,58 | 0,0372 | ATF2 | 1,96 | 0,0265 | ASCC3 | 1,91 | 0,0206 |
| BRCA2 | -2,66 | 0,2921 | ATF6B | 2 | 0,1006 | ASF1B | -2,21 | 0,3465 |
| BST2 | -3,35 | 0,0087 | ATF7IP2 | 1,34 | 0,0181 | ASH2L | -1,9 | 0,0233 |
| BTBD1 | 1,88 | 0,0285 | ATG10 | -1,92 | 0,0255 | ASNS | -4,2 | 0,3969 |
| BTBD7 | -2,09 | 0,6107 | ATG13 | 2,21 | 0,3118 | ASPM | -6,11 | 0,4949 |
| BTF3L4 | -1,59 | 0,0259 | ATL2 | 2,48 | 0,1821 | ASPN | 2,65 | 0,0461 |
| BTG3 | -2,47 | 0,4867 | ATM | -2,02 | 0,1470 | ASPRV1 | 1,53 | 0,0074 |
| BTN2A2 | 3,18 | 0,0054 | ATOX1 | -1,82 | 0,0199 | ASS1 | -2,03 | 0,7963 |
| BTN3A2 | 3,64 | 0,1140 | ATP10D | -2,34 | 0,8024 | ASXL1 | -2,03 | 0,2463 |
| BUB1B | -2,69 | 0,3206 | ATP11A | 3,07 | 0,1219 | ATAD5 | -2,75 | 0,4856 |
| BVES | -3,04 | 0,0897 | ATP13A2 | -2,35 | 0,5228 | ATG2B | 1,92 | 0,0103 |
| C1QTNF3 | -1,52 | 0,0146 | ATP2A1 | 1,41 | 0,0357 | ATG3 | 1,76 | 0,0311 |
| C1QTNF7 | -1,31 | 0,0377 | ATP6V0B | -2,62 | 0,6089 | ATG4D | -2,37 | 0,1542 |
| CA12 | 2,35 | 0,0896 | ATP6V1C1 | 2,14 | 0,1046 | ATM | 2,27 | 0,0014 |
| CA14 | 1,22 | 0,0312 | ATP6V1G1 | -1,7 | 0,0186 | ATOX1 | 2,05 | 0,0412 |
| CACHD1 | 2,52 | 0,1150 | ATPIF1 | -2,11 | 0,1372 | ATP11A | -2,53 | 0,1025 |
| CACNA1A | 4,53 | 0,0284 | AURKB | 16,61 | 0,2753 | ATP1B4 | -1,22 | 0,032 |
| CACNA1C | 2,11 | 0,2983 | AVEN | -2,07 | 0,6673 | ATP2A3 | -2,06 | 0,6288 |
| CACNB4 | 3,19 | 0,0021 | AXL | 2,13 | 0,2156 | ATP5SL | -2,29 | 0,1336 |
| CACYBP | -3,29 | 0,5669 | AZIN1 | -1,49 | 0,0464 | ATP6V1C2 | -1,23 | 0,032 |
| CAD | 2,34 | 0,1096 | AZIN2 | -3,96 | 0,6749 | ATP6V1G1 | 1,45 | 0,0436 |
| CALB1 | -1,71 | 0,0018 | B3GALT6 | -1,31 | 0,0248 | ATPIF1 | 2,63 | 0,0799 |
| CALD1 | 4,86 | 0,0267 | BAG2 | -1,61 | 0,0194 | ATXN10 | 1,18 | 0,0428 |
| CALHM2 | -1,49 | 0,0115 | BANP | 4,74 | 0,0326 | ATXN2L | -1,73 | 0,0037 |
| CALM3 | 2,15 | 0,1469 | BBOF1 | -2,63 | 0,0268 | AURKB | -7,56 | 0,0964 |
| CAMK2N1 | -3,09 | 0,2459 | BBX | -1,3 | 0,0494 | AVPI1 | -2,02 | 0,0596 |
| CAMTA2 | 2,25 | 0,2614 | BCAS3 | 2,36 | 0,0144 | AZIN2 | 3,61 | 0,4109 |
| CAND2 | 1,34 | 0,0459 | BCL11A | -2,78 | 0,0665 | B3GALT2 | 4,97 | 0,1274 |
| CAPG | -2,75 | 0,6895 | BCL2A1 | 3,94 | 0,4276 | B4GALT6 | -2,22 | 0,0491 |
| CARD16 | 2,97 | 0,3931 | BCL6 | 2,56 | 0,1104 | BAG5 | -1,74 | 0,0261 |
| CASC1 | -1,61 | 0,0126 | BCS1L | 3,42 | 0,0508 | BARD1 | -2,28 | 0,2813 |
| CASK | 1,45 | 0,0102 | BDH2 | 2,56 | 0,2339 | BASP1 | 1,35 | 0,0295 |
| CBLN3 | 2,26 | 0,0304 | BDKRB1 | 3,33 | 0,4934 | BBIP1 | 1,55 | 0,023 |
| CBS | -7,33 | 0,0550 | BEND6 | -1,64 | 0,0467 | BBOF1 | 2 | 0,0599 |
| CBX6 | 2,18 | 0,1806 | BET1L | -1,87 | 0,0229 | BBS9 | 2,02 | 0,1549 |
| CBY1 | -2,07 | 0,7214 | BEX1 | -2,32 | 0,1215 | BCAT1 | -2,34 | 0,4839 |
| CCBE1 | 2,24 | 0,2576 | BHLHE41 | -7,77 | 0,0263 | BCL2A1 | -10,1 | 0,4511 |
| CCDC126 | -3,12 | 0,6010 | BLOC1S5 | -2,05 | 0,7906 | BCL9 | 1,87 | 0,0313 |
| CCDC15 | -2,88 | 0,3427 | BMP4 | 4,59 | 0,2274 | BCS1L | -2,44 | 0,0215 |
| CCDC152 | -2,09 | 0,2204 | BMP6 | 2,4 | 0,5025 | BEND7 | -2,26 | 0,0118 |
| CCDC179 | 1,57 | 0,0412 | BNC2 | 3,7 | 0,1501 | BET1 | 2,17 | 0,0143 |
| CCDC185 | 1,37 | 0,0351 | BNIP1 | -1,39 | 0,0484 | BET1L | 1,98 | 0,015 |
| CCDC58 | -2,1 | 0,5793 | BORCS5 | 1 | 0,0384 | BEX1 | 7,75 | 0,0068 |
| CCDC68 | -3,26 | 0,0369 | BRAP | -1,18 | 0,0402 | BFAR | -1,56 | 0,032 |
| CCDC77 | -2,52 | 0,0588 | BRCA1 | -2,11 | 0,5378 | BGN | 2,37 | 0,6194 |
| CCL2 | 2,22 | 0,2096 | BRD8 | 2,14 | 0,0200 | BHLHE41 | 6 | 0,0132 |
| CCNA2 | -3,16 | 0,6633 | BRIP1 | -2,21 | 0,8814 | BIRC6 | 1,64 | 0,0241 |
| CCNB1 | -3,83 | 0,9341 | BRSK1 | -1,29 | 0,0496 | BLM | -2,69 | 0,6257 |
| CCNB2 | -2,06 | 0,7904 | BRWD1 | -1,57 | 0,0212 | BLOC1S1-RDH5 | 2,14 | 0,1177 |
| CCND1 | 5,91 | 0,1937 | BTBD8 | -1,67 | 0,0276 | BLOC1S2 | 1,7 | 0,043 |
| CCND2-AS1 | -1,53 | 0,0466 | BTN2A2 | 4,53 | 0,0127 | BMP4 | -2,04 | 0,4456 |
| CCNDBP1 | 1,79 | 0,0041 | BTN3A2 | 2,56 | 0,0908 | BMP5 | 1,45 | 0,0394 |
| CCPG1 | -1,86 | 0,0115 | BUB3 | 2,18 | 0,1005 | BMP6 | -6,58 | 0,1891 |
| CCT4P2 | -1,45 | 0,0310 | BZRAP1 | -1,25 | 0,0301 | BNC2 | -2,35 | 0,6864 |
| CD14 | -2,13 | 0,1546 | BZW1 | 2,51 | 0,1673 | BRAT1 | -1,86 | 0,0092 |
| CD163L1 | 2,59 | 0,2844 | C10orf107 | -3,49 | 0,1298 | BRE | 2 | 0,3341 |
| CD24 | -13,83 | 0,1281 | C10orf2 | 2,15 | 0,0698 | BST2 | -2,65 | 0,0619 |
| CD248 | 5,31 | 0,8005 | C12orf75 | -2,78 | 0,4026 | BTBD8 | 1,68 | 0,0338 |
| CD320 | -2,32 | 0,8302 | C17orf62 | -2,01 | 0,6328 | BTBD9 | -2,39 | 0,5978 |
| CD46 | 2,78 | 0,0038 | C18orf25 | 2,18 | 0,1724 | BTG3 | -3,4 | 0,6216 |
| CD55 | -3,62 | 0,0775 | C18orf32 | -2,37 | 0,0198 | BUB1B | -7,1 | 0,1042 |
| CDC14B | 2,02 | 0,0017 | C18orf54 | 2,95 | 0,4133 | BZW1 | -2,04 | 0,3517 |
| CDC25C | -2,04 | 0,2502 | C1D | -2,62 | 0,0778 | C10orf12 | 2,01 | 0,2793 |
| CDC42EP3 | 2,53 | 0,0548 | C1GALT1 | -2,72 | 0,0930 | C10orf2 | -2,55 | 0,5074 |
| CDC7 | -3,18 | 0,1312 | C1orf112 | 3,56 | 0,1841 | C11orf1 | 2,82 | 0,0075 |
| CDCA2 | -3,36 | 0,5655 | C1orf123 | 2,47 | 0,0609 | C11orf70 | 2,75 | 0,0434 |
| CDCA5 | 2,49 | 0,8555 | C1QTNF6 | -2,16 | 0,9617 | C12orf75 | 3,21 | 0,185 |
| CDH10 | 3,51 | 0,0898 | C1R | 6,19 | 0,5072 | C14orf37 | -2,09 | 0,1221 |
| CDH13 | 2,28 | 0,7143 | C1RL | 2,54 | 0,1296 | C17orf53 | -2,35 | 0,649 |
| CDH2 | -2,29 | 0,4059 | C1S | 3,12 | 0,8442 | C18orf54 | -2,39 | 0,2525 |
| CDH6 | 2,15 | 0,6295 | C2CD5 | 2,06 | 0,0287 | C1orf112 | -4,87 | 0,1124 |
| CDIPT-AS1 | -1,59 | 0,0200 | C4orf46 | 2,14 | 0,1316 | C1orf21 | -3,02 | 0,1193 |
| CDK1 | -2,28 | 0,7980 | C5orf28 | -2,9 | 0,0465 | C1orf53 | 3,09 | 0,0334 |
| CDK11B | -2,03 | 0,0050 | C6orf52 | -2,81 | 0,1755 | C1R | -15,47 | 0,2918 |
| CDK15 | 1,82 | 0,0269 | CA5B | -5,72 | 0,1518 | C1S | -13,22 | 0,5836 |
| CDK2 | 2,3 | 0,3582 | CA9 | -3,2 | 0,2302 | C20orf27 | -2,17 | 0,1387 |
| CDK7 | 2,33 | 0,1529 | CAB39L | -1,96 | 0,0313 | C2orf68 | -2,13 | 0,0996 |
| CDKN1A | 5,03 | 0,3720 | CABLES1 | 2,13 | 0,0429 | C3 | -5,72 | 0,7751 |
| CDON | -3,13 | 0,1905 | CABP5 | 1,31 | 0,0401 | C4orf22 | 2,36 | 0,1488 |
| CDY14P | 1,3 | 0,0210 | CABYR | 2,39 | 0,8032 | C4orf32 | -2,6 | 0,009 |
| CDYL | -1,58 | 0,0084 | CACHD1 | 2,21 | 0,0379 | C4orf46 | -2,11 | 0,2181 |
| CELF2 | 2,16 | 0,0162 | CACNA2D1 | -2,82 | 0,0927 | C5 | -2,47 | 0,2359 |
| CELSR1 | -1,47 | 0,0429 | CACNB1 | -2,12 | 0,8551 | C5orf30 | 2,49 | 0,0009 |
| CENPE | -2,26 | 0,3999 | CAD | 2,63 | 0,2398 | C9orf64 | -2,58 | 0,1635 |
| CENPJ | -3,4 | 0,0940 | CALB1 | -1,46 | 0,0121 | CA1 | 1,34 | 0,0432 |
| CENPO | 2,2 | 0,8092 | CALM3 | 2,56 | 0,0752 | CA5B | 3,72 | 0,2174 |
| CENPU | -3,1 | 0,6829 | CAMK2N1 | -7,21 | 0,1495 | CA9 | 2,39 | 0,701 |
| CEP44 | -2,06 | 0,0561 | CAMTA2 | 2,09 | 0,1361 | CAB39L | 2,47 | 0,0857 |
| CEP55 | -4,54 | 0,4838 | CARD16 | 7,91 | 0,1680 | CABLES1 | -3,63 | 0,0881 |
| CEP63 | -1,39 | 0,0349 | CASC1 | -1,5 | 0,0312 | CACNA1A | 3,36 | 0,0328 |
| CEP78 | -2,16 | 0,4651 | CASP2 | 2,81 | 0,1350 | CACNA2D1 | 4,09 | 0,0529 |
| CFAP54 | 1,58 | 0,0139 | CASP8AP2 | 2,04 | 0,6234 | CACNB4 | 2,29 | 0,0239 |
| CFL1P7 | 1,5 | 0,0288 | CASR | 1,32 | 0,0461 | CALD1 | 4,57 | 0,1054 |
| CHAC1 | -8,03 | 0,3013 | CATSPER3 | 2,02 | 0,0013 | CAMK2G | 1,49 | 0,0286 |
| CHAC2 | -2,35 | 0,5514 | CAV1 | 2 | 0,0764 | CAMK2N1 | 2,05 | 0,3099 |
| CHEK2 | 2,34 | 0,0015 | CBLB | 2,07 | 0,0558 | CAMKV | 1,25 | 0,0457 |
| CHFR | -2,14 | 0,7535 | CBLN3 | 2,57 | 0,0808 | CAMLG | 1,51 | 0,0132 |
| CHST3 | -2,27 | 0,2904 | CBX6 | 2,21 | 0,1687 | CAP2 | 3,01 | 0,0081 |
| CITED2 | 2,56 | 0,0474 | CC2D1B | 2,49 | 0,3120 | CARD10 | -1,44 | 0,0221 |
| CKAP2L | -2,34 | 0,5726 | CCDC126 | -2,35 | 0,7147 | CARD16 | -3,25 | 0,2529 |
| CLCA2 | -2,18 | 0,0560 | CCDC144NL | -2,15 | 0,0655 | CASP2 | -2,38 | 0,2006 |
| CLCN6 | 1,71 | 0,0237 | CCDC152 | -2,55 | 0,2272 | CATSPER3 | -1,39 | 0,0215 |
| CLDN1 | 5,33 | 0,0121 | CCDC174 | -1,64 | 0,0284 | CBLL1 | -1,63 | 0,0289 |
| CLDN10 | -6,9 | 0,0923 | CCDC43 | -1,9 | 0,0224 | CBS | -4,6 | 0,0551 |
| CLDN11 | 33,9 | 0,0578 | CCDC68 | -2,75 | 0,1993 | CC2D1B | -2,53 | 0,055 |
| CLEC2B | -2,64 | 0,0590 | CCDC7 | -1,5 | 0,0343 | CCAR2 | -2,27 | 0,1037 |
| CLIC2 | -2,94 | 0,0749 | CCDC73 | 1,64 | 0,0215 | CCDC109B | -2,55 | 0,4332 |
| CLIP4 | 2,03 | 0,0455 | CCDC79 | 1,62 | 0,0240 | CCDC115 | 1,62 | 0,0263 |
| CLK3 | 2,52 | 0,0953 | CCDC83 | -1,31 | 0,0289 | CCDC129 | 1,53 | 0,003 |
| CLK4 | 4,3 | 0,0746 | CCIN | -3,28 | 0,2389 | CCDC174 | 1,35 | 0,023 |
| CLLU1OS | -1,39 | 0,0435 | CCL2 | -2,07 | 0,1798 | CCDC175 | -1,3 | 0,0417 |
| CLPP | -2,2 | 0,9382 | CCL5 | 1,38 | 0,0337 | CCDC186 | 1,33 | 0,0381 |
| CLRN1 | 1,51 | 0,0158 | CCNB2 | 3,21 | 0,7739 | CCDC43 | 1,99 | 0,0494 |
| CLSPN | -2,89 | 0,2604 | CCNC | 2,73 | 0,3251 | CCL11 | 1,28 | 0,0259 |
| CLU | -3,08 | 0,1252 | CCND2 | -3,56 | 0,2665 | CCL2 | 3,55 | 0,0059 |
| CMAHP | -1,69 | 0,0309 | CCNE2 | 2,7 | 0,6158 | CCL20 | 3,04 | 0,065 |
| CMBL | 3,0 | 0,1012 | CCNF | 5,15 | 0,0896 | CCNA2 | -6,7 | 0,3207 |
| CMIP | -2,63 | 0,8001 | CCNK | -2,06 | 0,2322 | CCNB1 | -4,86 | 0,492 |
| CMTR2 | 2,35 | 0,5735 | CCP110 | 2,28 | 0,1537 | CCNB2 | -5,89 | 0,5367 |
| CNGA4 | -1,23 | 0,0405 | CCT3 | 2,18 | 0,2007 | CCND1 | 2,96 | 0,3345 |
| CNKSR2 | -2,15 | 0,3599 | CD24 | -57,29 | 0,1430 | CCND2 | 4,43 | 0,3817 |
| CNN3 | 2,29 | 0,0288 | CD248 | 5,82 | 0,6449 | CCNE2 | -2,76 | 0,4169 |
| CNTN3 | 2,72 | 0,0190 | CD2BP2 | 1,54 | 0,0354 | CCNF | -5,06 | 0,0217 |
| CNTNAP3B | -2,05 | 0,3360 | CD3E | -1,53 | 0,0285 | CCNJ | -1,57 | 0,0192 |
| COBL | -1,24 | 0,0213 | CD44 | -2,05 | 0,2590 | CCNJL | 1,92 | 0,0077 |
| COG2 | 2,0 | 0,0205 | CD58 | -2,08 | 0,0652 | CCT5 | -1,79 | 0,0478 |
| COL4A1 | 3,56 | 0,1139 | CDAN1 | -1,94 | 0,0076 | CD24 | 3,02 | 0,5515 |
| COL5A1 | -5,63 | 0,9000 | CDC16 | 2 | 0,0424 | CD300A | 1,23 | 0,0288 |
| COL6A1 | 2,45 | 0,2493 | CDC20 | 6,06 | 0,1834 | CD3E | 1,49 | 0,046 |
| COL6A2 | 3,52 | 0,3766 | CDC25B | 3,59 | 0,0715 | CD44 | 2,88 | 0,214 |
| COL8A1 | 2,02 | 0,0424 | CDC25C | 2,19 | 0,6242 | CD55 | -3,7 | 0,1158 |
| COLEC12 | -1,83 | 0,0257 | CDC37L1 | -1,38 | 0,0373 | CDAN1 | 1,76 | 0,0042 |
| COPB1 | 1,74 | 0,0226 | CDC40 | 1,74 | 0,0414 | CDC14A | -2,57 | 0,049 |
| COPG2 | 1,53 | 0,0319 | CDC42EP2 | 2,19 | 0,3422 | CDC20 | -5,13 | 0,1594 |
| COPRS | 2,36 | 0,1070 | CDCA5 | 5,15 | 0,4478 | CDC25A | -2,14 | 0,1076 |
| COQ2 | 2,38 | 0,1041 | CDCA8 | 6,18 | 0,1402 | CDC25B | -3,95 | 0,036 |
| COTL1 | 5,24 | 0,1336 | CDH10 | -3,24 | 0,0824 | CDC25C | -6,78 | 0,1045 |
| COX5BP2 | -1,78 | 0,0404 | CDH13 | 2,92 | 0,6723 | CDC42 | -1,2 | 0,0487 |
| COX7A1 | 4,03 | 0,1163 | CDK2 | 6,01 | 0,1111 | CDC45 | -3,31 | 0,0898 |
| COX7B2 | -1,24 | 0,0379 | CDK2AP1 | -1,57 | 0,0132 | CDC6 | -3,23 | 0,1838 |
| CPA4 | -2,03 | 0,5084 | CDK5RAP3 | 2,13 | 0,2241 | CDC7 | -3,55 | 0,2656 |
| CPM | 2,77 | 0,7220 | CDKAL1 | 1,84 | 0,0136 | CDCA2 | -3,21 | 0,2491 |
| CR1 | 1,25 | 0,0413 | CDKL1 | 2,72 | 0,1088 | CDCA5 | -3,26 | 0,2757 |
| CRABP2 | -2,02 | 0,1603 | CDKL5 | 1,66 | 0,0152 | CDCA7L | -2,65 | 0,2796 |
| CREB5 | 2,13 | 0,0964 | CDKN1A | 2,86 | 0,3616 | CDCA8 | -3,74 | 0,1 |
| CREG2 | -1,39 | 0,0484 | CDKN1B | 2,01 | 0,0036 | CDH10 | 8,65 | 0,005 |
| CRHBP | 15,89 | 0,1337 | CDKN2AIP | 2,18 | 0,0880 | CDH2 | -2,68 | 0,7274 |
| CRHR1-IT1 | 2,62 | 0,6734 | CDKN3 | 3,29 | 0,7562 | CDH6 | 3,51 | 0,144 |
| CRIP2 | -2,22 | 0,2463 | CDNF | -2,36 | 0,0228 | CDK2 | -2,45 | 0,1415 |
| CRISPLD2 | 1,79 | 0,0246 | CDON | -4,57 | 0,1329 | CDK4 | -2,12 | 0,1366 |
| CRYAB | 2,88 | 0,4058 | CEMIP | 8,42 | 0,5548 | CDK5RAP1 | -1,9 | 0,0301 |
| CRYGC | -1,49 | 0,0276 | CENPM | 10,86 | 0,0842 | CDKAL1 | -1,66 | 0,0336 |
| CSNK1G2-AS1 | 1,42 | 0,0230 | CENPO | 3,9 | 0,3578 | CDKL1 | -3,76 | 0,0341 |
| CSNK2A1 | -1,45 | 0,0359 | CENPU | -2,66 | 0,7979 | CDKN3 | -3,63 | 0,6029 |
| CSRNP1 | -2,37 | 0,5464 | CEP120 | 1,36 | 0,0339 | CDNF | 1,62 | 0,0155 |
| CST1 | 5,78 | 0,0460 | CEP135 | 2 | 0,0876 | CDR1 | 1,55 | 0,0073 |
| CST2 | 2,64 | 0,1515 | CEP290 | -1,74 | 0,0490 | CDV3 | -1,57 | 0,0187 |
| CST4 | 3,66 | 0,0813 | CEP44 | -2,21 | 0,0304 | CDYL2 | -2,4 | 0,0022 |
| CSTA | -2,58 | 0,0077 | CEP95 | -1,97 | 0,0078 | CELA3B | 1,41 | 0,0266 |
| CTBP2 | -2,04 | 0,6581 | CERK | 2,05 | 0,2166 | CELSR1 | -1,41 | 0,0492 |
| CTC-338M12.9 | 1,69 | 0,0130 | CERS3 | -1,28 | 0,0301 | CEMIP | -5,51 | 0,8798 |
| CTC-490E21.11 | 2,08 | 0,0726 | CFAP43 | -1,49 | 0,0102 | CEND1 | 1,23 | 0,0269 |
| CTDSP2 | -2,16 | 0,6637 | CFAP52 | -1,7 | 0,0253 | CENPC | -1,48 | 0,0377 |
| CTDSPL2 | -2,27 | 0,0262 | CFI | 11,08 | 0,1872 | CENPE | -2,69 | 0,5244 |
| CTHRC1 | -3,17 | 0,4543 | CH25H | 2,38 | 0,3631 | CENPK | -3,29 | 0,4809 |
| CTR9 | 2,4 | 0,0552 | CHAF1B | 5,79 | 0,2689 | CENPM | -7,38 | 0,0499 |
| CTSS | 2,97 | 0,5642 | CHCHD3 | 2,13 | 0,1619 | CENPO | -2,64 | 0,3448 |
| CTTNBP2NL | 1,57 | 0,0348 | CHD4 | -1,73 | 0,0159 | CENPP | -2,41 | 0,1287 |
| CTXN3 | -1,57 | 0,0157 | CHEK2 | 2 | 0,0840 | CENPU | -2,08 | 0,7455 |
| CUTA | 3,12 | 0,1113 | CHKB | 2,17 | 0,0246 | CENPW | -2,17 | 0,6467 |
| CXCR5 | 1,33 | 0,0436 | CHPF | -4,84 | 0,8238 | CEP128 | -2,04 | 0,1102 |
| CYB5R4 | 2,23 | 0,0007 | CHRFAM7A | 1,95 | 0,0114 | CEP135 | -1,81 | 0,0448 |
| CYBRD1 | 3,92 | 0,0010 | CHSY3 | -3,04 | 0,0252 | CEP55 | -3,63 | 0,33 |
| CYCSP49 | 1,42 | 0,0264 | CIR1 | -1,82 | 0,0274 | CEP78 | -3,49 | 0,2413 |
| CYP1B1 | -2,58 | 0,2496 | CKAP2L | -2,57 | 0,9634 | CERS6 | 2,71 | 0,146 |
| CYP21A2 | 1,34 | 0,0294 | CLCA2 | 3,03 | 0,4909 | CES4A | -1,64 | 0,0157 |
| CYP2R1 | -4,59 | 0,0013 | CLCN6 | 2,25 | 0,0186 | CFAP36 | 1,47 | 0,0126 |
| CYP2S1 | 5,42 | 0,3306 | CLDN10 | -7,41 | 0,1447 | CFAP58 | 1,36 | 0,0384 |
| CYP51A1 | 1,62 | 0,0135 | CLDN11 | 20,19 | 0,0102 | CFDP1 | -1,3 | 0,0331 |
| DAAM1 | -2,03 | 0,4387 | CLDN12 | -2,85 | 0,5176 | CFHR3 | -1,62 | 0,02 |
| DACH1 | -5,78 | 0,0386 | CLDN14 | -1,59 | 0,0471 | CFI | -24,93 | 0,0069 |
| DAW1 | 2,09 | 0,4002 | CLDN15 | 2,46 | 0,0741 | CHAC1 | -6,27 | 0,1084 |
| DAZ4 | 1,55 | 0,0127 | CLDN18 | -1,37 | 0,0357 | CHAF1B | -4,64 | 0,2173 |
| DAZAP2 | 1,68 | 0,0492 | CLIC2 | -2,28 | 0,4923 | CHCHD10 | 1,45 | 0,0236 |
| DBF4 | -2,26 | 0,4714 | CLIP4 | 2,8 | 0,0942 | CHD1 | -2,07 | 0,1427 |
| DCAF12L1 | -1,41 | 0,0175 | CLK4 | 2,03 | 0,4695 | CHEK1 | -3,37 | 0,0485 |
| DCAF15 | -2,11 | 0,1721 | CLMN | 2,34 | 0,1353 | CHM | 1,95 | 0,045 |
| DCHS1 | -2,57 | 0,2831 | Clostridiales-1 | -1,6 | 0,0460 | CHMP2B | 1,73 | 0,0036 |
| DCLK1 | 2,43 | 0,4495 | CLPB | 2,47 | 0,2407 | CHPF | 2,43 | 0,4131 |
| DCLRE1A | -2,34 | 0,0013 | CLU | 2,32 | 0,0820 | CHST11 | 2,95 | 0,1054 |
| DCPS | -2,36 | 0,5870 | CMBL | 2,08 | 0,7330 | CHST3 | -2,04 | 0,0391 |
| DDA1 | 2,09 | 0,2330 | CMTR2 | 3,18 | 0,0824 | CHSY3 | 4,29 | 0,007 |
| DDAH1 | 1,61 | 0,0290 | CNIH1 | -2,45 | 0,1788 | CIAPIN1 | -2,07 | 0,0846 |
| DDHD2 | 2,34 | 0,1308 | CNKSR2 | -2,49 | 0,0674 | CIR1 | 1,66 | 0,0028 |
| DDIAS | -2,22 | 0,1126 | CNNM3 | 1,35 | 0,0311 | CIT | -3,09 | 0,1913 |
| DDX43 | -2,76 | 0,2922 | CNOT6L | 3,43 | 0,0259 | CKAP2 | -3,5 | 0,3896 |
| DEFB107B | 1,48 | 0,0247 | CNOT7 | -2,23 | 0,3404 | CKAP4 | 2,49 | 0,0002 |
| DEFB134 | -1,26 | 0,0135 | CNPY3 | -2,01 | 0,6686 | CKLF | -2,15 | 0,8884 |
| DENND5B | 1,5 | 0,0073 | CNTNAP1 | 2,55 | 0,0635 | CKS1B | -3,33 | 0,4891 |
| DEPDC1 | -2,67 | 0,7002 | COBL | -1,34 | 0,0326 | CKS2 | -2,93 | 0,5975 |
| DEPTOR | 4,33 | 0,5397 | COBLL1 | -3,25 | 0,3939 | CLCA2 | -3,22 | 0,0565 |
| DFNA5 | -3,22 | 0,7893 | COG1 | -1,22 | 0,0269 | CLCN4 | -1,71 | 0,0454 |
| DGUOK-AS1 | 1,53 | 0,0294 | COG5 | -1,58 | 0,0358 | CLDN1 | 4,85 | 0,0049 |
| DHCR24 | 3,42 | 0,1379 | COL18A1-AS1 | -1,43 | 0,0090 | CLDN12 | 2,65 | 0,578 |
| DHRS1 | 1,77 | 0,0231 | COL4A4 | -1,34 | 0,0407 | CLEC18A | 1,18 | 0,0412 |
| DHRS3 | -2,13 | 0,9139 | COL6A1 | 2,72 | 0,5545 | CLEC1A | -1,78 | 0,0219 |
| DHRS4-AS1 | -1,94 | 0,0194 | COL6A2 | 2,79 | 0,9327 | CLEC2B | -3,56 | 0,051 |
| DIO2 | 2,62 | 0,1081 | COL6A6 | -1,96 | 0,0107 | CLEC4E | 1,34 | 0,0281 |
| DIRAS3 | -18,18 | 0,1847 | COLEC12 | 3,45 | 0,3086 | CLHC1 | 2,01 | 0,0571 |
| DKK1 | -8,35 | 0,3146 | COLGALT2 | -1,65 | 0,0150 | CLSTN3 | -2,34 | 0,1096 |
| DLC1 | 2,03 | 0,3679 | COMMD4 | 2,37 | 0,1478 | CLU | -7,89 | 0,0167 |
| DLGAP1 | 4,67 | 0,1713 | COPG2 | 1,54 | 0,0392 | CMIP | -2,23 | 0,0866 |
| DLGAP5 | -2,18 | 0,7241 | COQ2 | 2,86 | 0,2229 | CNGB1 | 1,28 | 0,0417 |
| DLX1 | -2,39 | 0,6080 | COTL1 | 3,65 | 0,2523 | CNIH3 | 2,03 | 0,592 |
| DMAP1 | 2,64 | 0,1106 | COX7A1 | -2,22 | 0,8793 | CNN1 | 2,22 | 0,5835 |
| DMKN | -3,95 | 0,0448 | CPD | 2,09 | 0,0933 | CNOT6 | -2,22 | 0,0694 |
| DMRT3 | -1,2 | 0,0435 | CPE | -2,63 | 0,6986 | CNOT6L | -3,02 | 0,051 |
| DNAH14 | -3,97 | 0,2952 | CPED1 | 2,11 | 0,9686 | CNTROB | -1,67 | 0,0369 |
| DNAJB1 | 2,14 | 0,3881 | CPXM2 | 2,21 | 0,0732 | COA1 | 3,28 | 0,0646 |
| DNAJB11 | -1,41 | 0,0429 | CRACR2A | -1,87 | 0,0112 | COA7 | -2,05 | 0,0455 |
| DNAJB4 | 2,63 | 0,0133 | CREB3L1 | -2,08 | 0,4809 | COBLL1 | 2,49 | 0,3514 |
| DNAJB5 | 2,68 | 0,2390 | CREBZF | 1,45 | 0,0224 | COL4A1 | 4,14 | 0,0567 |
| DNAJB9 | 1,53 | 0,0188 | CRHR1-IT1 | 2,1 | 0,1823 | COL5A1 | -3,44 | 0,6553 |
| DNAJC15 | 2,44 | 0,7693 | CRISPLD2 | 1,81 | 0,0288 | COL6A3 | -2,01 | 0,3989 |
| DNAJC18 | -1,42 | 0,0279 | CROT | -1,76 | 0,0474 | COL9A1 | 1,38 | 0,0308 |
| DNAJC21 | -1,78 | 0,0166 | CRTC2 | 1,22 | 0,0413 | COLEC12 | -9,61 | 0,0115 |
| DNAJC30 | 1,64 | 0,0275 | CRYZ | -2,58 | 0,0020 | COMMD1 | 1,36 | 0,0397 |
| DNAJC6 | 1,88 | 0,0421 | CSF1 | 2,08 | 0,6319 | COPRS | 3,31 | 0,0098 |
| DNAJC7 | 1,6 | 0,0256 | CSF2RB | 2,71 | 0,5695 | COQ10B | 1,3 | 0,0094 |
| DNM1 | -1,9 | 0,0301 | CSNK1A1 | 1,46 | 0,0481 | COX17 | 2,2 | 0,0512 |
| DNPEP | -2,17 | 0,8992 | CST1 | -2,2 | 0,6684 | COX7A1 | 9,76 | 0,0921 |
| DOCK5 | 2,73 | 0,0975 | CST4 | -2,48 | 0,9640 | COX7A2L | 1,38 | 0,0464 |
| DPEP2 | 1,24 | 0,0291 | CSTA | -3,43 | 0,0352 | CPD | -2,12 | 0,2925 |
| DPH2 | -2,22 | 0,4248 | CTC-490E21.11 | 2,09 | 0,0177 | CPE | 2,06 | 0,9518 |
| DPPA3 | -1,77 | 0,0046 | CTDNEP1 | 2,53 | 0,1408 | CPM | 2,39 | 0,2394 |
| DPT | -14,4 | 0,0001 | CTH | -2,14 | 0,0379 | CPNE2 | -2,01 | 0,0119 |
| DPY19L1 | 2,63 | 0,1496 | CTNNA1 | 1,94 | 0,0159 | CPNE3 | 1,66 | 0,0499 |
| DPY19L4 | 2,71 | 0,0190 | CTPS2 | 1,78 | 0,0153 | CPXM2 | -2,01 | 0,0575 |
| DPYD | 2,54 | 0,6041 | CTR9 | 3 | 0,0960 | CREB1 | -2,05 | 0,193 |
| DPYSL3 | -8,71 | 0,4801 | CWC22 | 2,15 | 0,0456 | CRHBP | 34,91 | 0,2157 |
| DRAP1 | 2,3 | 0,4982 | CXCL16 | 2,57 | 0,0693 | CROT | 1,62 | 0,0436 |
| DSE | 2,44 | 0,1935 | CXCL6 | -1,88 | 0,0283 | CRYAB | 4,69 | 0,1469 |
| DSEL | 2,05 | 0,1090 | CXCL9 | -1,41 | 0,0118 | CRYZ | 1,84 | 0,0264 |
| DSP | -4,5 | 0,3798 | CXCR4 | 9,87 | 0,8960 | CSE1L | -2,39 | 0,2435 |
| DSPP | -1,31 | 0,0499 | CXorf56 | 2,03 | 0,0317 | CSRNP3 | 1,7 | 0,0248 |
| DTNA | 16,52 | 0,0986 | CXorf57 | -2,05 | 0,0259 | CST1 | 20,3 | 0,1884 |
| DTNB | -2,64 | 0,3860 | CYB561D2 | -1,18 | 0,0429 | CST2 | 2,43 | 0,2492 |
| DUS2 | -2,4 | 0,5612 | CYB5B | 2,79 | 0,1196 | CST4 | 7,54 | 0,1864 |
| DUS4L | -2,03 | 0,5411 | CYB5R1 | -2,41 | 0,6788 | CST9 | -1,21 | 0,0445 |
| DUSP10 | 3,06 | 0,0417 | CYP1B1 | -3,02 | 0,3458 | CTB-151G24.2 | 2,33 | 0,0245 |
| DUSP3 | -2,74 | 0,7970 | CYP2R1 | -3,09 | 0,1149 | CTBS | 1,74 | 0,0071 |
| DUSP4 | 2,79 | 0,9622 | CYP2S1 | 13,66 | 0,3091 | CTCF | -2,59 | 0,1848 |
| DUTP5 | -1,46 | 0,0151 | CYTH1 | 2,2 | 0,1543 | CTDSP2 | -2 | 0,03 |
| DUXAP8 | 11,06 | 0,0442 | CYTIP | -1,66 | 0,0099 | CTHRC1 | -2,16 | 0,9989 |
| DYNAP | 1,5 | 0,0293 | CYTL1 | -4,64 | 0,6493 | CTSS | 2,15 | 0,6339 |
| DYNC1I1 | 1,18 | 0,0464 | DAAM1 | -2,62 | 0,1987 | CUL4A | -1,59 | 0,0245 |
| DYNC2H1 | -2,0 | 0,6205 | DAB2 | 2,32 | 0,0585 | CWF19L1 | -2,02 | 0,0399 |
| DYSF | 3,56 | 0,8562 | DACH1 | -4,25 | 0,7822 | CXCL8 | 2,38 | 0,28 |
| E2F5 | -2,26 | 0,1466 | DAGLB | 2,04 | 0,5229 | CXCR4 | -9,03 | 0,2567 |
| E2F8 | -2,24 | 0,3564 | DAW1 | 3,1 | 0,6774 | CXorf56 | -2,8 | 0,0391 |
| EBF1 | 4,05 | 0,3055 | DBF4 | -2,2 | 0,6847 | CXorf57 | 2,42 | 0,0447 |
| EBF2 | 3,47 | 0,2653 | DBR1 | 2,49 | 0,3401 | CYB5B | -2,03 | 0,1197 |
| EBF3 | 1,92 | 0,0378 | DCAF4 | -1,76 | 0,0284 | CYB5D1 | 3,16 | 0,2641 |
| EDN1 | 2,11 | 0,6531 | DCN | 2,55 | 0,6402 | CYB5R1 | 2,84 | 0,3358 |
| EEF1D | -2,19 | 0,6232 | DCTPP1 | 2,82 | 0,3253 | CYBRD1 | 1,59 | 0,0302 |
| EEF1E1P1 | -1,38 | 0,0312 | DCUN1D5 | -1,43 | 0,0353 | CYP1A1 | 1,4 | 0,0285 |
| EEFSEC | -2,1 | 0,6449 | DDRGK1 | 2,02 | 0,1959 | CYP2S1 | -3,57 | 0,7022 |
| EFNB2 | -5,14 | 0,5341 | DDX11 | 2,34 | 0,2055 | CYP4F12 | -1,44 | 0,0098 |
| EGR1 | -2,67 | 0,4865 | DDX39A | 2,31 | 0,1512 | CYTH1 | -2,78 | 0,0378 |
| EHD3 | -2,03 | 0,8801 | DDX3X | 1,44 | 0,0152 | DAAM2 | 2,6 | 0,1225 |
| EIF1AX | -2,77 | 0,3301 | DDX51 | 1,4 | 0,0455 | DAPK1 | -1,83 | 0,0255 |
| EIF1B | -1,9 | 0,0168 | DEDD | 2,08 | 0,1925 | DARS-AS1 | 2,17 | 0,081 |
| EIF2B3 | 1,46 | 0,0405 | DEFB107A | 1,35 | 0,0403 | DCAF4 | 1,78 | 0,0471 |
| EIF2B5 | 1,8 | 0,0290 | DENND1A | 2,23 | 0,2249 | DCAF7 | -1,66 | 0,0187 |
| EIF2S3 | -2,21 | 0,8300 | DENND6A | 1,81 | 0,0274 | DCBLD2 | 2,76 | 0,1844 |
| EIF3A | -2,01 | 0,2568 | DEPDC1B | 2,86 | 0,1089 | DCLRE1A | -2,45 | 0,0022 |
| EIF4EBP1 | -3,86 | 0,6343 | DES | 1,68 | 0,0021 | DCPS | -2,33 | 0,0402 |
| EIF5A2 | -1,52 | 0,0424 | DFNA5 | -2,4 | 0,8203 | DCTD | 2,4 | 0,0761 |
| ELFN1 | -1,62 | 0,0335 | DHCR24 | 3,13 | 0,0893 | DCTN6 | 1,76 | 0,0495 |
| ELMOD1 | -3,07 | 0,0431 | DHRS9 | -2,3 | 0,2951 | DCTPP1 | -2,24 | 0,186 |
| ELOVL2 | -2,11 | 0,1937 | DHX36 | -1,38 | 0,0231 | DDAH1 | 2 | 0,1028 |
| ELOVL6 | 2,4 | 0,2971 | DIAPH2 | -1,79 | 0,0374 | DDIAS | -2,03 | 0,2926 |
| ELP5 | -3,67 | 0,8143 | DIO2 | -2,54 | 0,0135 | DDIT4 | -2,94 | 0,3108 |
| ELSPBP1 | 1,22 | 0,0333 | DIRAS3 | -30,98 | 0,1609 | DDIT4L | -1,65 | 0,0288 |
| EMCN | 3,82 | 0,9895 | DIRC3 | -1,4 | 0,0267 | DDRGK1 | -1,18 | 0,0483 |
| EMD | -2,21 | 0,7736 | DKK1 | -4,04 | 0,4091 | DDX11 | -2,34 | 0,0153 |
| EML4 | 2,76 | 0,0372 | DKK3 | -3,45 | 0,1794 | DDX23 | -1,71 | 0,0134 |
| EMP1 | -2,85 | 0,8177 | DLC1 | 2,03 | 0,1907 | DDX39A | -2,01 | 0,1877 |
| EMP3 | 2,32 | 0,2888 | DLGAP1 | -3,73 | 0,0132 | DDX3Y | -2,19 | 0,943 |
| ENAH | 2,13 | 0,0194 | DLGAP5 | 2,81 | 0,6267 | DEFB112 | 1,27 | 0,0268 |
| ENPP4 | 2,19 | 0,0500 | DLX1 | -3,32 | 0,1826 | DEFB128 | 1,52 | 0,0409 |
| ENTPD7 | -2,07 | 0,9937 | DMAP1 | 3,74 | 0,0279 | DENND6A | -1,71 | 0,0038 |
| EPAS1 | 8,54 | 0,2389 | DMKN | -5,6 | 0,6135 | DEPDC1 | -3,65 | 0,7339 |
| EPHA2 | -2,15 | 0,6849 | DNAH14 | -2,66 | 0,2451 | DEPDC5 | -1,5 | 0,0249 |
| EPHA5 | 3,02 | 0,0549 | DNAH7 | -1,4 | 0,0417 | DEPTOR | 2,85 | 0,2314 |
| EPHB2 | -7,85 | 0,3319 | DNAJB11 | -1,83 | 0,0312 | DERA | -2,04 | 0,1491 |
| EPPIN | -1,44 | 0,0491 | DNAJB5 | 3,05 | 0,1218 | DERL2 | 1,53 | 0,0085 |
| ERAP1 | -2,13 | 0,6072 | DNAJC21 | -2,45 | 0,0060 | DGKQ | -1,41 | 0,037 |
| ERBB4 | -3,41 | 0,3074 | DNAJC28 | 3,69 | 0,1123 | DHRS4-AS1 | -1,94 | 0,0071 |
| ERCC2 | -2,19 | 0,7543 | DNAJC7 | 2,18 | 0,0578 | DHX30 | -2,06 | 0,583 |
| ERG | 4,25 | 0,9654 | DNAJC9 | 3,7 | 0,4699 | DHX35 | -1,7 | 0,005 |
| ERLIN1 | 2,2 | 0,1939 | DNM1L | -2 | 0,0020 | DIDO1 | -1,85 | 0,0259 |
| ERLIN2 | 2,05 | 0,0244 | DOCK8 | -1,4 | 0,0413 | DIO2 | 3,61 | 0,0596 |
| ERMAP | 2,21 | 0,0966 | DOK4 | -2,08 | 0,8583 | DIP2A | -2,98 | 0,0961 |
| ERRFI1 | -2,77 | 0,0249 | DPH3 | -2,16 | 0,2467 | DIXDC1 | 2,28 | 0,1006 |
| ERV3-1 | 3,24 | 0,5361 | DPT | -2,23 | 0,1284 | DKK3 | 4,7 | 0,3524 |
| ESCO1 | 3,14 | 0,1709 | DPY19L4 | 2,38 | 0,0196 | DLGAP1 | 17,02 | 0,0052 |
| ESCO2 | -2,59 | 0,0930 | DPYD | 2,08 | 0,6633 | DLGAP5 | -6,23 | 0,3496 |
| ESM1 | 3,78 | 0,9353 | DPYSL3 | -2,41 | 0,8515 | DNAJB4 | 2,25 | 0,1487 |
| ETNK1 | 1,42 | 0,0261 | DR1 | -1,49 | 0,0362 | DNAJB9 | 1,32 | 0,0253 |
| ETV1 | -2,44 | 0,2981 | DRAM1 | -3,06 | 0,0550 | DNAJC15 | 3,51 | 0,8104 |
| EXOC3 | 1,37 | 0,0381 | DSN1 | 7,93 | 0,1493 | DNAJC17 | -1,53 | 0,0184 |
| EXOG | 1,61 | 0,0496 | DSP | -7,02 | 0,2441 | DNAJC21 | 1,31 | 0,0444 |
| EXOSC8 | -2,46 | 0,3711 | DTL | 2,65 | 0,5238 | DNAJC30 | 1,96 | 0,0115 |
| EZH2 | -4,38 | 0,0807 | DTYMK | 5,23 | 0,0953 | DNAJC6 | 1,85 | 0,0235 |
| F13B | -1,39 | 0,0259 | DUS4L | -2,3 | 0,1296 | DNAJC9 | -4,01 | 0,0729 |
| F2RL2 | 5,19 | 0,0597 | DUSP1 | 3,33 | 0,3374 | DNALI1 | 1,47 | 0,0126 |
| F3 | 7,31 | 0,0558 | DUSP4 | 4,94 | 0,7850 | DNM1L | 1,27 | 0,024 |
| FABP4 | 3,17 | 0,1512 | DYNAP | 1,68 | 0,0133 | DNMT3A | -2,83 | 0,2306 |
| FADS1 | 2,59 | 0,2572 | DYNC2H1 | -2,32 | 0,2507 | DNTTIP1 | -1,59 | 0,0135 |
| FADS2 | 3,18 | 0,1839 | DYNC2LI1 | -1,7 | 0,0344 | DOK1 | 2,04 | 0,1374 |
| FAM101B | -1,55 | 0,0477 | DYSF | 2,2 | 0,4882 | DPH2 | -1,67 | 0,0108 |
| FAM111A | -2,14 | 0,7847 | DZIP1 | -2,71 | 0,0153 | DPM3 | 3,16 | 0,3837 |
| FAM122A | -1,29 | 0,0275 | E2F5 | -2,82 | 0,1849 | DPT | -4,93 | 0,1565 |
| FAM126B | 2,02 | 0,1499 | E2F8 | 2,2 | 0,7381 | DPYSL3 | -3,99 | 0,3218 |
| FAM132B | 1,25 | 0,0484 | EBF2 | -2,4 | 0,5684 | DRAM1 | 3,73 | 0,0786 |
| FAM156A | 2,48 | 0,6807 | EBF3 | 1,48 | 0,0368 | DSN1 | -8,58 | 0,0811 |
| FAM171A1 | 1,32 | 0,0243 | EBNA1BP2 | 2,84 | 0,1910 | DST | 1,57 | 0,0039 |
| FAM172BP | -1,51 | 0,0330 | EBP | 2,51 | 0,1645 | DSTN | 1,97 | 0,031 |
| FAM180A | 4,79 | 0,9339 | ECT2 | 3,37 | 0,1812 | DTL | -3,7 | 0,3377 |
| FAM198B | 2,17 | 0,2918 | EDIL3 | -18,7 | 0,1166 | DTNA | 10,4 | 0,2132 |
| FAM217B | 1,6 | 0,0052 | EDN1 | 4,34 | 0,3408 | DTYMK | -2,45 | 0,1212 |
| FAM222A | 1,3 | 0,0247 | EDNRA | -3,09 | 0,4038 | DUS2 | -2,04 | 0,1729 |
| FAM27E3 | -2,67 | 0,2186 | EFCC1 | -1,3 | 0,0341 | DUSP1 | -2,35 | 0,2782 |
| FAM46A | -1,66 | 0,0105 | EFEMP1 | 6,05 | 0,0374 | DUSP10 | 2,06 | 0,1877 |
| FAM73B | -1,2 | 0,0395 | EFEMP2 | -2,14 | 0,7136 | DUSP5 | -2,38 | 0,2391 |
| FAM83D | -2,11 | 0,9087 | EFNA5 | 2,91 | 0,1039 | DYNC1I1 | 1,25 | 0,0245 |
| FANCF | -1,21 | 0,0400 | EGF | -1,5 | 0,0113 | DYNLT3 | 2,04 | 0,0026 |
| FANK1 | 2,32 | 0,1231 | EGFLAM | -4,59 | 0,1497 | DYRK2 | 1,41 | 0,0438 |
| FARP1 | 7,91 | 0,0023 | EGR2 | 2 | 0,3635 | E2F8 | -11,89 | 0,1979 |
| FAS | 2,03 | 0,0327 | EHD3 | -2,62 | 0,4502 | E4F1 | -1,5 | 0,0172 |
| FBL | -2,02 | 0,7032 | EHMT1 | 2,04 | 0,1418 | EBF1 | 6,27 | 0,2225 |
| FBLN2 | -4,91 | 0,3148 | EIF1AX | -2,71 | 0,4490 | EBF2 | 6,53 | 0,296 |
| FBXL21 | -2,7 | 0,0262 | EIF1AY | 2,25 | 0,7790 | EBLN2 | -1,83 | 0,0434 |
| FBXL6 | -2,57 | 0,8986 | EIF1B | -2 | 0,0018 | EBNA1BP2 | -1,78 | 0,0062 |
| FBXO27 | 2,17 | 0,0400 | EIF3A | -1,88 | 0,0426 | EBP | -2,84 | 0,0888 |
| FBXO32 | 24,53 | 0,0217 | EIF3C | 2,53 | 0,1549 | ECHDC2 | 1,33 | 0,043 |
| FBXO5 | -2,13 | 0,4791 | ELAC2 | 2,62 | 0,4408 | ECT2 | -3,39 | 0,1308 |
| FCF1P10 | 1,48 | 0,0189 | ELF4 | -3,19 | 0,3495 | EDC4 | -1,76 | 0,0058 |
| FCF1P3 | 1,25 | 0,0446 | ELL2 | 3 | 0,6585 | EDIL3 | 15,81 | 0,0531 |
| FDFT1 | 2,95 | 0,0200 | ELMOD2 | -2,29 | 0,0260 | EDNRA | 5,28 | 0,1772 |
| FER | -2,16 | 0,0583 | ELOVL1 | 2,32 | 0,2754 | EEFSEC | -1,74 | 0,0031 |
| FERMT1 | -6,14 | 0,2098 | ELOVL6 | 2,38 | 0,6379 | EFEMP1 | -5,52 | 0,0151 |
| FGF1 | 2,41 | 0,0049 | ELP5 | -3,71 | 0,7256 | EFNB2 | -7 | 0,9148 |
| FGF12 | -1,6 | 0,0185 | EMB | -2,09 | 0,1132 | EFTUD2 | -2,67 | 0,015 |
| FGF13-AS1 | 1,57 | 0,0249 | EMC10 | 1,53 | 0,0479 | EGF | 1,64 | 0,0073 |
| FGF5 | 2,98 | 0,3637 | EMC9 | 2,63 | 0,0261 | EGFL6 | 1,28 | 0,0245 |
| FGL2 | -3,83 | 0,3546 | EMCN | 7,41 | 0,4212 | EGFL7 | -1,6 | 0,0176 |
| FHAD1 | 1,51 | 0,0359 | EMD | -2,92 | 0,6294 | EGFLAM | 4,45 | 0,0565 |
| FIG4 | 2,03 | 0,0163 | EMP1 | 2,21 | 0,3420 | EGR1 | -2,78 | 0,4823 |
| FKBP14 | 1,7 | 0,0420 | ENOSF1 | 2,25 | 0,0714 | EGR2 | -5,25 | 0,1224 |
| FKBP5 | 1,69 | 0,0157 | ENPP4 | 2,76 | 0,0016 | EHMT1 | -1,77 | 0,0409 |
| FKTN | -2,03 | 0,1171 | ENTHD2 | 1,27 | 0,0413 | EIF2AK2 | 1,55 | 0,0142 |
| FLI1 | -3,25 | 0,8959 | ENTPD4 | -2,63 | 0,9722 | EIF2B2 | -1,32 | 0,0208 |
| FLNB | -4,34 | 0,9901 | EOGT | 2,3 | 0,5611 | EIF2S3 | -3,74 | 0,1264 |
| FLRT2 | -6,23 | 0,0049 | EPAS1 | 3,67 | 0,3525 | EIF3C | -2,08 | 0,0528 |
| FLRT3 | -3,31 | 0,2047 | EPB41L3 | 12,52 | 0,2273 | EIF4EBP1 | -2,2 | 0,2685 |
| FLT1 | -5,34 | 0,0272 | EPDR1 | -2,88 | 0,5556 | ELAC2 | -3,16 | 0,1232 |
| FNBP1L | 2,04 | 0,3005 | EPGN | -3,86 | 0,0152 | ELAVL1 | -2,13 | 0,1206 |
| FNDC9 | -1,24 | 0,0336 | EPHA4 | 5,12 | 0,3145 | ELF2 | -1,33 | 0,014 |
| FOSL1 | 2,06 | 0,9234 | EPHA5 | 2,98 | 0,6066 | ELOVL1 | -2,94 | 0,0137 |
| FOXM1 | -2,09 | 0,7998 | EPHB2 | -3,41 | 0,9200 | EMCN | -2,12 | 0,2989 |
| FOXN4 | 1,22 | 0,0477 | EPS15 | 3,21 | 0,4246 | EMILIN1 | 2,43 | 0,7662 |
| FRMPD4 | 3,32 | 0,0635 | EPSTI1 | -2,27 | 0,3089 | EML4 | 2,03 | 0,0383 |
| FRRS1 | 2,11 | 0,0967 | ERBB4 | -4,33 | 0,6877 | EMP1 | -6,84 | 0,0119 |
| FSCN1 | -3,19 | 0,7886 | ERCC5 | -3,22 | 0,3442 | ENAH | 2,64 | 0,068 |
| FST | 2,52 | 0,1122 | ERCC8 | -1,69 | 0,0159 | ENC1 | -2,45 | 0,4483 |
| FUT10 | 2,49 | 0,2576 | ERG | 2,57 | 0,3946 | ENOSF1 | -1,46 | 0,0341 |
| FXYD5 | -1,41 | 0,0358 | ERGIC3 | -2,54 | 0,8124 | ENOX1 | -1,27 | 0,0221 |
| FYN | -2,0 | 0,3939 | ERICH6B | -1,32 | 0,0254 | EOGT | -2,56 | 0,564 |
| GABPB2 | 2,17 | 0,2244 | ERLEC1 | -1,61 | 0,0192 | EPAS1 | 2,35 | 0,7931 |
| GADD45A | -2,14 | 0,6667 | ERLIN1 | 2,23 | 0,1390 | EPB41L3 | -15,96 | 0,0191 |
| GALC | 1,36 | 0,0415 | ERRFI1 | -1,82 | 0,0342 | EPDR1 | 2,74 | 0,0818 |
| GALK2 | 2,71 | 0,0435 | ESAM | 2,86 | 0,4218 | EPGN | 3,95 | 0,0128 |
| GALNT5 | 12,75 | 0,0158 | ESM1 | 3,51 | 0,8711 | EPHA2 | -2 | 0,2147 |
| GAPDHP36 | 1,48 | 0,0231 | ETHE1 | 2,38 | 0,2796 | EPHA4 | -2,86 | 0,2312 |
| GAS8-AS1 | 1,23 | 0,0439 | ETV1 | -3,57 | 0,0808 | EPHB2 | -2,6 | 0,236 |
| GATA6-AS1 | -2,28 | 0,4053 | ETV6 | 4,31 | 0,1545 | EPS15 | -3,54 | 0,1935 |
| GATC | 2,82 | 0,1339 | EWSR1 | 2,28 | 0,0255 | ERAP1 | -3,09 | 0,0393 |
| GBP4 | -2,72 | 0,1390 | EXO1 | 2,31 | 0,4664 | ERCC2 | -3,4 | 0,119 |
| GCOM1 | -1,83 | 0,0171 | EXOC3-AS1 | 1,6 | 0,0176 | ERCC5 | 2,79 | 0,0472 |
| GDPGP1 | -2,29 | 0,4649 | EXOSC5 | 1,85 | 0,0239 | ERCC6L2 | 1,78 | 0,0121 |
| GFPT2 | -3,14 | 0,3690 | EYA3 | 1,56 | 0,0418 | ERGIC2 | 1,86 | 0,0082 |
| GGT5 | 3,58 | 0,1955 | EZR | 4,17 | 0,5620 | ERI1 | -2,03 | 0,1195 |
| GINS1 | -2,26 | 0,7995 | F3 | 2,46 | 0,9737 | ERLEC1 | 2,08 | 0,0008 |
| GIPC2 | 2,12 | 0,0747 | FABP5 | -2,34 | 0,1305 | ERLIN2 | 1,65 | 0,015 |
| GIT1 | -2,04 | 0,6799 | FADS1 | 2,86 | 0,9171 | ERMAP | 2,57 | 0,021 |
| GJA5 | -2,15 | 0,2801 | FADS2 | 12,02 | 0,1931 | ERV3-1 | 2,08 | 0,8806 |
| GK | -2,84 | 0,1305 | FAM103A1 | -2,08 | 0,0959 | ESCO1 | 3,73 | 0,0767 |
| GK5 | 1,38 | 0,0281 | FAM107B | -2,21 | 0,7570 | ESCO2 | -2,15 | 0,1382 |
| GLCE | -1,91 | 0,0041 | FAM111B | -2,37 | 0,9518 | ESYT1 | -2,52 | 0,055 |
| GLIPR2 | 2,11 | 0,5437 | FAM114A1 | -2,46 | 0,1120 | ETHE1 | -1,68 | 0,0271 |
| GLIS3 | 2,53 | 0,9781 | FAM126B | 2,76 | 0,0021 | ETNK2 | -2,57 | 0,2109 |
| GMNN | -4,46 | 0,2363 | FAM129B | -2,15 | 0,5668 | ETV6 | -2,19 | 0,276 |
| GMPR | 2,33 | 0,0941 | FAM149B1 | 1,7 | 0,0248 | EXOSC5 | -1,45 | 0,0234 |
| GNAI2P2 | 1,2 | 0,0283 | FAM151B | -2,27 | 0,0459 | EZH2 | -6,27 | 0,0676 |
| GNG11 | 2,79 | 0,2843 | FAM155A | 1,54 | 0,0250 | EZR | -3,1 | 0,1018 |
| GNG2 | 2,45 | 0,4837 | FAM156A | 2,59 | 0,4077 | F2RL2 | 4,58 | 0,1444 |
| GNPTAB | 2,44 | 0,1447 | FAM157B | 1,44 | 0,0383 | F3 | 2,15 | 0,123 |
| GOLGA8T | 1,29 | 0,0458 | FAM174A | -2,03 | 0,0277 | F5 | -1,17 | 0,0375 |
| GOT1 | -2,88 | 0,5137 | FAM174B | -2,04 | 0,0146 | FABP7 | 1,22 | 0,0441 |
| GPAM | -2,0 | 0,7254 | FAM177A1 | -1,64 | 0,0206 | FADS2 | -3,75 | 0,5698 |
| GPANK1 | -2,43 | 0,7467 | FAM195A | -2,4 | 0,8610 | FAM101B | -1,4 | 0,0336 |
| GPAT2 | -2,08 | 0,8685 | FAM196B | 2,93 | 0,0079 | FAM111A | -2,61 | 0,6347 |
| GPC6 | -2,69 | 0,1152 | FAM198B | 2,26 | 0,1314 | FAM117B | 2,66 | 0,0073 |
| GPCPD1 | -2,3 | 0,0273 | FAM200A | -2,34 | 0,2459 | FAM13B | 2,53 | 0,2045 |
| GPNMB | -2,48 | 0,9320 | FAM201B | 1,34 | 0,0263 | FAM151B | 1,51 | 0,0434 |
| GPR137B | 2,71 | 0,0523 | FAM205A | -1,28 | 0,0326 | FAM171B | 2,54 | 0,2558 |
| GPR137C | 1,62 | 0,0257 | FAM206A | 3 | 0,3453 | FAM174A | 3,37 | 0,0976 |
| GPR148 | -1,63 | 0,0095 | FAM20B | 2,07 | 0,5032 | FAM180A | 5,91 | 0,5075 |
| GPR157 | -2,52 | 0,3345 | FAM213B | 1,66 | 0,0499 | FAM180B | 1,43 | 0,03 |
| GPR161 | -2,33 | 0,8039 | FAM216A | 2,36 | 0,3214 | FAM182B | 1,35 | 0,0471 |
| GPR89B | -1,76 | 0,0136 | FAM229B | -3,18 | 0,0097 | FAM195A | 2,18 | 0,1621 |
| GPRC5A | 6,76 | 0,2763 | FAM231A | -1,36 | 0,0169 | FAM196B | -3,74 | 0,0436 |
| GPT2 | -3,8 | 0,4915 | FAM231C | -1,27 | 0,0170 | FAM200B | 2,46 | 0,0192 |
| GPX3 | 2,43 | 0,0011 | FAM24B | 2,21 | 0,8628 | FAM208A | 1,95 | 0,0439 |
| GPX4 | 2,33 | 0,2110 | FAM50B | -1,36 | 0,0070 | FAM223A | 1,58 | 0,0307 |
| GRAMD1B | 3,34 | 0,0641 | FAM60A | 2,86 | 0,3302 | FAM229B | 2,44 | 0,0074 |
| GRAMD3 | 2,59 | 0,0612 | FAM63B | -2,02 | 0,0069 | FAM231A | 1,43 | 0,0047 |
| GRB10 | -3,3 | 0,9965 | FAM66A | -2,15 | 0,3239 | FAM231C | 1,42 | 0,0072 |
| GRB14 | -3,43 | 0,1433 | FAM72A | 4,16 | 0,4514 | FAM231D | 2,5 | 0,0191 |
| GREM1 | 6,79 | 0,0097 | FAM72B | 2,8 | 0,7343 | FAM27E2 | -1,69 | 0,0228 |
| GRIA1 | 1,64 | 0,0425 | FAM72C | 2,7 | 0,3052 | FAM3C | 1,95 | 0,0402 |
| GRIA3 | -2,4 | 0,1904 | FAM72D | 3,65 | 0,3523 | FAM41C | 1,34 | 0,0093 |
| GRIK2 | -2,1 | 0,3066 | FAM76A | 1,49 | 0,0304 | FAM50B | 1,63 | 0,0272 |
| GRM8 | 1,47 | 0,0151 | FAM83D | 2,03 | 0,4363 | FAM53C | -2,04 | 0,0167 |
| GSE1 | -2,1 | 0,0859 | FAM91A1 | 2,63 | 0,0549 | FAM65A | -2,05 | 0,1515 |
| GSN | 5,79 | 0,2427 | FANCC | 2,69 | 0,4424 | FAM65B | 1,24 | 0,0115 |
| GTDC1 | 2,74 | 0,0808 | FANCI | 2,9 | 0,1574 | FAM69A | 2,05 | 0,7329 |
| GTF2H1 | 3,83 | 0,0286 | FARP1 | 5,9 | 0,0266 | FAM72A | -4,23 | 0,1453 |
| GTPBP2 | -2,87 | 0,4732 | FAT1 | -3,53 | 0,5775 | FAM72B | -3,39 | 0,3706 |
| GUCD1 | 2,03 | 0,2228 | FBLN2 | -4,48 | 0,5828 | FAM72C | -3,78 | 0,068 |
| GUCY1B3 | -2,32 | 0,0239 | FBLN5 | 3,38 | 0,6690 | FAM72D | -4,13 | 0,1307 |
| GVINP1 | -1,5 | 0,0105 | FBXL17 | -1,76 | 0,0120 | FAM83D | -5,75 | 0,2844 |
| GXYLT2 | 2,57 | 0,7208 | FBXL3 | 2,4 | 0,1162 | FAM91A1 | -2,04 | 0,0904 |
| H19 | -2,65 | 0,1994 | FBXO38 | 2,19 | 0,1146 | FANCA | -2,45 | 0,1596 |
| H2AFJ | 2,17 | 0,0649 | FBXO40 | -1,25 | 0,0282 | FANCC | -2,02 | 0,1541 |
| H2AFZ | -3,59 | 0,3697 | FBXO8 | -1,94 | 0,0326 | FANCD2 | -2,84 | 0,3365 |
| H2BFM | -1,59 | 0,0317 | FBXW11 | -2,53 | 0,1700 | FANCI | -2,28 | 0,1032 |
| H3F3C | -2,01 | 0,2428 | FCER1G | 1,85 | 0,0103 | FAR1 | -1,21 | 0,0386 |
| H6PD | -2,37 | 0,5897 | FCGRT | -2,25 | 0,7379 | FAT1 | 3,56 | 0,1091 |
| HACD3 | -2,13 | 0,0496 | FDPS | 2,68 | 0,1052 | FBF1 | -1,2 | 0,0191 |
| HAPLN1 | 6,42 | 0,1045 | FEN1 | 3,32 | 0,1260 | FBL | -2,7 | 0,0192 |
| HAS2 | -2,9 | 0,5539 | FERMT1 | -6,12 | 0,4518 | FBLN1 | -2,54 | 0,3112 |
| HAS3 | 2,05 | 0,1282 | FGF13-AS1 | 1,53 | 0,0289 | FBN1 | -2 | 0,3995 |
| HAT1 | -2,81 | 0,2260 | FGF5 | -2 | 0,2215 | FBXL3 | -2,78 | 0,093 |
| HAUS6 | -2,4 | 0,4569 | FGF7 | 2,5 | 0,6421 | FBXO24 | -1,15 | 0,0493 |
| HAVCR1 | -1,36 | 0,0442 | FGFR1OP | -1,31 | 0,0292 | FBXO32 | 16,62 | 0,0363 |
| HBP1 | 3,32 | 0,0097 | FGGY | -2,04 | 0,2666 | FBXO43 | -3,38 | 0,3992 |
| HCG27 | -1,4 | 0,0249 | FGL2 | -5,94 | 0,0799 | FBXO5 | -2,82 | 0,4692 |
| HCN3 | -1,29 | 0,0130 | FHAD1 | 1,68 | 0,0266 | FEN1 | -3,79 | 0,0353 |
| HCRTR2 | -1,4 | 0,0121 | FIBIN | -2,32 | 0,2998 | FER | -3,08 | 0,2806 |
| HEATR4 | -1,24 | 0,0472 | FIGN | 4,49 | 0,0126 | FEZ2 | 2,1 | 0,4881 |
| HEATR5B | 2,16 | 0,5512 | FIGNL1 | 2,11 | 0,1933 | FGD4 | 2,65 | 0,2486 |
| HECTD4 | 2,91 | 0,0735 | FKBP11 | -2,37 | 0,1124 | FGF1 | 2,26 | 0,0057 |
| HEG1 | -4,28 | 0,9698 | FKBP7 | -3,18 | 0,0564 | FGF5 | 5,44 | 0,057 |
| HERPUD1 | -2,46 | 0,3633 | FKTN | -2,37 | 0,2173 | FGF7 | -2,25 | 0,5689 |
| HERPUD2 | -1,99 | 0,0094 | FLCN | 2,76 | 0,1313 | FGFR2 | -1,37 | 0,0368 |
| HEY2 | -3,94 | 0,0839 | FLII | 2,11 | 0,1242 | FGGY | 2,5 | 0,0402 |
| HFE | 1,31 | 0,0279 | FLRT3 | -2,42 | 0,6585 | FHL1 | 2,38 | 0,1094 |
| HGF | 2,4 | 0,9748 | FMN1 | 1 | 0,0220 | FIBIN | 2,04 | 0,4321 |
| HIBADH | 1,77 | 0,0276 | FNBP1L | 3,1 | 0,2924 | FIGN | -1,89 | 0,0442 |
| HIST1H1B | -2,54 | 0,3759 | FNIP2 | 2,15 | 0,6264 | FIGNL1 | -2,95 | 0,2077 |
| HIST1H1E | -2,51 | 0,0461 | FOSL1 | 2,83 | 0,4741 | FIP1L1 | 3,35 | 0,7118 |
| HIST1H2AC | 2,04 | 0,7954 | FOXC2-AS1 | -2,76 | 0,0070 | FKBP11 | 2,1 | 0,0373 |
| HIST1H2AG | 2,02 | 0,6654 | FOXM1 | 4,49 | 0,3777 | FKBP14 | 1,54 | 0,0054 |
| HIST1H2BH | -2,79 | 0,4531 | FOXN3 | -2,53 | 0,1950 | FLCN | -2 | 0,3376 |
| HIST1H3I | -2,04 | 0,2199 | FOXR1 | -1,34 | 0,0376 | FLI1 | -3,87 | 0,036 |
| HIST1H4C | -3,73 | 0,2196 | FRMD3 | 10,49 | 0,0313 | FLNB | -2,25 | 0,125 |
| HIST1H4E | -3,57 | 0,8142 | FRMPD4 | 13,73 | 0,0093 | FLRT2 | -3,81 | 0,0029 |
| HIST1H4I | -2,01 | 0,4788 | FRRS1L | -1,53 | 0,0339 | FLRT3 | -1,98 | 0,0274 |
| HIST2H2AA3 | 2,56 | 0,2634 | FRYL | 2,19 | 0,1525 | FLT1 | -3,8 | 0,0686 |
| HIST2H2AA4 | 3,32 | 0,2789 | FURIN | -2 | 0,5705 | FOPNL | 2,37 | 0,0408 |
| HIVEP1 | -2,39 | 0,3014 | FUS | 2,51 | 0,0878 | FOXC2-AS1 | 4,07 | 0,0138 |
| HIVEP2 | 1,38 | 0,0366 | GAB3 | 1,97 | 0,0123 | FOXM1 | -8,51 | 0,23 |
| HJURP | 2,27 | 0,7605 | GABBR2 | 25,41 | 0,1172 | FOXP2 | -1,24 | 0,015 |
| HLA-C | -2,94 | 0,9749 | GABPB1 | 2,09 | 0,0045 | FRAS1 | -1,39 | 0,0127 |
| HLA-DMA | 2,41 | 0,2447 | GABPB2 | 2,07 | 0,2621 | FRMD3 | -10,53 | 0,0044 |
| HMBS | 1 | 0,0369 | GABRG3 | 1,28 | 0,0243 | FRMPD4 | -2,26 | 0,5946 |
| HMGB1 | -2,06 | 0,6216 | GADD45A | -2,2 | 0,8949 | FRYL | -1,88 | 0,001 |
| HMGB2 | -2,79 | 0,2544 | GALE | 2,02 | 0,4567 | FSCN1 | -3,53 | 0,0961 |
| HMGN1P10 | -1,31 | 0,0194 | GALNT5 | -2,66 | 0,3270 | FSD1 | -1,26 | 0,0355 |
| HMGN1P13 | -1,61 | 0,0037 | GALT | 2,29 | 0,0718 | FSD1L | -2,1 | 0,1147 |
| HMGN1P30 | -1,44 | 0,0063 | GAP43 | -2,41 | 0,0215 | FUS | -1,81 | 0,0399 |
| HMGN1P38 | -1,57 | 0,0256 | GARNL3 | 2,02 | 0,2929 | FXYD5 | -1,83 | 0,0462 |
| HMGXB4 | -2,05 | 0,3063 | GART | 2,71 | 0,1163 | FYN | -2,22 | 0,4587 |
| HN1 | -2,21 | 0,6142 | GAS2L3 | 3,88 | 0,0625 | GABARAPL2 | 1,4 | 0,0313 |
| HNRNPA0 | -2,57 | 0,7485 | GAS5 | 2,14 | 0,4609 | GABBR2 | -22,12 | 0,0489 |
| HNRNPA3P16 | -1,31 | 0,0181 | GAS6 | -4,95 | 0,8142 | GABPB1 | -1,36 | 0,0447 |
| HNRNPDP2 | -1,6 | 0,0208 | GATA3 | -1,46 | 0,0303 | GABRG1 | 1,18 | 0,0369 |
| HNRNPH3 | -2,23 | 0,5007 | GATC | 2,59 | 0,0918 | GABRG3 | -1,25 | 0,0193 |
| HPS3 | 1,34 | 0,0152 | GBP5 | -2,44 | 0,1860 | GABRQ | 1,36 | 0,0409 |
| HRASLS | -2,05 | 0,1340 | GCKR | 2,4 | 0,8892 | GALNS | -2,01 | 0,0795 |
| HRCT1 | -2,7 | 0,1647 | GCNT1 | 2,27 | 0,8180 | GALNT5 | 28,03 | 0,0083 |
| HSD17B2 | 1,56 | 0,0178 | GCSH | -2,01 | 0,0261 | GAS2L3 | -4,29 | 0,1376 |
| HSD17B7 | 1,48 | 0,0401 | GDAP2 | 2,36 | 0,1383 | GAS6 | 4,61 | 0,4937 |
| HSPA4L | 2,48 | 0,0445 | GDPD3 | 2,13 | 0,8342 | GAS6-AS1 | 1,24 | 0,0434 |
| HSPB6 | 2,28 | 0,2445 | GFM2 | 2,02 | 0,2117 | GATAD2A | -2 | 0,0315 |
| HSPE1 | -2,07 | 0,3551 | GFPT2 | -2,21 | 0,7126 | GBA2 | -2,23 | 0,0816 |
| HSPG2 | -2,21 | 0,7328 | GGCX | 2,13 | 0,2604 | GBA3 | -1,36 | 0,0165 |
| HTR2A | -2,47 | 0,4544 | GGT1 | 1,62 | 0,0350 | GBP2 | -2,1 | 0,1617 |
| HYKK | -1,45 | 0,0113 | GGT5 | 2,63 | 0,5107 | GBP4 | -2,63 | 0,2846 |
| HYOU1 | -2,55 | 0,9156 | GHR | -2,36 | 0,0617 | GBP6 | -1,3 | 0,0412 |
| ID1 | -2,07 | 0,7168 | GIN1 | -2,13 | 0,1435 | GCKR | -2,03 | 0,8694 |
| ID2 | -2,03 | 0,1511 | GINS2 | 3,7 | 0,3124 | GCLM | -2,68 | 0,5304 |
| ID3 | -2,1 | 0,5649 | GINS4 | 3,82 | 0,2573 | GDE1 | 1,77 | 0,009 |
| IDI1 | 2,67 | 0,0125 | GJC1 | -4,6 | 0,4331 | GDNF | 1,31 | 0,048 |
| IER2 | -2,79 | 0,6406 | GK | -2,12 | 0,8248 | GGA3 | -1,87 | 0,0078 |
| IFI16 | -2,16 | 0,0260 | GLB1L | 2,19 | 0,1549 | GHDC | 1,36 | 0,044 |
| IFI27 | -4,14 | 0,0815 | GLTP | -2,19 | 0,9002 | GIMD1 | 1,37 | 0,0205 |
| IFI6 | -2,02 | 0,3018 | GMCL1 | -1,53 | 0,0137 | GINS1 | -3,56 | 0,1128 |
| IFNA10 | -1,44 | 0,0390 | GNA14 | 6,4 | 0,1479 | GINS2 | -4,32 | 0,072 |
| IFNA2 | -1,41 | 0,0221 | GNG11 | 2,53 | 0,6532 | GINS4 | -4,53 | 0,2469 |
| IFNAR2 | -2,21 | 0,3892 | GNL2 | -1,96 | 0,0146 | GJC1 | 6,87 | 0,3275 |
| IFNL3 | 1,28 | 0,0283 | GOLIM4 | -1,78 | 0,0354 | GK | -1,54 | 0,0376 |
| IFT88 | -2,31 | 0,1213 | GORASP1 | 2,09 | 0,1866 | GLIPR1 | 3,69 | 0,0008 |
| IGFBP1 | -1,19 | 0,0486 | GPAM | -4,44 | 0,1812 | GLIS3 | 2,11 | 0,906 |
| IGFBP2 | 11,38 | 0,0585 | GPAT2 | -3,25 | 0,2250 | GLUL | -2 | 0,0371 |
| IGFBP4 | 4,16 | 0,1060 | GPATCH2 | 2,04 | 0,0299 | GMEB1 | -2,16 | 0,0438 |
| IGFBP5 | 8,55 | 0,5818 | GPATCH3 | 1,62 | 0,0174 | GMNN | -2,17 | 0,519 |
| IGHV3-32 | -1,5 | 0,0444 | GPC6 | 2,04 | 0,5500 | GMPPB | -1,71 | 0,0447 |
| IGHV3-72 | -1,19 | 0,0496 | GPD1L | -1,47 | 0,0319 | GNA14 | -5,09 | 0,5957 |
| IGHV3-79 | 1,35 | 0,0282 | GPR141 | 1,59 | 0,0355 | GNAI1 | 2,21 | 0,1716 |
| IGKV2-40 | 1 | 0,0312 | GPR155 | -1,59 | 0,0181 | GNAZ | -1,57 | 0,0257 |
| IGKV3-25 | 1,28 | 0,0331 | GPR157 | -2,37 | 0,2793 | GNPTAB | 1,52 | 0,0303 |
| IGLV3-30 | 1,45 | 0,0393 | GPR19 | 1,94 | 0,0013 | GOLGA2 | 2,47 | 0,5443 |
| IGSF3 | -1,34 | 0,0323 | GPR4 | 2,12 | 0,1162 | GOLPH3L | -2,07 | 0,0339 |
| IGSF5 | 1,34 | 0,0361 | GPR85 | 2,5 | 0,1492 | GOPC | 2,01 | 0,0313 |
| IL13RA2 | -15,28 | 0,0816 | GPR89B | -1,95 | 0,0313 | GOT1 | -2,58 | 0,005 |
| IL15 | 1,66 | 0,0178 | GPRC5A | 5,86 | 0,4081 | GPATCH2 | -1,75 | 0,0498 |
| IL17RD | -3,21 | 0,8044 | GPRIN3 | -1,74 | 0,0316 | GPATCH3 | -1,53 | 0,0407 |
| IL18R1 | -2,97 | 0,0003 | GPX8 | -2,19 | 0,0259 | GPC3 | 1,23 | 0,0393 |
| IL1B | 2,18 | 0,1691 | GREM1 | 12,06 | 0,0557 | GPC4 | 1,75 | 0,0281 |
| IL1RL1 | -103,7 | 0,0001 | GREM2 | -1,89 | 0,0215 | GPC6 | -6,91 | 0,8811 |
| IL32 | -2,31 | 0,8276 | GRIA3 | -2,17 | 0,1457 | GPCPD1 | -2,53 | 0,2561 |
| IL7 | -3,92 | 0,0427 | GRK4 | -1,96 | 0,0403 | GPD1L | 1,42 | 0,0186 |
| INAFM2 | 2,13 | 0,0173 | GSE1 | -2,08 | 0,0333 | GPER1 | -2,32 | 0,0014 |
| ING2 | -2,02 | 0,3003 | GSG2 | 2,3 | 0,8328 | GPNMB | -2,01 | 0,6481 |
| INHBA | 2,93 | 0,1445 | GSTM3 | -2,45 | 0,0511 | GPR1 | -2,13 | 0,798 |
| INSIG1 | 1,73 | 0,0097 | GSTM4 | -2,81 | 0,1534 | GPR107 | 2,06 | 0,3079 |
| IQCA1 | -1,86 | 0,0012 | GSTO1 | -3,28 | 0,0864 | GPR135 | 1,73 | 0,0386 |
| IRAK1BP1 | -1,78 | 0,0162 | GSTZ1 | -1,56 | 0,0357 | GPR161 | -2,1 | 0,0264 |
| IRAK4 | 2,49 | 0,1475 | GTDC1 | 2,01 | 0,3372 | GPR19 | -1,68 | 0,0032 |
| IRF5 | 1,3 | 0,0316 | GTF2F2 | -1,6 | 0,0246 | GPR3 | -2,51 | 0,1409 |
| IRS1 | 2,57 | 0,3825 | GTF2H1 | 2,88 | 0,3295 | GPX3 | 1,86 | 0,0342 |
| ISLR | -7,91 | 0,1766 | GTPBP10 | 2,22 | 0,8709 | GPX8 | 1,8 | 0,0128 |
| ISLR2 | -1,5 | 0,0076 | GTSE1 | 2,02 | 0,5062 | GRAMD1B | 2,73 | 0,6557 |
| ISOC2 | 2,18 | 0,4162 | GUCD1 | 2,18 | 0,0961 | GRAMD3 | 2,21 | 0,263 |
| ISYNA1 | 9,14 | 0,2812 | H19 | 2,03 | 0,5412 | GRB14 | -2,26 | 0,4129 |
| ITGA11 | -2,51 | 0,2692 | H2AFX | 4,92 | 0,2369 | GRID2 | 1,24 | 0,0303 |
| ITGA2 | -2,37 | 0,1573 | H2AFY | 1,51 | 0,0212 | GRK5 | -2,61 | 0,1306 |
| ITGA5 | -2,07 | 0,9488 | H2BFM | -1,65 | 0,0238 | GSN | 2,56 | 0,7808 |
| ITGA6 | -9,12 | 0,0247 | HACD3 | -1,81 | 0,0273 | GSTM4 | 2,97 | 0,3716 |
| ITGA8 | -4,0 | 0,2830 | HACL1 | -2,31 | 0,0099 | GSTO1 | 3,46 | 0,059 |
| ITGB8 | 7,75 | 0,1164 | HAPLN1 | -2,3 | 0,4001 | GSTO2 | 1,34 | 0,0273 |
| ITM2A | -2,59 | 0,0266 | HAS2 | -7,83 | 0,2135 | GTF2H1 | 1,32 | 0,0477 |
| ITM2C | 3,62 | 0,3202 | HAUS8 | 2,23 | 0,2303 | GTF2H4 | -1,8 | 0,0125 |
| ITPR1 | 2,49 | 0,2559 | HBE1 | 1,28 | 0,0491 | GTF2H5 | 2,53 | 0,017 |
| ITPR2 | -1,81 | 0,0154 | HBP1 | 3,16 | 0,0464 | GTF3C5 | -1,54 | 0,025 |
| IVNS1ABP | -2,18 | 0,5204 | HDAC5 | 2,36 | 0,2172 | GTPBP2 | -2,42 | 0,0272 |
| IZUMO1 | 1,35 | 0,0282 | HDAC8 | 2,04 | 0,4031 | GTPBP8 | 1,29 | 0,0194 |
| JAK2 | -2,04 | 0,2122 | HDDC2 | -1,93 | 0,0232 | GTSE1 | -2,94 | 0,2554 |
| JAM3 | -2,65 | 0,0596 | HEATR5A | -2,65 | 0,0217 | GUSB | -1,82 | 0,0233 |
| JARID2 | -2,04 | 0,2081 | HECTD3 | 2,71 | 0,0403 | GXYLT2 | 2,17 | 0,2001 |
| JOSD1 | -2,31 | 0,7824 | HECW2 | 2,09 | 0,9788 | H19 | -3,33 | 0,1737 |
| JUNB | -2,68 | 0,3377 | HELLS | 2,09 | 0,8420 | H1FX | -1,9 | 0,0065 |
| KANK4 | -1,29 | 0,0260 | HEPH | -3,74 | 0,6037 | H2AFX | -5,19 | 0,0482 |
| KAT6B | 1 | 0,0476 | HERC1 | 1,34 | 0,0442 | H2AFZ | -3,93 | 0,4255 |
| KAT7 | 2,3 | 0,1043 | HERPUD2 | -1,59 | 0,0241 | HAPLN1 | 15,96 | 0,0455 |
| KCNA1 | 1,44 | 0,0170 | HEXA | 2,55 | 0,3439 | HAS2 | 2,43 | 0,2554 |
| KCNE4 | 2,39 | 0,0508 | HEY2 | -6,18 | 0,7520 | HAS2-AS1 | 2,23 | 0,0192 |
| KCNK2 | 13,35 | 0,1843 | HIBADH | 1,69 | 0,0331 | HAS3 | 2,02 | 0,0394 |
| KCTD15 | -2,6 | 0,4127 | HIPK1 | 2,22 | 0,1342 | HAUS7 | -1,95 | 0,0496 |
| KDM4A | -2,28 | 0,5178 | HIPK3 | -3,01 | 0,0572 | HAUS8 | -2,65 | 0,0522 |
| KDR | -3,53 | 0,1054 | HIST1H1B | 2,85 | 0,2976 | HBD | -1,29 | 0,0373 |
| KHDC1 | -2,18 | 0,0738 | HIST1H2AB | 3,62 | 0,2059 | HDDC2 | 1,57 | 0,0311 |
| KIAA0040 | -11,57 | 0,0119 | HIST1H2AE | 2,68 | 0,3991 | HEATR5A | 2,14 | 0,0272 |
| KIF11 | -2,0 | 0,7042 | HIST1H2AG | 8,81 | 0,1948 | HECTD3 | -1,93 | 0,0103 |
| KIF14 | -2,13 | 0,4904 | HIST1H2AI | 4,92 | 0,1702 | HEG1 | -2,92 | 0,5442 |
| KIF15 | -3,19 | 0,1250 | HIST1H2AJ | 5,72 | 0,2299 | HERC1 | -1,47 | 0,0445 |
| KIF18A | -2,85 | 0,9891 | HIST1H2AL | 3,71 | 0,3043 | HHLA3 | 1,88 | 0,0276 |
| KIF20A | -2,25 | 0,8914 | HIST1H2AM | 2,99 | 0,2346 | HINT3 | 1,31 | 0,0035 |
| KIF23 | -3,97 | 0,6493 | HIST1H2BB | 6,92 | 0,2332 | HIPK1 | -2,27 | 0,0409 |
| KIF26B | -3,46 | 0,8349 | HIST1H2BE | 2,08 | 0,6612 | HIPK4 | 1,32 | 0,0197 |
| KIF3C | 1,68 | 0,0433 | HIST1H2BF | 2,68 | 0,3473 | HIST1H1B | -5,85 | 0,1002 |
| KLF5 | -3,48 | 0,2836 | HIST1H2BG | 9,74 | 0,1106 | HIST1H1E | -2,33 | 0,0209 |
| KLHL2 | -2,34 | 0,0365 | HIST1H2BI | 3,09 | 0,5082 | HIST1H2AB | -3,86 | 0,0536 |
| KLHL21 | 2,06 | 0,3291 | HIST1H2BJ | 2,03 | 0,5042 | HIST1H2AE | -3,84 | 0,0878 |
| KLHL29 | -2,14 | 0,9724 | HIST1H2BK | 2,47 | 0,6804 | HIST1H2AG | -4,25 | 0,0883 |
| KLHL5 | 2,54 | 0,2299 | HIST1H2BL | 18,72 | 0,2228 | HIST1H2AI | -4,59 | 0,14 |
| KNOP1P4 | -1,25 | 0,0359 | HIST1H2BM | 5,13 | 0,2845 | HIST1H2AJ | -8,4 | 0,0727 |
| KNTC1 | -2,22 | 0,4607 | HIST1H3A | 2,7 | 0,1413 | HIST1H2AL | -8,1 | 0,0695 |
| KRCC1 | -1,25 | 0,0428 | HIST1H3B | 8,17 | 0,1992 | HIST1H2AM | -6,05 | 0,0357 |
| KRT18 | 2,18 | 0,5581 | HIST1H3F | 2,41 | 0,2234 | HIST1H2BB | -8,3 | 0,0497 |
| KRT7 | -2,46 | 0,9833 | HIST1H4D | 2,25 | 0,2313 | HIST1H2BE | -2,64 | 0,2045 |
| KRT8 | -7,82 | 0,0427 | HIST1H4E | 2,54 | 0,1570 | HIST1H2BF | -3,55 | 0,105 |
| KRT84 | -1,3 | 0,0280 | HIST1H4I | 2,67 | 0,1328 | HIST1H2BG | -10,22 | 0,0307 |
| KRTAP10-11 | 1,78 | 0,0207 | HIST2H2BE | 2,29 | 0,6268 | HIST1H2BI | -2,57 | 0,1458 |
| KRTAP1-5 | 1,96 | 0,0128 | HIST2H2BF | 14,51 | 0,1619 | HIST1H2BJ | -3,23 | 0,146 |
| KRTAP2-5P | 1,3 | 0,0438 | HJURP | 2,27 | 0,4500 | HIST1H2BK | -4,71 | 0,1642 |
| LACC1 | 2,88 | 0,3192 | HLA-G | 1,77 | 0,0396 | HIST1H2BL | -14,28 | 0,04 |
| LACRT | 1,25 | 0,0446 | HMBS | -1,76 | 0,0470 | HIST1H2BM | -13,05 | 0,0658 |
| LACTB | 2,87 | 0,0001 | HMGB2 | 2,22 | 0,7375 | HIST1H3A | -2,5 | 0,042 |
| LAMA1 | 1,37 | 0,0480 | HMGCR | 2,1 | 0,0879 | HIST1H3B | -6,17 | 0,0902 |
| LAMA5 | 4,69 | 0,3297 | HMGN2 | 2,04 | 0,0732 | HIST1H3F | -3,66 | 0,0276 |
| LAMB2 | 2,73 | 0,6906 | HN1L | 2,15 | 0,0234 | HIST1H3I | -3,18 | 0,0124 |
| LAMC2 | -3,33 | 0,7970 | HNMT | -1,7 | 0,0473 | HIST1H4D | -2,78 | 0,0826 |
| LAMP3 | -2,52 | 0,1227 | HNRNPA2B1 | 2,44 | 0,0960 | HIST1H4E | -8,77 | 0,0191 |
| LAPTM4B | 1,78 | 0,0041 | HNRNPM | 2,98 | 0,1153 | HIST2H2AA3 | 2,19 | 0,1262 |
| LARP7P2 | -1,34 | 0,0285 | HOMER1 | -2,31 | 0,0488 | HIST2H2AB | -4,12 | 0,0594 |
| LDAH | 2,47 | 0,0613 | HPX | 1,6 | 0,0427 | HIST2H2BF | -14,82 | 0,0374 |
| LDLR | 1,96 | 0,0458 | HRCT1 | -2,7 | 0,3026 | HIST2H3A | -2,42 | 0,0531 |
| LGR5 | -4,14 | 0,0285 | HSD11B1 | -1,51 | 0,0219 | HIST4H4 | -1,68 | 0,019 |
| LIF | 2,49 | 0,0506 | HSD17B7 | 2,24 | 0,0156 | HIVEP2 | 2,89 | 0,0641 |
| LIMCH1 | 2,4 | 0,0368 | HSD3B2 | -1,64 | 0,0185 | HLA-DMA | 2,92 | 0,1851 |
| LIN9 | -3,42 | 0,1817 | HSPA4L | 2,96 | 0,3495 | HMGB1 | -2,21 | 0,4351 |
| LINGO1 | 2,06 | 0,1428 | HSPE1-MOB4 | -1,48 | 0,0421 | HMGB2 | -5,89 | 0,1882 |
| LIPG | -2,4 | 0,9571 | HTR2A | -3,3 | 0,5762 | HMGB3 | -2,03 | 0,6423 |
| LIPH | 2,7 | 0,1045 | HYI | 2,86 | 0,0358 | HMGN2 | -2,51 | 0,0216 |
| LMAN2L | -2,57 | 0,9378 | HYKK | -1,39 | 0,0163 | HMGXB3 | -2,36 | 0,3998 |
| LMCD1 | 5,18 | 0,1153 | IBSP | 2,69 | 0,1940 | HMMR | -2,4 | 0,7067 |
| LMNB1 | -3,3 | 0,2391 | ICA1L | 2,48 | 0,0922 | HMOX1 | -2,08 | 0,0296 |
| LMOD1 | 2,11 | 0,1112 | ICAM1 | 2,29 | 0,2166 | HN1L | -2,74 | 0,0301 |
| LONRF1 | -2,02 | 0,0970 | IDI1 | 3,07 | 0,3140 | HNF4G | 1,15 | 0,041 |
| LOXL4 | 3,57 | 0,0811 | IDNK | 3,56 | 0,0551 | HNMT | 2,95 | 0,0406 |
| LPCAT4 | -3,22 | 0,3857 | IFI27 | -4,92 | 0,9121 | HNRNPA0 | -2,59 | 0,0319 |
| LPIN1 | 3,09 | 0,2046 | IFI44 | -3,07 | 0,6543 | HNRNPAB | -2,06 | 0,2682 |
| LPP | 2,27 | 0,0412 | IFI6 | -2,12 | 0,2601 | HNRNPU | -1,71 | 0,0135 |
| LRP5 | 3,82 | 0,0067 | IFIH1 | -2,46 | 0,2906 | HPS5 | 2,66 | 0,103 |
| LRP8 | 2,09 | 0,3322 | IFIT1 | -2,01 | 0,2592 | HPSE | -2,76 | 0,0501 |
| LRRC2 | 1,7 | 0,0382 | IFNAR2 | -2,23 | 0,7467 | HPX | -1,43 | 0,0108 |
| LRRC28 | 4,57 | 0,3599 | IFT74 | -1,44 | 0,0463 | HS3ST3A1 | -1,6 | 0,0248 |
| LRRC32 | 493,19 | 0,1168 | IFT88 | -2,15 | 0,0456 | HSD3B2 | 1,24 | 0,016 |
| LRRCC1 | -2,12 | 0,2438 | IGF2 | 2,6 | 0,5520 | HSF2 | -2,43 | 0,1039 |
| LSAMP | -2,13 | 0,0369 | IGFBP5 | 43,47 | 0,9651 | HSPA2 | 1,67 | 0,0013 |
| LUM | -2,12 | 0,2914 | IKBKB | 3,08 | 0,1615 | HSPA9 | -1,59 | 0,023 |
| LURAP1L | -2,24 | 0,1173 | IKZF5 | 1,92 | 0,0099 | HSPB6 | 2,4 | 0,3083 |
| LUZP2 | -2,32 | 0,0325 | IL13RA2 | -5,34 | 0,0054 | HSPBAP1 | -2,94 | 0,1427 |
| LXN | 2,38 | 0,8744 | IL18BP | 2,82 | 0,1556 | HSPG2 | -2,74 | 0,104 |
| LY6G5C | 1,63 | 0,0125 | IL1A | -3,7 | 0,0765 | HTT | 2,07 | 0,0785 |
| LYAR | -2,16 | 0,2829 | IL1B | -6,15 | 0,5088 | HYI | -2,21 | 0,1013 |
| MAD2L1 | -2,68 | 0,7988 | IL1RAP | 3,34 | 0,2637 | HYLS1 | -2,37 | 0,4383 |
| MAGEB16 | 1,35 | 0,0221 | IL1RAPL2 | -1,6 | 0,0468 | HYOU1 | -2,15 | 0,0538 |
| MAGI1 | -2,05 | 0,9256 | IL1RL1 | -2,11 | 0,6545 | IBSP | -2,29 | 0,8259 |
| MAGI2-AS3 | 2,18 | 0,0009 | IL1RN | -1,92 | 0,0463 | ID1 | -2,94 | 0,0779 |
| MAGT1 | 1,72 | 0,0442 | IL20RB | -2,43 | 0,1930 | IDH3A | -1,77 | 0,0425 |
| MALL | -7,58 | 0,0279 | IL22RA2 | -1,52 | 0,0377 | IDNK | -2,49 | 0,1018 |
| MALT1 | 2,25 | 0,1121 | IL4R | 3,15 | 0,0796 | IDS | 1,64 | 0,0247 |
| MAOA | 2,04 | 0,4477 | IL6 | -3,33 | 0,2375 | IFI27L2 | 1,45 | 0,0282 |
| MAP2K3 | 1,46 | 0,0232 | IL7 | -3,04 | 0,1233 | IFI44 | 2,44 | 0,7887 |
| MAP2K4P1 | 1,4 | 0,0217 | IL7R | -2,45 | 0,5151 | IFIT1 | 2,11 | 0,2211 |
| MAP2K5 | 2,98 | 0,2438 | ILDR2 | -1,34 | 0,0145 | IFRD2 | -3,19 | 0,0193 |
| MAP3K1 | 2,48 | 0,4781 | IMPDH2 | 1,28 | 0,0291 | IFT20 | 3,07 | 0,0009 |
| MAP3K11 | -2,16 | 0,7570 | INCENP | 5,21 | 0,2239 | IFT57 | 1,91 | 0,0397 |
| MAP3K12 | 2,3 | 0,2501 | INPP5A | -2,05 | 0,7673 | IFT80 | -2,14 | 0,2565 |
| MAP3K13 | 2,81 | 0,1004 | INSIG1 | 2,32 | 0,0215 | IFT81 | 1,55 | 0,037 |
| MAP3K7CL | 6,9 | 0,0284 | INTS1 | -2,22 | 0,5883 | IGF2 | -1,73 | 0,0347 |
| MAP4K4 | -2,43 | 0,9729 | INTS4 | -1,31 | 0,0346 | IGF2BP3 | 3,12 | 0,7829 |
| MAP4K5 | -1,23 | 0,0356 | INTU | -1,54 | 0,0318 | IGFBP2 | 7,51 | 0,0899 |
| MAPT | 1,23 | 0,0345 | INVS | 2,05 | 0,2661 | IGFBP4 | 2,43 | 0,4039 |
| MARCO | -1,11 | 0,0451 | IQCH | 2,62 | 0,3876 | IGFBP5 | -5,19 | 0,7103 |
| MAT2B | -1,78 | 0,0341 | IQGAP3 | 8,9 | 0,0367 | IGIP | 3,86 | 0,003 |
| MATN2 | 2,87 | 0,1995 | IRAK4 | 2,8 | 0,0912 | IKBKB | -2,44 | 0,3537 |
| MBD3 | -2,13 | 0,5686 | ISLR | -6,53 | 0,5024 | IKBKG | -1,28 | 0,0481 |
| MC4R | -1,26 | 0,0398 | ISYNA1 | 3,56 | 0,3026 | IKZF5 | -1,67 | 0,0151 |
| MCFD2 | 4,19 | 0,0641 | ITGA2 | -2,13 | 0,2789 | IL13RA2 | -2,86 | 0,7669 |
| MCM7 | -2,22 | 0,7115 | ITGA3 | -2,53 | 0,7393 | IL17RD | -2,46 | 0,3981 |
| MCM8 | -3,05 | 0,2422 | ITGA8 | -2,65 | 0,4552 | IL18BP | -1,71 | 0,0436 |
| MDFIC | -1,42 | 0,0372 | ITGB3 | 3,13 | 0,1559 | IL18R1 | -3,49 | 0,0009 |
| MDM1 | -1,53 | 0,0248 | ITGB8 | 6,84 | 0,6444 | IL1A | 3,75 | 0,1507 |
| MDM2 | 5,06 | 0,2090 | ITM2A | -3,3 | 0,0706 | IL1B | 12,11 | 0,0276 |
| ME2 | 1,71 | 0,0187 | ITM2C | 2,59 | 0,8987 | IL1RAP | -2,01 | 0,3482 |
| MECOM | -2,38 | 0,5016 | ITPR2 | -1,46 | 0,0050 | IL1RL1 | -57,35 | 0,0408 |
| MED20 | 2,2 | 0,0705 | IVNS1ABP | -2,03 | 0,9363 | IL4R | -3,53 | 0,0542 |
| MED22 | 1 | 0,0239 | JADE2 | 2,14 | 0,5442 | IL5 | 1,32 | 0,0169 |
| MEDAG | 1,77 | 0,0109 | JAK2 | -2,24 | 0,0702 | IL6 | 2,67 | 0,1157 |
| MEIS1 | -2,47 | 0,2100 | JAM2 | -3,83 | 0,9407 | IL7R | 2,64 | 0,115 |
| MELK | -2,18 | 0,5569 | JAM3 | -2,92 | 0,0101 | IMPDH2 | -1,32 | 0,0396 |
| METAP1 | -2,13 | 0,1565 | KAT7 | 2,76 | 0,1624 | INAFM2 | 2,41 | 0,0327 |
| Metazoa_SRP | -3,39 | 0,9086 | KBTBD12 | -1,73 | 0,0127 | INCENP | -8,42 | 0,1252 |
| METRNL | -2,48 | 0,8559 | KBTBD8 | 2,38 | 0,3440 | ING3 | 2,84 | 0,5332 |
| METTL20 | 1,43 | 0,0096 | KCNAB1 | 4,31 | 0,8108 | INHBA | 2,72 | 0,1697 |
| METTL3 | -2,34 | 0,7731 | KCNIP2 | 1,7 | 0,0039 | INTU | 1,74 | 0,0007 |
| METTL6 | -2,25 | 0,0087 | KCNK1 | 2,03 | 0,4499 | IP6K1 | -2,26 | 0,1017 |
| MFAP2 | -5,61 | 0,0298 | KCNK10 | -1,34 | 0,0447 | IPMK | -2,39 | 0,0243 |
| MFSD1 | 2,61 | 0,0630 | KCNK2 | 8,35 | 0,5256 | IPO9 | -2,79 | 0,0166 |
| MFSD14A | 2,24 | 0,0478 | KCTD21 | 2,37 | 0,2151 | IQCF6 | -1,32 | 0,0439 |
| MFSD6 | 4,31 | 0,2004 | KCTD8 | -2,21 | 0,0461 | IQGAP3 | -4,97 | 0,0313 |
| MGA | -2,19 | 0,1307 | KDELC1 | -2,14 | 0,0458 | IQSEC2 | 1,13 | 0,0491 |
| MGARP | 3,63 | 0,0998 | KDELR3 | -2,74 | 0,6628 | IRAK1BP1 | -1,71 | 0,0322 |
| MGC10814 | -1,34 | 0,0152 | KDM2B | 2,01 | 0,0639 | ISYNA1 | 3,08 | 0,5801 |
| MGP | -8,89 | 0,1571 | KDM3A | 2,02 | 0,1470 | ITGA3 | 3,97 | 0,1046 |
| MGST1 | 2,48 | 0,6190 | KDM4C | 1,36 | 0,0379 | ITGA4 | 1,35 | 0,0233 |
| MGST2 | -2,4 | 0,3587 | KDR | -3,75 | 0,9782 | ITGA5 | -2,31 | 0,1319 |
| MICU1 | 2,35 | 0,3824 | KIF18B | 4,6 | 0,2148 | ITGA6 | -6,52 | 0,0544 |
| MID1 | 2,55 | 0,0415 | KIF1BP | 2,12 | 0,0767 | ITGA9-AS1 | 1,51 | 0,0308 |
| MID2 | -2,27 | 0,3949 | KIF20A | 5,29 | 0,2143 | ITPR3 | -2,86 | 0,0525 |
| MIOS | 3,05 | 0,1020 | KIF22 | 5,27 | 0,1940 | JAM2 | 2,25 | 0,6235 |
| MIS18A | -3,92 | 0,3733 | KIFAP3 | -2,14 | 0,0190 | JOSD1 | -2,74 | 0,0426 |
| MIS18BP1 | -3,65 | 0,1766 | KIFC1 | 2,6 | 0,4291 | KAT6B | 2,02 | 0,0545 |
| MKI67 | -3,03 | 0,5091 | KITLG | -3,04 | 0,0018 | KCNA10 | -1,28 | 0,035 |
| MKS1 | -2,19 | 0,2566 | KLF5 | -2,11 | 0,3352 | KCNAB1 | -6,83 | 0,5714 |
| MLH1 | 2,19 | 0,2045 | KLHL18 | -1,28 | 0,0270 | KCNG3 | 1,27 | 0,0294 |
| MLLT1 | -1,64 | 0,0101 | KLHL20 | 2,16 | 0,1418 | KCNH4 | 1,24 | 0,0332 |
| MLPH | 2,23 | 0,1860 | KLHL5 | 5,01 | 0,0490 | KCNK1 | -2,57 | 0,0121 |
| MMP10 | -2,13 | 0,2332 | KMT5B | -1,93 | 0,0084 | KCNK2 | 2,35 | 0,5184 |
| MMP12 | 2,02 | 0,7862 | KNOP1 | -1,93 | 0,0320 | KCNMB2 | -1,25 | 0,0385 |
| MMP14 | -2,43 | 0,7643 | KNSTRN | 2,69 | 0,0934 | KCNN4 | -2,21 | 0,884 |
| MMP16 | -3,49 | 0,0011 | KPNA2 | 2,5 | 0,5191 | KCTD15 | -3,01 | 0,1224 |
| MMS22L | -3,09 | 0,6116 | KPNA5 | 1,46 | 0,0143 | KCTD4 | 1,13 | 0,0286 |
| MN1 | -4,18 | 0,4842 | KRBOX1 | -17,99 | 0,0386 | KCTD8 | 1,67 | 0,0215 |
| MOCOS | -1,91 | 0,0498 | KRT78 | -1,26 | 0,0246 | KDELR3 | 2,9 | 0,0184 |
| MOK | 2,11 | 0,5383 | KRT8 | -9,18 | 0,0578 | KDM3B | -1,62 | 0,0366 |
| MOXD1 | 4,77 | 0,1076 | KRT84 | -1,74 | 0,0157 | KDM4A | -2,09 | 0,5855 |
| MPC2 | -2,11 | 0,9744 | LACTB2 | -2,07 | 0,1402 | KDM4A-AS1 | -1,35 | 0,0193 |
| MPHOSPH6 | -2,48 | 0,5299 | LAMA4 | 2,37 | 0,0823 | KDR | -2 | 0,1529 |
| MPP4 | 2,24 | 0,1753 | LAMA5 | 2,59 | 0,2416 | KIAA0040 | -2,17 | 0,2386 |
| MPV17 | 1,36 | 0,0293 | LAMB2 | 3,99 | 0,2980 | KIAA0101 | -2,68 | 0,6883 |
| MR1 | 2,68 | 0,1757 | LAMC2 | -4,76 | 0,7398 | KIAA0753 | -1,72 | 0,0192 |
| MRE11A | -2,79 | 0,0168 | LAMTOR2 | -1,59 | 0,0111 | KIAA1024L | 1,28 | 0,0234 |
| MROH8 | 1,45 | 0,0406 | LAMTOR3 | -1,82 | 0,0043 | KIAA1109 | -2,01 | 0,3985 |
| MRPL15 | -2,09 | 0,4566 | LARS2 | 1,4 | 0,0264 | KIAA1324L | -3,24 | 0,2837 |
| MRPS18C | -2,18 | 0,4100 | LAS1L | 2,59 | 0,2335 | KIAA1524 | -10,77 | 0,1283 |
| MRPS27 | 2,62 | 0,6223 | LAYN | -5,41 | 0,0009 | KIAA1549L | -2,52 | 0,0453 |
| MRPS30 | -2,12 | 0,1819 | LBH | -7,3 | 0,9533 | KIDINS220 | -2,09 | 0,0977 |
| MSC-AS1 | 2,05 | 0,8565 | LBR | 3,49 | 0,0677 | KIF11 | -2,57 | 0,2923 |
| MSI2 | 2,1 | 0,0651 | LDB2 | 3,94 | 0,2239 | KIF14 | -3,57 | 0,2863 |
| MSMO1 | 2,11 | 0,0016 | LDLR | 4,14 | 0,0439 | KIF15 | -2,71 | 0,1738 |
| MSR1 | 1,52 | 0,0440 | LGALS8 | 3,32 | 0,1119 | KIF18A | -3,21 | 0,3102 |
| MT1A | 4,09 | 0,0313 | LGR4 | -1,39 | 0,0479 | KIF18B | -5,17 | 0,0607 |
| MT1E | 2,59 | 0,1939 | LGR5 | -2,66 | 0,1701 | KIF20A | -6,57 | 0,1763 |
| MT1F | 3,93 | 0,1592 | LIN37 | -1,46 | 0,0101 | KIF22 | -8,73 | 0,1153 |
| MT1G | 3,57 | 0,0811 | LIN52 | 2,22 | 0,1119 | KIF23 | -4,3 | 0,3845 |
| MT1H | 3,03 | 0,0957 | LINGO1 | 2,71 | 0,0361 | KIF24 | -2,41 | 0,2546 |
| MT1JP | 4,3 | 0,0275 | LINS1 | 5,38 | 0,0141 | KIF25 | 1,89 | 0,035 |
| MT1L | 4,76 | 0,0301 | LIPH | -2,27 | 0,7447 | KIF26B | -4,57 | 0,6462 |
| MT1M | 2,69 | 0,0799 | LOXL4 | 2,04 | 0,1624 | KIF4A | -2,04 | 0,6438 |
| MT1P1 | 2,83 | 0,0473 | LPCAT3 | 2,08 | 0,1046 | KIFAP3 | 2,09 | 0,4216 |
| MT1X | 3,22 | 0,2134 | LPCAT4 | 2,18 | 0,3920 | KIFC1 | -3,85 | 0,1776 |
| MT2A | 3,56 | 0,1810 | LPXN | 2,54 | 0,1151 | KITLG | 2,54 | 0,012 |
| MTATP6P7 | -1,59 | 0,0228 | LRBA | 1,43 | 0,0351 | KLF6 | -1,65 | 0,015 |
| MTCO3P17 | 1,47 | 0,0106 | LRP12 | -2,14 | 0,5352 | KLHDC3 | -2,17 | 0,069 |
| MTCYBP1 | -1,51 | 0,0163 | LRP5 | 2,37 | 0,0578 | KLHL35 | 1,23 | 0,042 |
| MTERF2 | -2,24 | 0,1727 | LRP6 | 1 | 0,0018 | KLHL5 | -2,18 | 0,1242 |
| MTND1P33 | -1,42 | 0,0168 | LRP8 | 6,6 | 0,1320 | KLK13 | -1,42 | 0,0281 |
| MTND1P35 | -1,55 | 0,0360 | LRRC32 | 22,99 | 0,1876 | KNCN | -1,43 | 0,0183 |
| MTND1P4 | -1,32 | 0,0172 | LRRC34 | -2,29 | 0,3178 | KPNA2 | -4,53 | 0,3545 |
| MTND4P16 | 4,44 | 0,0383 | LRRC8B | -2,26 | 0,5804 | KRBOX1 | 7,73 | 0,0252 |
| MT-TC | -2,91 | 0,0031 | LRRD1 | -1,32 | 0,0224 | KRIT1 | -1,49 | 0,0163 |
| MTX1 | -2,32 | 0,7846 | LRRN3 | -2,19 | 0,0115 | KRT7 | -5,91 | 0,257 |
| MUSK | 1,29 | 0,0438 | LSM1 | -2,3 | 0,0182 | KRT80 | -1,45 | 0,0052 |
| MXD3 | 1,58 | 0,0330 | LSS | 1,62 | 0,0317 | KRTAP1-5 | 1,69 | 0,0009 |
| MXRA5 | -3,39 | 0,2652 | LST1 | -1,24 | 0,0454 | LACC1 | 3,52 | 0,7747 |
| MYC | -3,31 | 0,9589 | LTBP1 | -2,14 | 0,1612 | LACTB | 2,32 | 0,0041 |
| MYCBPAP | 1,5 | 0,0210 | LXN | 4,92 | 0,7241 | LAMA2 | -2,02 | 0,7816 |
| MYEF2 | 2,54 | 0,0110 | LYRM4 | 2,11 | 0,0248 | LAMA4 | -2,19 | 0,189 |
| MYL9 | 4,08 | 0,0486 | LYRM7 | -2,05 | 0,0200 | LAMTOR3 | 2,28 | 0,0197 |
| MYLK | 37,24 | 0,0775 | MAB21L1 | -2,02 | 0,0328 | LANCL2 | 2,06 | 0,0511 |
| MYO1D | 3,91 | 0,2650 | MADD | 1,41 | 0,0097 | LAP3 | 2,3 | 0,8916 |
| MYO1E | 2,09 | 0,0238 | MAGEB2 | -1,36 | 0,0238 | LARS2 | -1,9 | 0,0239 |
| MYOCD | 9,93 | 0,0829 | MAGED4 | -2,23 | 0,9809 | LAS1L | -2,86 | 0,0705 |
| MYOZ2 | 2,6 | 0,0967 | MAGEH1 | -1,63 | 0,0459 | LAYN | 3,53 | 0,007 |
| NABP1 | 5,0 | 0,0744 | MAGI1 | -2,37 | 0,9091 | LBH | 6,68 | 0,4335 |
| NABP2 | 1,77 | 0,0336 | MAML2 | -2,48 | 0,0618 | LBR | -2,62 | 0,0498 |
| NACAD | -1,61 | 0,0067 | MAOB | -2,22 | 0,2543 | LCN12 | -1,2 | 0,0467 |
| NAP1L1 | -1,29 | 0,0378 | MAP1A | -2,37 | 0,7745 | LDB2 | -6,57 | 0,0945 |
| NAP1L1P2 | -1,62 | 0,0385 | MAP1B | -3,99 | 0,3526 | LDLR | -2,19 | 0,4503 |
| NAP1L5 | 2,03 | 0,0538 | MAP1LC3B | -2,14 | 0,0423 | LETMD1 | 2,16 | 0,0185 |
| NARS2 | -2,06 | 0,0096 | MAP1S | -2,22 | 0,9168 | LGALS3BP | 2,7 | 0,24 |
| NBEA | 2,26 | 0,0472 | MAP2K5 | 3,22 | 0,0763 | LGALS4 | 1,34 | 0,0481 |
| NBEAL1 | 2,21 | 0,0034 | MAP3K1 | 3,64 | 0,1708 | LHPP | 1,52 | 0,0159 |
| NBPF14 | 1,78 | 0,0310 | MAP3K7CL | 4,34 | 0,0120 | LIMCH1 | 2,3 | 0,047 |
| NBPF19 | 1,92 | 0,0070 | MAP9 | -2,1 | 0,0826 | LIMD2 | -1,2 | 0,0398 |
| NBR2 | -1,6 | 0,0191 | MAPK14 | 2,02 | 0,0605 | LIN37 | 1,73 | 0,0458 |
| NCAPG | -4,06 | 0,3487 | MASP1 | 2,16 | 0,1044 | LIN9 | -4,81 | 0,0738 |
| NCAPG2 | 2,24 | 0,0874 | MAST4 | -2,21 | 0,7139 | LINC01085 | 2,03 | 0,2123 |
| NCAPH | -3,75 | 0,6265 | MATN2 | 2,16 | 0,8278 | LINC01588 | 3,01 | 0,0163 |
| NCEH1 | 5,77 | 0,2116 | MCHR1 | -1,42 | 0,0114 | LINS1 | -2,72 | 0,0539 |
| NCOA3 | 1,95 | 0,0359 | MCM2 | 7,56 | 0,1483 | LIPH | 5,72 | 0,0186 |
| NDC80 | -5,07 | 0,4796 | MCM3 | 2,85 | 0,1279 | LMAN2L | -3,07 | 0,0754 |
| NDE1 | -2,43 | 0,4557 | MCM5 | 8,23 | 0,1752 | LMBR1L | -1,77 | 0,0052 |
| NDRG3 | 2,08 | 0,0249 | MCM6 | 2,81 | 0,1418 | LMCD1 | 8,68 | 0,3769 |
| NDUFA10 | 1 | 0,0386 | MCM7 | 3,41 | 0,2470 | LMNB1 | -2 | 0,6505 |
| NDUFA6 | 1 | 0,0361 | MDM2 | 4,98 | 0,1750 | LMNB2 | -1,22 | 0,048 |
| NDUFA8 | 1,71 | 0,0459 | ME2 | 2,14 | 0,0485 | LMO7 | 3,34 | 0,0911 |
| NDUFS4 | 1,59 | 0,0026 | MED22 | 1 | 0,0193 | LMO7DN | 1,31 | 0,0089 |
| NDUFS6P1 | 1,5 | 0,0205 | MEDAG | 2,55 | 0,2223 | LNX2 | -2,01 | 0,1947 |
| NDUFV2 | 1,52 | 0,0385 | MEIS2 | 2,51 | 0,0288 | LOC102723897 | -2,24 | 0,0737 |
| NEAT1 | 2,21 | 0,2429 | MEIS3 | 2,28 | 0,5858 | LOC730101 | 2,19 | 0,2671 |
| NECAP1 | 1,62 | 0,0247 | METTL4 | 2 | 0,6941 | LONP1 | -2,15 | 0,1472 |
| NEFM | 133,19 | 0,1601 | METTL5 | -1,84 | 0,0261 | LOXL4 | 2,03 | 0,3659 |
| NEGR1 | 2,77 | 0,0944 | MFAP3 | 2,28 | 0,1267 | LPCAT1 | -1,49 | 0,042 |
| NELFCD | 2,13 | 0,1277 | MFAP3L | -2,15 | 0,8867 | LPCAT4 | -4,61 | 0,0402 |
| NFATC3 | 3,86 | 0,1443 | MFAP4 | 2,34 | 0,8274 | LPIN2 | 1,77 | 0,0004 |
| NFKBIA | 2,76 | 0,5992 | MFSD14A | 1,37 | 0,0490 | LPP | 2,56 | 0,2295 |
| NFYB | -2,27 | 0,0740 | MGARP | 6,79 | 0,1130 | LRIT1 | 1,31 | 0,0318 |
| NHEJ1 | 2,45 | 0,0463 | MGP | -2,94 | 0,5813 | LRP2BP | 1,3 | 0,0139 |
| NIFK-AS1 | 2,19 | 0,1589 | MGST1 | 3,57 | 0,3737 | LRP8 | -3,02 | 0,0016 |
| NIN | 2,39 | 0,0271 | MGST2 | -2,56 | 0,4727 | LRRC2 | 1,77 | 0,028 |
| NIPAL2 | 2,72 | 0,7409 | MICAL1 | -2,19 | 0,8367 | LRRC28 | 4,27 | 0,7754 |
| NKTR | 2,15 | 0,7890 | MICU3 | -2,24 | 0,1723 | LRRC32 | 32,12 | 0,4692 |
| NLGN1 | 2,01 | 0,0167 | MID1 | 2,23 | 0,0680 | LRRC34 | 2,26 | 0,1513 |
| NLGN4Y | 6,02 | 0,6715 | MILR1 | -2,07 | 0,3031 | LRRC39 | 1,33 | 0,0102 |
| NOC3L | -2,2 | 0,0203 | MINCR | -1,67 | 0,0210 | LRRC8C | -2,75 | 0,1759 |
| NOS3 | -1,24 | 0,0473 | MIP | -1,36 | 0,0353 | LRRC8D | -3,03 | 0,0061 |
| NOTCH2 | 1,55 | 0,0340 | MIS12 | 4,41 | 0,0203 | LRRN3 | 2,92 | 0,0419 |
| NOTCH2NL | 1,59 | 0,0172 | MIS18A | -2 | 0,5695 | LSM1 | 1,46 | 0,0499 |
| NPAS2 | 3,23 | 0,1596 | MLF1 | -1,45 | 0,0304 | LTBP1 | 2,53 | 0,0258 |
| NPL | 2,15 | 0,3139 | MLLT11 | -2,05 | 0,0526 | LTBP4 | -2,38 | 0,0267 |
| NPM2 | 1,33 | 0,0338 | MLLT6 | 1,57 | 0,0327 | LURAP1L | -2,68 | 0,0946 |
| NPNT | -2,01 | 0,2235 | MMAA | -2,46 | 0,0668 | LXN | -2,06 | 0,6933 |
| NPR3 | 2,56 | 0,2953 | MMACHC | 2 | 0,0553 | LY96 | 2,25 | 0,1742 |
| NQO1 | 2,45 | 0,0707 | MME | 3,25 | 0,0667 | LYPLAL1 | 2,08 | 0,139 |
| NR2C1 | 2,29 | 0,0074 | MMP1 | 2,75 | 0,1719 | LYRM4 | -1,52 | 0,0015 |
| NR2F1 | -2,6 | 0,9845 | MMP14 | -2,16 | 0,7090 | LYRM5 | 1,83 | 0,0185 |
| NR2F2 | 2,32 | 0,2523 | MOB4 | -1,43 | 0,0493 | LYSMD3 | 1,89 | 0,0059 |
| NR3C2 | 2,0 | 0,1452 | MPC2 | -2,43 | 0,4993 | LYSMD4 | -1,69 | 0,0281 |
| NREP | 2,08 | 0,7459 | MRAS | 2,08 | 0,9156 | MALL | -14,24 | 0,0308 |
| NRP2 | 7,68 | 0,0295 | MRC2 | -2,09 | 0,6788 | MAML1 | -2,11 | 0,1989 |
| NRXN3 | 5,98 | 0,1382 | MRE11A | -2,71 | 0,0554 | MAML2 | 2,52 | 0,0643 |
| NSDHL | 2,57 | 0,2227 | MRLN | 1,34 | 0,0306 | MAN2C1 | -2 | 0,0661 |
| NSUN3 | 2,16 | 0,1991 | MRPL11 | 2,42 | 0,0399 | MAOA | 2,24 | 0,0739 |
| NT5DC1 | 1,32 | 0,0280 | MRPL18 | 2,07 | 0,9820 | MAP1A | 2,52 | 0,082 |
| NTF3 | 3,7 | 0,0023 | MRPL32 | -2 | 0,1703 | MAP1B | 2,16 | 0,0616 |
| NTNG1 | -2,22 | 0,8356 | MRPS27 | 7,75 | 0,0865 | MAP3K13 | 2,8 | 0,1308 |
| NTS | -138,5 | 0,0144 | MRPS33 | -1,82 | 0,0232 | MAP3K14 | -1,57 | 0,0464 |
| NTSR1 | -2,42 | 0,1627 | MRPS36 | -2,52 | 0,0559 | MAP3K7CL | 2,04 | 0,9101 |
| NUBPL | 1,6 | 0,0467 | MRRF | 2,07 | 0,0067 | MAP4K3 | 1,33 | 0,0126 |
| NUDCD3 | -2,56 | 0,7586 | MRTO4 | 1,62 | 0,0205 | MAPRE3 | 1,77 | 0,0394 |
| NUF2 | -3,29 | 0,4138 | MSH2 | 2,32 | 0,8179 | MARK3 | -2,22 | 0,0788 |
| NUP133 | -1,85 | 0,0441 | MSH6 | -1,96 | 0,0408 | MAST4 | 2,12 | 0,4054 |
| NUP85 | -3,05 | 0,2533 | MSL2 | 2,27 | 0,0517 | MASTL | -2,87 | 0,2352 |
| NUSAP1 | -2,39 | 0,5867 | MSRB3 | -2,2 | 0,2923 | MAT2A | -2,74 | 0,1725 |
| NXF2B | -1,38 | 0,0260 | MT1E | -2,41 | 0,5937 | MAT2B | -1,99 | 0,042 |
| NXPE4 | -1,17 | 0,0470 | MTA2 | 2,34 | 0,0941 | MAZ | -2,08 | 0,1185 |
| NYNRIN | 2,34 | 0,1720 | MTAP | 2,16 | 0,2044 | MB | 1,18 | 0,0466 |
| ODF2L | -2,06 | 0,0371 | MTBP | 3,5 | 0,1100 | MBD2 | -1,43 | 0,0465 |
| OFD1P3Y | -2,59 | 0,0064 | MTHFD1 | 5,32 | 0,1143 | MBD3 | -1,7 | 0,0497 |
| OGN | -5,22 | 0,0080 | MTHFSD | 1,63 | 0,0253 | MCEE | 1,86 | 0,001 |
| OIP5 | -2,21 | 0,8829 | MTMR3 | 2,61 | 0,0625 | MCFD2 | 2,39 | 0,3864 |
| OLA1 | 2,16 | 0,1131 | MTMR4 | -2,58 | 0,3425 | MCM2 | -7,86 | 0,067 |
| OMG | -1,32 | 0,0161 | MTRR | 2,06 | 0,3076 | MCM3 | -8,2 | 0,0306 |
| OPA1 | 2,06 | 0,0102 | MTSS1 | 2,07 | 0,4458 | MCM5 | -14,06 | 0,044 |
| OPRK1 | -1,7 | 0,0388 | MT-TC | -1,79 | 0,0317 | MCM6 | -3,64 | 0,0588 |
| OPTN | 2,49 | 0,0325 | MTUS1 | -3,04 | 0,6512 | MCM7 | -9,95 | 0,1884 |
| OR10G7 | -1,19 | 0,0376 | MUC12 | -1,42 | 0,0405 | MCMBP | -1,83 | 0,0165 |
| OR14I1 | -1,44 | 0,0183 | MX1 | -2,16 | 0,2700 | MCU | -2,03 | 0,8455 |
| OR2A9P | -2,24 | 0,0294 | MYBL2 | 13,17 | 0,1307 | MDK | -2,47 | 0,1458 |
| OR2AG2 | -1,5 | 0,0236 | MYEOV2 | -2,42 | 0,2661 | MDM4 | -2,11 | 0,0695 |
| OR2T29 | -1,36 | 0,0154 | MYLIP | 1,9 | 0,0340 | MECR | -2,4 | 0,0191 |
| OR4R2P | 1,11 | 0,0493 | MYLK | 3,04 | 0,6594 | MED12 | -1,87 | 0,0161 |
| OR51B4 | 1,44 | 0,0153 | MYO10 | -1,34 | 0,0454 | MED12L | -2,17 | 0,0171 |
| OR51H1 | 1,23 | 0,0397 | MYO1D | 5,29 | 0,1334 | MED30 | -1,73 | 0,031 |
| OR56A1 | -1,73 | 0,0039 | MYOM1 | -1,29 | 0,0266 | MEIS1 | -4,68 | 0,6134 |
| OR56B4 | 1,68 | 0,0033 | NABP1 | 4,69 | 0,2080 | MEP1B | 1,44 | 0,0329 |
| OR6B2 | 1,43 | 0,0087 | NACAD | -1,61 | 0,0488 | MEPCE | -3,45 | 0,1011 |
| OR6T1 | -1,26 | 0,0352 | NADSYN1 | 1,32 | 0,0380 | Metazoa_SRP | -2,67 | 0,2092 |
| OR7E163P | 1,32 | 0,0374 | NAE1 | 2,19 | 0,0959 | METRNL | -4,53 | 0,0507 |
| OR7L1P | -1,3 | 0,0308 | NANOGNB | -1,5 | 0,0461 | METTL13 | -2,25 | 0,0226 |
| ORMDL3 | 2,72 | 0,2730 | NAP1L3 | -2,02 | 0,0087 | METTL4 | -2,81 | 0,1589 |
| OSGIN1 | 1,4 | 0,0472 | NAT1 | 1,97 | 0,0386 | MFAP2 | -3,2 | 0,997 |
| OSTF1 | 2,04 | 0,3417 | NAT10 | 1,8 | 0,0444 | MFAP3L | 3,31 | 0,5218 |
| OSTM1 | 1,77 | 0,0114 | NBPF1 | -2,34 | 0,6367 | MFAP4 | -2,98 | 0,5771 |
| OXCT1 | 2,17 | 0,5917 | NBPF3 | 2,16 | 0,0708 | MFSD6 | 3,55 | 0,4678 |
| P2RY13 | 1,31 | 0,0183 | NCAPD2 | 4,27 | 0,1463 | MFSD6L | -1,22 | 0,0233 |
| P3H2 | 7,96 | 0,0729 | NCBP2 | -1,65 | 0,0079 | MGAT1 | -1,4 | 0,0108 |
| P4HA2 | -3,87 | 0,8989 | NCEH1 | 2,37 | 0,6391 | MGAT3 | 1,2 | 0,0494 |
| PADI4 | 1 | 0,0475 | NCOA1 | -2,33 | 0,0338 | MGP | -2,18 | 0,3406 |
| PAG1 | 2,7 | 0,8900 | NCOA3 | 1,67 | 0,0253 | MID2 | -1,74 | 0,0112 |
| PAK3 | -2,82 | 0,1719 | NCOA5 | 2,71 | 0,0726 | MIEN1 | 1,58 | 0,031 |
| PALLD | 3,6 | 0,1015 | NCOA6 | -2,05 | 0,4383 | MILR1 | 2,41 | 0,3291 |
| PALMD | 10,5 | 0,0169 | NDNF | -2,75 | 0,0036 | MIS12 | -2,76 | 0,023 |
| PAMR1 | 4,57 | 0,0645 | NDRG1 | 2,38 | 0,1235 | MKI67 | -4,2 | 0,3454 |
| PANK1 | 2,94 | 0,0382 | NDUFA4L2 | -6,94 | 0,1944 | MKS1 | -1,47 | 0,0218 |
| PAPPA2 | -2,16 | 0,2229 | NDUFA6-AS1 | -2,18 | 0,0046 | MLEC | 1,71 | 0,0181 |
| PAPSS2 | 2,34 | 0,0275 | NDUFAF5 | -2,25 | 0,0418 | MLLT1 | -1,88 | 0,0065 |
| PARD6G-AS1 | 1,32 | 0,0326 | NEAT1 | 3,25 | 0,0122 | MLPH | 2,26 | 0,1635 |
| PARP4P2 | 1,56 | 0,0107 | NEDD4 | 2,01 | 0,0997 | MMAA | 2,47 | 0,045 |
| PAXBP1 | 3,17 | 0,1191 | NEFM | -4,16 | 0,0356 | MMP10 | -2,04 | 0,0548 |
| PCCB | 2,36 | 0,6102 | NEGR1 | 2,16 | 0,0006 | MMP16 | -2,92 | 0,8777 |
| PCDH7 | -6,75 | 0,0829 | NELFCD | 2,4 | 0,1426 | MMP9 | -1,14 | 0,0364 |
| PCDHB14 | -2,19 | 0,0912 | NEO1 | -3,67 | 0,3183 | MMS22L | -2,43 | 0,4625 |
| PCED1B-AS1 | -1,39 | 0,0173 | NFATC3 | 3,94 | 0,0256 | MN1 | -3,07 | 0,883 |
| PCK2 | -2,14 | 0,6860 | NFIA | 2,3 | 0,1409 | MOK | 2,89 | 0,3589 |
| PCNXL4 | 1,45 | 0,0182 | NFKB1 | 1,9 | 0,0345 | MOXD1 | 5,58 | 0,0142 |
| PCYOX1 | 2,02 | 0,1002 | NFKBIZ | -2,74 | 0,3105 | MPHOSPH8 | -1,67 | 0,0342 |
| PDE10A | 3,24 | 0,0531 | NFYB | -2,54 | 0,0061 | MRPL11 | -1,56 | 0,0195 |
| PDE1C | 9,95 | 0,1410 | NGDN | 2,63 | 0,1554 | MRPL15 | -2,12 | 0,5455 |
| PDE5A | 4,05 | 0,0025 | NHEJ1 | 2,89 | 0,1099 | MRPL33 | 1,58 | 0,0319 |
| PDE7B | 2,57 | 0,0083 | NHSL1 | 3,23 | 0,0850 | MRPS25 | -1,4 | 0,0204 |
| PDGFC | 2,51 | 0,0222 | NID1 | 3,62 | 0,2258 | MRPS27 | -2,37 | 0,0732 |
| PDGFRB | 5,5 | 0,4356 | NID2 | -2,51 | 0,1344 | MRPS33 | 1,54 | 0,0198 |
| PDK1 | 1,81 | 0,0467 | NKAP | -2,19 | 0,0667 | MRVI1 | 1,87 | 0,0226 |
| PDLIM1 | 3,44 | 0,2817 | NLRP10 | 1 | 0,0255 | MSANTD3-TMEFF1 | -2,15 | 0,1704 |
| PDLIM2 | 5,98 | 0,1211 | NME5 | -3,95 | 0,0114 | MSGN1 | 1,29 | 0,0403 |
| PDPR | 2,3 | 0,4094 | NMT2 | 2,57 | 0,0361 | MSH2 | -2,11 | 0,5367 |
| PDSS1 | -3,15 | 0,2669 | NOC2L | -2,06 | 0,4343 | MSRB2 | 2,75 | 0,0503 |
| PDZD9 | -1,45 | 0,0155 | NOD1 | 2,25 | 0,0995 | MSRB3 | 2,29 | 0,053 |
| PEBP1 | 2,0 | 0,2599 | NOTCH2NL | -1,55 | 0,0478 | MT1A | 3,59 | 0,0371 |
| PERP | 2,15 | 0,2477 | NPAS2 | 3,64 | 0,0958 | MT1E | 7,19 | 0,0854 |
| PEX12 | 1,67 | 0,0246 | NPIPB4 | 1,34 | 0,0352 | MT1F | 5,79 | 0,0858 |
| PGM5 | 2,37 | 0,0334 | NPNT | -2,18 | 0,0748 | MT1G | 3,33 | 0,053 |
| PGM5-AS1 | 2,96 | 0,0893 | NPR3 | 5,95 | 0,8684 | MT1H | 3,62 | 0,0547 |
| PGPEP1 | 2,11 | 0,0479 | NPTN | -2,11 | 0,1643 | MT1X | 2,91 | 0,125 |
| PGRMC1 | 1,62 | 0,0392 | NQO1 | 3,79 | 0,0364 | MT2A | 4,76 | 0,0547 |
| PHB2 | -2,24 | 0,7389 | NR2C1 | 2,63 | 0,1088 | MTA2 | -2,14 | 0,0076 |
| PHBP14 | -1,39 | 0,0364 | NR4A1 | 7,42 | 0,5436 | MTBP | -2,8 | 0,0603 |
| PHF3 | -2,27 | 0,1746 | NR4A3 | 7,13 | 0,6647 | MTHFD1 | -2,81 | 0,014 |
| PHF5CP | -1,88 | 0,0342 | NRAS | 1,64 | 0,0430 | MTIF2 | 2,06 | 0,0007 |
| PHF7 | 1,61 | 0,0299 | NRCAM | -3,9 | 0,8788 | MTMR3 | -2,2 | 0,0816 |
| PHGDH | -2,4 | 0,4153 | NREP | 3,29 | 0,3275 | MTMR7 | 1,43 | 0,0305 |
| PHLDB1 | 2,49 | 0,4071 | NRM | 5,24 | 0,2631 | MT-TA | 1,88 | 0,0392 |
| PI15 | -2,52 | 0,0600 | NRP2 | 2,72 | 0,0623 | MT-TS1 | 2,44 | 0,0864 |
| PI16 | -2,4 | 0,6163 | NRSN1 | -1,41 | 0,0255 | MTUS1 | 2,75 | 0,4132 |
| PID1 | 1,37 | 0,0243 | NSDHL | 2,35 | 0,3130 | MTX1 | -2,06 | 0,1014 |
| PIEZO1P1 | 1,83 | 0,0440 | NSF | -2,38 | 0,0052 | MUC15 | 1,38 | 0,0293 |
| PIGK | 1,65 | 0,0483 | NTM | -2,76 | 0,2630 | MUT | -1,68 | 0,0484 |
| PIH1D1 | -2,06 | 0,7358 | NTRK3 | 2,02 | 0,6833 | MYBL2 | -9,46 | 0,0113 |
| PIK3C2A | 1,54 | 0,0091 | NTS | -177,96 | 0,0508 | MYC | -3,14 | 0,0727 |
| PIK3R1 | -2,03 | 0,1069 | NUAK1 | 3,28 | 0,6247 | MYEF2 | 3,07 | 0,1581 |
| PIK3R3 | 1,75 | 0,0453 | NUDCD1 | 4,9 | 0,0287 | MYL9 | 5,08 | 0,0723 |
| PKIA | -2,37 | 0,1864 | NUFIP1 | -2,02 | 0,2813 | MYLK | 13,67 | 0,1563 |
| PKNOX2 | 2,56 | 0,0545 | NUP133 | -1,94 | 0,0235 | MYO1E | 1,78 | 0,0325 |
| PKP2 | -2,18 | 0,8603 | NUP35 | 2,48 | 0,6069 | MYO1G | -1,35 | 0,0192 |
| PLAT | 13,13 | 0,8225 | NUP43 | 1,82 | 0,0107 | MYOCD | 8 | 0,0732 |
| PLBD1 | -1,94 | 0,0087 | NUP62 | 2,02 | 0,1635 | MYOM1 | 1,23 | 0,0462 |
| PLCE1 | -1,52 | 0,0318 | NUP88 | 2,1 | 0,2575 | MYOZ2 | 3,1 | 0,0592 |
| PLEKHG5 | -2,11 | 0,5149 | NUPL2 | -2,42 | 0,2536 | NAA16 | -1,82 | 0,0284 |
| PLK4 | -5,05 | 0,4109 | NUSAP1 | 3,48 | 0,6150 | NAAA | 2,08 | 0,0462 |
| PLOD2 | 2,56 | 0,0754 | NXF1 | 2,36 | 0,0043 | NAP1L3 | 1,88 | 0,0463 |
| PLPP3 | 4,6 | 0,2013 | NXPH4 | -2,03 | 0,5788 | NAP1L5 | 2,47 | 0,0064 |
| PLPPR4 | 13,67 | 0,2154 | NYNRIN | 2,2 | 0,1294 | NARS | -2,17 | 0,0222 |
| PLSCR4 | 6,27 | 0,0090 | OARD1 | -2,39 | 0,0751 | NARS2 | -2,28 | 0,0394 |
| PLXNA4 | 3,22 | 0,4265 | OBFC1 | -1,47 | 0,0257 | NAT10 | -2,51 | 0,0583 |
| PLXNC1 | 2,5 | 0,2901 | OCIAD2 | -4,6 | 0,2711 | NBEA | 2,66 | 0,0263 |
| PLXND1 | 2,12 | 0,6321 | OCLN | 2,45 | 0,2447 | NBEAL1 | 1,44 | 0,0164 |
| PMCH | -2,12 | 0,0898 | OFD1 | -2,21 | 0,1413 | NBPF19 | 2,44 | 0,0002 |
| PMP22 | -4,04 | 0,6219 | OGN | -6,05 | 0,0146 | NBPF3 | -2,74 | 0,0068 |
| PMS2P10 | -1,6 | 0,0127 | OLFM1 | -2,01 | 0,2629 | NCALD | 1,39 | 0,0119 |
| PMS2P5 | -1,52 | 0,0373 | OPN3 | 2,04 | 0,8013 | NCAPD2 | -4,49 | 0,0792 |
| PMS2P9 | -1,4 | 0,0436 | OPRK1 | -1,52 | 0,0498 | NCAPG | -5,2 | 0,1647 |
| PNP | -2,71 | 0,1987 | OR10A3 | 1 | 0,0422 | NCAPH | -3,03 | 0,2201 |
| PODNL1 | -2,77 | 0,3618 | OR10H5 | -1,36 | 0,0254 | NCEH1 | 2,5 | 0,4772 |
| PODXL | -32,28 | 0,6126 | OR10W1 | -1,21 | 0,0489 | NCL | -1,57 | 0,0245 |
| POLA1 | -2,51 | 0,1679 | OR14I1 | -1,46 | 0,0057 | NDC80 | -3,1 | 0,3835 |
| POLE2 | -2,11 | 0,6650 | OR2J2 | -1,68 | 0,0011 | NDE1 | -2 | 0,0332 |
| POLG2 | -2,15 | 0,0295 | OR2T1 | -1,37 | 0,0430 | NDUFA13 | 1,38 | 0,0423 |
| POLR1D | -2,22 | 0,0421 | OR4F3 | -1,54 | 0,0224 | NDUFA4L2 | 7,8 | 0,0959 |
| POLR3D | -1,37 | 0,0492 | OR51B4 | 1,33 | 0,0344 | NDUFA6-AS1 | 1,6 | 0,0397 |
| POLR3GP2 | -1,6 | 0,0353 | OR5D16 | -1,72 | 0,0278 | NDUFA7 | 1,66 | 0,0106 |
| POM121L12 | 1,42 | 0,0253 | OR5P3 | -1,33 | 0,0240 | NDUFS5 | 1,12 | 0,0485 |
| PON2 | 1,89 | 0,0030 | OR6C65 | 1,54 | 0,0149 | NECAP1 | 1,9 | 0,0373 |
| POP1 | -2,51 | 0,2906 | ORC4 | 1,96 | 0,0269 | NEDD4 | -1,66 | 0,0222 |
| POPDC3 | -3,94 | 0,1054 | OSCP1 | -1,44 | 0,0390 | NEFM | 655,21 | 0,0352 |
| PPBPP1 | 1,27 | 0,0469 | OSGEP | 3,89 | 0,1084 | NEIL2 | 1,29 | 0,041 |
| PPFIA4 | 4,13 | 0,0720 | OSR1 | 2,4 | 0,2583 | NEK1 | 1,54 | 0,0346 |
| PPFIBP2 | -2,23 | 0,5153 | OSR2 | 5,17 | 0,1616 | NELL2 | 1,31 | 0,037 |
| PPM1D | -1,83 | 0,0314 | OTUD4 | -2,06 | 0,1120 | NEO1 | 3 | 0,5202 |
| PPP1R14C | -2,08 | 0,0076 | OXCT1 | 2,6 | 0,5317 | NETO2 | -2,01 | 0,6775 |
| PPP1R2P2 | 1,18 | 0,0437 | P3H1 | -2,34 | 0,6747 | NEXN | 2,51 | 0,2284 |
| PPP3CB-AS1 | -2,23 | 0,0738 | P3H2 | -4,48 | 0,0358 | NFIA | -2,43 | 0,025 |
| PQLC2L | 2,51 | 0,2593 | P4HA1 | -2,2 | 0,0623 | NFKB1 | -1,46 | 0,0462 |
| PRC1 | -6,3 | 0,5367 | PAIP2 | 1,39 | 0,0491 | NFYA | -2,55 | 0,0963 |
| PRDM1 | 2,1 | 0,7299 | PAK3 | -2,74 | 0,3025 | NHS | 1,23 | 0,0256 |
| PRDM6 | -2,66 | 0,0333 | PALLD | -2,02 | 0,5455 | NHSL1 | -2,43 | 0,0655 |
| PRDM8 | -2,03 | 0,6851 | PALMD | -3,24 | 0,6622 | NID1 | -2,06 | 0,136 |
| PRDX1 | 2,22 | 0,1878 | PANK1 | 2,51 | 0,1372 | NIP7 | -2 | 0,0387 |
| PREPL | 1,71 | 0,0318 | PAPLN | -1,7 | 0,0168 | NIPAL1 | -2,38 | 0,1779 |
| PRG4 | -7,07 | 0,2277 | PAPPA2 | -2,28 | 0,0684 | NIPAL2 | 2,39 | 0,6193 |
| PRIMPOL | -3,05 | 0,0190 | PARD6G-AS1 | 1,26 | 0,0402 | NLGN4Y | 3,29 | 0,8209 |
| PRKAA2 | 2,52 | 0,0178 | PARP1 | 2,53 | 0,0607 | NLGN4Y-AS1 | 2,05 | 0,0868 |
| PRKAG2 | 2,56 | 0,0136 | PARP11 | -1,71 | 0,0016 | NME5 | 3,39 | 0,0081 |
| PRKCA | 2,97 | 0,1619 | PARP4 | 2,06 | 0,1152 | NME6 | -1,77 | 0,0203 |
| PRKCH | -3,12 | 0,0444 | PARP9 | 4,12 | 0,1665 | NOC4L | -2,17 | 0,0879 |
| PRKD3 | 2,33 | 0,2658 | PARPBP | 2,21 | 0,0247 | NOL4L | -1,77 | 0,0134 |
| PRKG2 | -1,43 | 0,0451 | PATL1 | 2,88 | 0,0940 | NOTCH2 | 2,57 | 0,0016 |
| PRKRIRP9 | -1,78 | 0,0137 | PATZ1 | 2,13 | 0,2971 | NOTCH2NL | 2,22 | 0,0007 |
| PRPF39 | 2,14 | 0,2649 | PAXBP1 | 2,11 | 0,3366 | NPIPA1 | -2,02 | 0,3093 |
| PRPSAP2 | 2,13 | 0,1282 | PCED1A | 2,45 | 0,1313 | NPR2 | 1,83 | 0,0254 |
| PRR11 | -2,7 | 0,8124 | PCMTD2 | 3,29 | 0,0680 | NPR3 | -2,36 | 0,6967 |
| PRR14L | -2,12 | 0,6809 | PCP4 | -1,41 | 0,0423 | NPTN | 2,46 | 0,0087 |
| PRR16 | 1,79 | 0,0149 | PDCL | 2,04 | 0,0314 | NQO1 | -1,52 | 0,0236 |
| PRRC2A | -2,24 | 0,9141 | PDCL3 | 2,01 | 0,3989 | NR2F1 | -2,61 | 0,0271 |
| PRSS12 | 4,45 | 0,2336 | PDE1C | 4,78 | 0,7953 | NR4A1 | -7,66 | 0,6392 |
| PRTG | 1,41 | 0,0331 | PDE4B | 2,73 | 0,2453 | NR4A2 | -2,22 | 0,931 |
| PRUNE2 | 3,43 | 0,2971 | PDGFRA | 3,78 | 0,6914 | NR4A3 | -3,39 | 0,6375 |
| PSAT1 | -2,32 | 0,3540 | PDGFRB | 8,2 | 0,5109 | NRAS | -1,98 | 0,0132 |
| PSAT1P3 | -1,86 | 0,0298 | PDHX | -2,22 | 0,0049 | NRCAM | 2,37 | 0,6306 |
| PSD3 | 2,23 | 0,0041 | PDIA5 | -2,28 | 0,5434 | NRG1 | -2,18 | 0,4305 |
| PSMB3 | 2,01 | 0,1702 | PDP1 | 2,28 | 0,0938 | NRM | -3,9 | 0,3031 |
| PSMB8 | 2,95 | 0,0825 | PDP2 | 1,95 | 0,0026 | NRN1 | -2,03 | 0,3657 |
| PSMB9 | 3,71 | 0,1281 | PDPN | 3,15 | 0,2042 | NRP2 | 2,8 | 0,5624 |
| PSMD10 | 1,22 | 0,0263 | PDS5B | 1,7 | 0,0361 | NRXN3 | 3,58 | 0,4017 |
| PSPH | -1,34 | 0,0112 | PDZD11 | -2,06 | 0,3036 | NSUN4 | -2 | 0,037 |
| PTBP1 | -2,29 | 0,7835 | PDZD9 | -1,69 | 0,0139 | NTF3 | 5,07 | 0,0084 |
| PTCD2 | 2,08 | 0,2625 | PER3 | 2,54 | 0,5586 | NTRK3 | -2,9 | 0,4412 |
| PTCHD4 | 1,48 | 0,0318 | PERP | 3,46 | 0,1665 | NUAK1 | -2,74 | 0,8693 |
| PTGER4 | 2,34 | 0,4804 | PFKFB4 | 2,21 | 0,8565 | NUBPL | 1,64 | 0,0349 |
| PTGFR | -2,98 | 0,0254 | PGAM5 | 2,03 | 0,0977 | NUDC | -3,26 | 0,0287 |
| PTGR1 | 1,51 | 0,0224 | PGF | 4,29 | 0,9388 | NUDCD1 | -3,29 | 0,026 |
| PTHLH | -10,22 | 0,2443 | PGRMC1 | 1,68 | 0,0369 | NUDCD3 | -2,2 | 0,03 |
| PTN | -2,04 | 0,4067 | PGS1 | -1,31 | 0,0261 | NUF2 | -4,11 | 0,417 |
| PTPN11 | 1,33 | 0,0060 | PHF10 | 3,01 | 0,0312 | NUP107 | -2,39 | 0,0179 |
| PTPN20CP | 1,55 | 0,0083 | PHF7 | 2,67 | 0,0040 | NUP188 | -2,36 | 0,0098 |
| PTPRD | -2,18 | 0,3485 | PHLPP2 | 2,81 | 0,0397 | NUP50 | -1,72 | 0,0302 |
| PTPRK | 1,36 | 0,0211 | PI15 | -2,52 | 0,1212 | NUP54 | -2,18 | 0,1747 |
| PTPRN | -1,99 | 0,0018 | PI16 | -2,89 | 0,7585 | NUP62 | -2,11 | 0,1522 |
| PTPRU | -2,57 | 0,4198 | PI3 | -1,41 | 0,0152 | NUP85 | -4,34 | 0,0665 |
| PVRL2 | 2,27 | 0,6027 | PIGA | -2,12 | 0,7131 | NUSAP1 | -9,47 | 0,1861 |
| PVT1 | 2,47 | 0,2658 | PIGM | 1,46 | 0,0135 | NXF1 | -2,17 | 0,0127 |
| PXDN | 2,12 | 0,5352 | PIGX | -1,87 | 0,0374 | NXPE3 | 1,28 | 0,0392 |
| PXK | -1,65 | 0,0266 | PIK3R3 | 1,57 | 0,0215 | OAF | 2,47 | 0,7793 |
| PXYLP1 | 2,38 | 0,3067 | PIM2 | 2,08 | 0,0767 | OAT | 1,82 | 0,0148 |
| PYCR2 | -3,99 | 0,8042 | PIP5K1C | -2,19 | 0,5834 | OCIAD2 | 4,42 | 0,2967 |
| PYGO2 | -2,19 | 0,0567 | PIR | 2,14 | 0,5167 | OCLN | -2,72 | 0,1594 |
| PYHIN1 | -1,41 | 0,0380 | PKMYT1 | 2,2 | 0,1591 | OIP5 | -2,87 | 0,619 |
| QTRT1 | 1,64 | 0,0492 | PKP2 | -4,51 | 0,2552 | OLFML3 | -3,28 | 0,8064 |
| RAB11A | 1,51 | 0,0344 | PKP4 | 2,27 | 0,0362 | OPA1 | 1,57 | 0,0188 |
| RAB13 | 2,06 | 0,2923 | PLA2G12A | -2,75 | 0,0420 | OR1L8 | -1,43 | 0,0078 |
| RAB20 | 1,4 | 0,0102 | PLA2G4A | 2,55 | 0,7911 | OR2AK2 | -1,55 | 0,0293 |
| RAB29 | 2,06 | 0,1799 | PLAG1 | -2,67 | 0,0439 | OR2D3 | 1,28 | 0,0393 |
| RAB30 | 2,29 | 0,0441 | PLAT | 2,13 | 0,5540 | OR52A1 | -1,25 | 0,0206 |
| RAB3B | 2,61 | 0,2051 | PLBD2 | -2,14 | 0,6702 | OR52K2 | 1,29 | 0,0306 |
| RABL3 | 2,02 | 0,0033 | PLCE1 | -2,05 | 0,0279 | OR6T1 | -1,31 | 0,0183 |
| RAC2 | 2,06 | 0,5644 | PLCZ1 | 1,69 | 0,0081 | OR6X1 | -1,37 | 0,0327 |
| RAD23A | -2,29 | 0,5217 | PLD1 | -2,37 | 0,3144 | OR8B12 | 1,47 | 0,0318 |
| RAD51AP1 | -3,66 | 0,2950 | PLD3 | -2,9 | 0,6011 | OR8G2 | -1,34 | 0,0334 |
| RAD54B | -3,04 | 0,3302 | PLEKHA2 | 2,94 | 0,2957 | ORC4 | -1,62 | 0,032 |
| RALA | -2,24 | 0,2496 | PLEKHA6 | 1,59 | 0,0493 | ORC5 | -2,13 | 0,1691 |
| RALGDS | -2,04 | 0,6652 | PLEKHG1 | 2,22 | 0,6733 | ORC6 | -2,42 | 0,2741 |
| RALGPS2 | 2,17 | 0,7209 | PLEKHO1 | 2,99 | 0,4003 | ORMDL3 | 3,29 | 0,58 |
| RANBP10 | 1,58 | 0,0242 | PLGLB1 | 1,37 | 0,0206 | OSGEP | -2,51 | 0,1638 |
| RAP1GAP2 | -3,43 | 0,1721 | PLIN2 | -3,15 | 0,7772 | OSR2 | -5,55 | 0,547 |
| RARG | -2,62 | 0,9182 | PLK1 | 4,5 | 0,4103 | OST4 | 1,26 | 0,027 |
| RASA4 | -2,42 | 0,3721 | PLOD1 | -2,9 | 0,8965 | OSTC | 1,25 | 0,0308 |
| RASA4B | -4,82 | 0,2436 | PLOD2 | -3,3 | 0,6233 | OSTM1 | 2,14 | 0,0019 |
| RBBP8 | -2,66 | 0,1715 | PLPP3 | 2,25 | 0,0854 | OTUD4 | 2,85 | 0,2717 |
| RBM24 | 2,18 | 0,1279 | PLPP4 | -2,07 | 0,8208 | P3H2 | 33,49 | 0,0094 |
| RBM39 | -2,18 | 0,2984 | PLS1 | -1,66 | 0,0494 | P4HA1 | 2,02 | 0,1522 |
| RBM48 | -1,26 | 0,0313 | PLSCR4 | 3,38 | 0,0634 | PAG1 | 3,11 | 0,1818 |
| RBMY1A3P | 1,27 | 0,0475 | PLTP | 3,18 | 0,2984 | PAK1 | 2,91 | 0,1677 |
| RBP1 | -1,3 | 0,0465 | PLXDC2 | -2,28 | 0,1697 | PALLD | 7,11 | 0,0993 |
| RBP4 | -2,99 | 0,2397 | PLXNA2 | -4,23 | 0,7956 | PALMD | 30,56 | 0,1158 |
| RBPMS | 1,59 | 0,0493 | PLXNA3 | -2,26 | 0,7843 | PAMR1 | 3,97 | 0,9271 |
| RCAN2 | 1,95 | 0,0225 | PLXNA4 | 3,69 | 0,0568 | PAPD7 | -2,27 | 0,1252 |
| RCBTB1 | -1,91 | 0,0120 | PLXND1 | 2,11 | 0,3586 | PAPLN | 1,79 | 0,0347 |
| RCC2 | -2,82 | 0,7308 | PMCH | -2,31 | 0,2176 | PAPPA | 2,98 | 0,1181 |
| RDH5 | 3,26 | 0,0612 | PMP22 | -2,09 | 0,4133 | PAPSS2 | 1,51 | 0,0162 |
| RELB | 2,13 | 0,1984 | PMPCA | 1,58 | 0,0184 | PARP1 | -1,91 | 0,0486 |
| RGS2 | -11,24 | 0,2838 | PNRC2 | 2,17 | 0,0939 | PARP8 | 2,22 | 0,2101 |
| RGS5 | 51,81 | 0,3415 | PODN | 5,4 | 0,1002 | PARP9 | -2,65 | 0,1579 |
| RGS7 | 1,23 | 0,0439 | PODXL | -2,84 | 0,8078 | PARPBP | -1,81 | 0,0497 |
| RGS7BP | 6,93 | 0,0234 | POLA2 | 3,83 | 0,7003 | PATL1 | -2,02 | 0,1276 |
| RHOJ | -2,11 | 0,9343 | POLD1 | 2,62 | 0,0959 | PATZ1 | -1,56 | 0,0349 |
| RHOQ | 1,81 | 0,0126 | POLI | -2,31 | 0,4070 | PBK | -2,28 | 0,1658 |
| RHOQP3 | 1,67 | 0,0003 | POLK | 2,05 | 0,2050 | PBLD | 2,53 | 0,0076 |
| RIN3 | 3,45 | 0,1892 | POLR1D | -2,18 | 0,0138 | PCDH1 | -2,67 | 0,0822 |
| RMI1 | -2,39 | 0,1585 | POLR2D | 3,05 | 0,0718 | PCDH10 | -2,09 | 0,4934 |
| RMI2 | -2,44 | 0,2788 | POLR3C | 2,41 | 0,1900 | PCDH7 | -3,02 | 0,2372 |
| RN7SL1 | -2,71 | 0,8999 | POLR3G | -3,29 | 0,2543 | PCDHB13 | -1,49 | 0,0476 |
| RN7SL2 | -2,22 | 0,9802 | POPDC3 | -7,92 | 0,0742 | PCDHB9 | -1,33 | 0,0175 |
| RNA45S5 | -2,04 | 0,9688 | POSTN | -25,8 | 0,0840 | PCK2 | -3,64 | 0,2463 |
| RNA5-8S5 | -2,39 | 0,6594 | POTEB | -1,34 | 0,0387 | PCMTD2 | -2,04 | 0,007 |
| RNA5S1 | 2,08 | 0,2792 | POTEB3 | 1,76 | 0,0318 | PDE1C | 2,33 | 0,2863 |
| RNA5S10 | 2,08 | 0,2792 | PPCDC | -1,21 | 0,0335 | PDE3A | 2,11 | 0,647 |
| RNA5S11 | 2,08 | 0,2792 | PPIL6 | -2,64 | 0,0355 | PDE3B | -2,59 | 0,0318 |
| RNA5S17 | 2,08 | 0,2792 | PPIP5K1 | 1,75 | 0,0027 | PDE4B | -2,66 | 0,1536 |
| RNA5S2 | 2,08 | 0,2792 | PPM1K | -2,22 | 0,1347 | PDE5A | 5,26 | 0,0055 |
| RNA5S3 | 2,08 | 0,2792 | PPP1R14C | -2,09 | 0,0013 | PDGFC | 2,62 | 0,0567 |
| RNA5S4 | 2,08 | 0,2792 | PPP1R17 | -1,49 | 0,0238 | PDK1 | 3,53 | 0,0459 |
| RNA5S6 | 2,08 | 0,2792 | PPP2R2B | -2,28 | 0,0013 | PDK3 | -2,02 | 0,1482 |
| RNA5S7 | 2,08 | 0,2792 | PPT1 | 1,79 | 0,0322 | PDLIM1 | 3,26 | 0,7599 |
| RNASEH2A | 2,42 | 0,6305 | PQBP1 | 2,09 | 0,2117 | PDLIM2 | 6,95 | 0,3441 |
| RNASET2 | 2,41 | 0,1297 | PQLC2L | 2,96 | 0,5393 | PDP1 | -2,17 | 0,1606 |
| RNF115 | 2,13 | 0,0674 | PRDM1 | 2,17 | 0,4377 | PDPN | -2,16 | 0,3641 |
| RNF125 | -1,99 | 0,0003 | PRDM8 | -4,72 | 0,9791 | PDS5B | -2,1 | 0,0072 |
| RNF150 | 15,85 | 0,0582 | PRDX1 | 2,94 | 0,1370 | PDSS1 | -2,19 | 0,4416 |
| RNLS | 1,59 | 0,0437 | PRDX4 | -2,19 | 0,0021 | PDZD11 | 3,45 | 0,0695 |
| RNPC3 | -1,6 | 0,0385 | PRELP | 1,27 | 0,0368 | PEX2 | 1,41 | 0,0299 |
| RNPEPL1 | 2,24 | 0,5141 | PREX1 | -2,87 | 0,4285 | PFAS | -2,31 | 0,0071 |
| RNY4 | -1,65 | 0,0099 | PRG4 | -14,2 | 0,1270 | PFDN5 | 1,73 | 0,022 |
| RP11-504P2 | 1,62 | 0,0088 | PRKCA | 3,96 | 0,0555 | PGAM5 | -2,37 | 0,0532 |
| RPGR | -2,54 | 0,0122 | PRKD3 | 2,88 | 0,0429 | PGF | -2,31 | 0,3468 |
| RPS6KA1 | 1 | 0,0067 | PRMT3 | 1,51 | 0,0198 | PGM5-AS1 | 2,1 | 0,0546 |
| RPS6KA2 | 2,17 | 0,2584 | PRPF3 | 2,47 | 0,1651 | PHACTR2 | -1,44 | 0,0364 |
| RPS6KA5 | -2,79 | 0,0621 | PRPSAP1 | 2,31 | 0,0914 | PHF10 | -2,47 | 0,0088 |
| RRAS2 | 2,83 | 0,3522 | PRRG1 | -2 | 0,0457 | PHF19 | -2,48 | 0,0239 |
| RRM2B | 5,34 | 0,1212 | PRRX1 | -1,35 | 0,0246 | PHF3 | -2,17 | 0,0457 |
| RTKN2 | 3,39 | 0,4195 | PSG4 | -2,4 | 0,8010 | PHGDH | -4,01 | 0,3694 |
| RTTN | -3,03 | 0,0655 | PSG5 | -2,58 | 0,4247 | PIGF | 2 | 0,0271 |
| RUNX1T1 | -2,37 | 0,1210 | PSMB8 | 2,17 | 0,1471 | PIGV | 1,37 | 0,0171 |
| S100A2 | -2,54 | 0,1664 | PSMB9 | 4,19 | 0,1107 | PIGX | 1,79 | 0,034 |
| S1PR1 | -14,32 | 0,3566 | PSMD2 | 2,03 | 0,2893 | PINX1 | -2,44 | 0,1135 |
| SAA1 | -7,26 | 0,8013 | PSMG2 | -1,34 | 0,0475 | PIR | -2,59 | 0,2087 |
| SAAL1 | -2,37 | 0,4380 | PTGFR | -3,73 | 0,1227 | PKIA | -4,68 | 0,0294 |
| SACM1L | 2,11 | 0,0040 | PTHLH | -6,04 | 0,2249 | PKP4 | -2 | 0,1325 |
| SAE1 | 3,26 | 0,5486 | PTPRB | 2,23 | 0,5821 | PLAT | 7,71 | 0,8515 |
| SAMD1 | -2,0 | 0,1577 | PTPRN | -2,16 | 0,0007 | PLAU | 2,26 | 0,8604 |
| SAMD9 | -2,27 | 0,0285 | PTS | -2,78 | 0,0906 | PLCL2 | 1,41 | 0,0391 |
| SART3 | -1,63 | 0,0187 | PVRL1 | 2,72 | 0,5650 | PLCZ1 | -1,57 | 0,0465 |
| SASS6 | -3,28 | 0,2026 | PVRL2 | 2,02 | 0,1764 | PLD1 | 1,65 | 0,0216 |
| SBSN | -2,3 | 0,9890 | PVRL3 | 2,27 | 0,2395 | PLD2 | -2,01 | 0,0163 |
| SC5D | 2,06 | 0,0357 | PVT1 | 5,59 | 0,0626 | PLD3 | 2,85 | 0,9445 |
| SCFD2 | 2,4 | 0,2693 | PXN | 3,02 | 0,4246 | PLEC | -2,25 | 0,4954 |
| SCN1A | 2,22 | 0,0295 | PYCR2 | -2,42 | 0,6673 | PLEKHA8 | 1,71 | 0,0283 |
| SCN3A | 6,07 | 0,0187 | PYROXD2 | -1,6 | 0,0377 | PLEKHF1 | -1,35 | 0,0259 |
| SCN9A | 2,82 | 0,6511 | QKI | 2,48 | 0,0444 | PLEKHG1 | -2,4 | 0,0158 |
| SCP2 | 2,18 | 0,2480 | QRICH1 | 1,78 | 0,0440 | PLEKHG5 | -2,44 | 0,068 |
| SCRN1 | 5,33 | 0,2762 | RAB27B | -2,89 | 0,2244 | PLG | -1,52 | 0,0319 |
| SCUBE2 | 1,33 | 0,0383 | RAB38 | 2,3 | 0,1279 | PLIN2 | 2,29 | 0,5643 |
| SCUBE3 | 27,49 | 0,1194 | RAB3B | 6,28 | 0,0497 | PLK1 | -4,26 | 0,2571 |
| SCYL2 | 1,59 | 0,0228 | RAB3IP | 2,55 | 0,2806 | PLK4 | -3,3 | 0,3452 |
| SDC4 | 2,08 | 0,1407 | RAC2 | 6,06 | 0,4435 | PLOD1 | 2,6 | 0,5308 |
| SDCBP | 2,1 | 0,1807 | RACGAP1 | 2,21 | 0,4635 | PLOD2 | 9,35 | 0,0445 |
| SDF2L1 | -2,05 | 0,9717 | RAD51B | -1,76 | 0,0458 | PLPP4 | 2,75 | 0,3576 |
| SDR42E1 | 1,39 | 0,0129 | RAD9A | 1,49 | 0,0361 | PLPPR2 | 3,12 | 0,8417 |
| SEC14L1 | -2,06 | 0,0228 | RAI14 | 2,11 | 0,1316 | PLPPR4 | 47,3 | 0,0673 |
| SEC14L5 | -1,31 | 0,0307 | RALGAPA2 | 2,69 | 0,1322 | PMF1 | -2,12 | 0,0113 |
| SEC14L6 | 1,3 | 0,0480 | RALGPS2 | 3,8 | 0,2339 | PMPCA | -1,42 | 0,0363 |
| SEC16B | -1,87 | 0,0447 | RANBP10 | 1,45 | 0,0284 | PNP | -4,11 | 0,1258 |
| SEC22B | 2,52 | 0,0119 | RANBP6 | -1,9 | 0,0226 | PODN | -2,64 | 0,3541 |
| SEL1L3 | -4,32 | 0,2503 | RAPGEF5 | -2,67 | 0,7554 | PODXL | -13,46 | 0,3168 |
| SELPLG | 2,4 | 0,1338 | RARG | -2,33 | 0,9884 | POLA1 | -2,72 | 0,1424 |
| SEMA6A | -1,47 | 0,0359 | RASA4B | -4,29 | 0,3932 | POLA2 | -5,42 | 0,5937 |
| SEMA6A-AS1 | 1,89 | 0,0027 | RASGRP3 | 2,71 | 0,8171 | POLD1 | -3,07 | 0,0429 |
| SEPTIN8 | -2,52 | 0,7473 | RASSF2 | 2,61 | 0,9357 | POLE | -2,23 | 0,0617 |
| SERBP1P4 | -1,43 | 0,0200 | RASSF9 | -2,39 | 0,0101 | POLG2 | -1,71 | 0,0239 |
| SERINC2 | 3,35 | 0,1587 | RAVER2 | 2,03 | 0,0729 | POLR2J3 | 1,93 | 0,0429 |
| SERPINA9 | 4,74 | 0,0443 | RBBP8 | -2,14 | 0,1299 | POLR3B | -2,21 | 0,0438 |
| SERPINB1 | 1,46 | 0,0172 | RBM12 | 3,6 | 0,0494 | POMZP3 | -1,74 | 0,0036 |
| SERPINF1 | 2,13 | 0,5955 | RBM18 | -1,6 | 0,0470 | POSTN | 41,41 | 0,0482 |
| SERPINH1 | -3,17 | 0,8463 | RBM6 | 2,2 | 0,1360 | POT1 | -2,31 | 0,5054 |
| SERTAD3 | -3,2 | 0,4679 | RBM7 | -1,54 | 0,0163 | PPM1D | -2,26 | 0,0754 |
| SF3A2 | -2,3 | 0,8303 | RBMX | 1,58 | 0,0354 | PPM1K | 2,04 | 0,0021 |
| SFTA3 | -1,37 | 0,0378 | RBP4 | -3,17 | 0,1474 | PPP1R14A | 2,17 | 0,0279 |
| SFXN4 | 1,24 | 0,0436 | RCBTB1 | -2,22 | 0,0020 | PPP1R16B | -1,15 | 0,0411 |
| SGCD | -4,3 | 0,0685 | RCC1 | 2,18 | 0,2080 | PPP1R1A | 1,21 | 0,0397 |
| SGCE | 2,08 | 0,0723 | RDH10 | 2,87 | 0,5283 | PPP2R2B | 2,15 | 0,027 |
| SGCG | -2,07 | 0,1275 | RECQL | 3,95 | 0,0431 | PPP3CB-AS1 | -2,68 | 0,3483 |
| SGIP1 | 17,35 | 0,1314 | RECQL4 | 2,44 | 0,3047 | PPP6R3 | -2,55 | 0,0245 |
| SGK3 | 1,87 | 0,0024 | REEP5 | -1,81 | 0,0196 | PRC1 | -3,19 | 0,3211 |
| SGOL1 | -2,49 | 0,4066 | RELB | 2,66 | 0,4462 | PRDM15 | 1,38 | 0,0422 |
| SGOL2 | -5,24 | 0,3593 | RERE | -2,08 | 0,1341 | PRDM6 | -2,63 | 0,7644 |
| SH3BGR | -2,4 | 0,0397 | REV3L | 2,54 | 0,4913 | PRDM7 | 1,26 | 0,0419 |
| SHMT2 | -2,08 | 0,6839 | RFC2 | 3,08 | 0,2031 | PRDX4 | 1,74 | 0,022 |
| SHTN1 | -2,82 | 0,6267 | RFTN2 | -2,04 | 0,5461 | PREX1 | 3,01 | 0,9941 |
| SIAH1 | -3,72 | 0,1219 | RFWD3 | 3,62 | 0,1022 | PRG4 | 2,93 | 0,2148 |
| SIGLECL1 | -1,23 | 0,0325 | RGPD2 | -2,2 | 0,0833 | PRIMPOL | -1,52 | 0,0054 |
| SIK1 | -2,11 | 0,2340 | RGPD5 | 2,1 | 0,0449 | PRKAG2 | 2,25 | 0,0387 |
| SIM2 | 2,54 | 0,2581 | RGS2 | -2,56 | 0,3161 | PRKD2 | -1,63 | 0,0104 |
| SIRPA | -2,14 | 0,4931 | RGS5 | 6,85 | 0,0587 | PRKG1 | -2,06 | 0,1829 |
| SKA1 | -2,46 | 0,3511 | RGS7BP | 4,69 | 0,0385 | PRPF3 | -2,26 | 0,0398 |
| SKA3 | -2,88 | 0,1851 | RGS8 | -1,36 | 0,0325 | PRPSAP1 | -2,13 | 0,1699 |
| SKAP1 | -1,33 | 0,0204 | RHAG | -1,39 | 0,0105 | PRR11 | -2,32 | 0,8517 |
| SLC12A6 | -2,23 | 0,0006 | RIBC2 | 2,29 | 0,0342 | PRR14 | -1,37 | 0,0295 |
| SLC15A3 | 2,1 | 0,7607 | RIC8A | 2,36 | 0,2475 | PRR14L | -2,66 | 0,0596 |
| SLC17A5 | 1,81 | 0,0462 | RIIAD1 | -1,57 | 0,0198 | PRRC1 | 1,43 | 0,0078 |
| SLC19A1 | -3,11 | 0,9147 | RIMBP3 | -1,5 | 0,0369 | PRSS12 | 2,56 | 0,381 |
| SLC1A1 | 3,28 | 0,0528 | RIPK4 | -1,67 | 0,0438 | PRTG | 1,23 | 0,0478 |
| SLC1A5 | -7,13 | 0,4118 | RMI2 | -2,23 | 0,5340 | PRUNE2 | 5,24 | 0,1115 |
| SLC20A2 | 2,06 | 0,1363 | RNA45S5 | -2,35 | 0,9909 | PSAT1 | -7,41 | 0,2454 |
| SLC22A18 | 1,34 | 0,0381 | RNASEH2A | 8,32 | 0,1512 | PSG5 | 4,02 | 0,2462 |
| SLC22A3 | -2,53 | 0,1742 | RNF122 | 2,24 | 0,1343 | PSG9 | 2,04 | 0,2631 |
| SLC25A4 | 1,82 | 0,0418 | RNF7 | -1,6 | 0,0157 | PSMD1 | 1,33 | 0,0447 |
| SLC26A2 | 2,71 | 0,0298 | ROR2 | 4,22 | 0,1123 | PSRC1 | -2,03 | 0,1723 |
| SLC2A5 | 2,04 | 0,0405 | RPA1 | 3,85 | 0,3534 | PTBP1 | -2,78 | 0,1186 |
| SLC2A9 | -1,72 | 0,0107 | RPEL1 | -1,49 | 0,0156 | PTGIS | -3,39 | 0,5277 |
| SLC34A1 | -1,32 | 0,0149 | RPF2 | -1,3 | 0,0466 | PTGS1 | -2,64 | 0,2153 |
| SLC38A1 | -3,5 | 0,0643 | RPGRIP1L | -2,15 | 0,0499 | PTHLH | -2,44 | 0,7441 |
| SLC38A5 | -2,8 | 0,4054 | RPRD1B | 2,23 | 0,1988 | PTPN11 | 1,27 | 0,0144 |
| SLC3A2 | -5,0 | 0,7646 | RPS6KA6 | -1,51 | 0,0400 | PTPRB | -2,96 | 0,3159 |
| SLC40A1 | 2,47 | 0,1978 | RRAS2 | 2,1 | 0,6953 | PTPRD | -2,48 | 0,1701 |
| SLC46A3 | 2,08 | 0,3105 | RRM2B | 2,23 | 0,3203 | PTPRU | -6,03 | 0,0644 |
| SLC47A2 | 1,29 | 0,0462 | RTKN2 | 2,49 | 0,6234 | PTRHD1 | 1,41 | 0,0375 |
| SLC4A4 | 2,78 | 0,2068 | RTN4R | 1,65 | 0,0415 | PTS | 2,18 | 0,1009 |
| SLC6A6 | 6,57 | 0,1158 | RTTN | -4,07 | 0,0162 | PUS7 | -2,22 | 0,0891 |
| SLC6A9 | -2,76 | 0,7981 | RUNX1 | 2,47 | 0,9449 | PVRIG | -1,22 | 0,0337 |
| SLC7A14 | 2,93 | 0,0264 | RUVBL1 | 2,3 | 0,2668 | PVRL1 | -3,44 | 0,0562 |
| SLC7A2 | 3,65 | 0,2634 | RUVBL2 | 2,01 | 0,2355 | PWP2 | -2,5 | 0,027 |
| SLC7A5 | -6,29 | 0,7258 | RWDD2A | -2,68 | 0,0515 | PXN | -3,58 | 0,2317 |
| SLMAP | 2,55 | 0,2097 | RWDD3 | -2,76 | 0,0234 | PXYLP1 | 4,07 | 0,0231 |
| SLN | -4,22 | 0,0127 | S100A13 | -2,07 | 0,0184 | QKI | -2,27 | 0,0296 |
| SLPI | -1,48 | 0,0100 | S100A2 | -2,22 | 0,1327 | QTRTD1 | -1,4 | 0,0073 |
| SMAD7 | -3,17 | 0,8251 | S100A3 | -2,5 | 0,0136 | RAB11FIP1 | -2,23 | 0,2577 |
| SMAD9 | -3,99 | 0,0532 | S100A4 | -2,97 | 0,1048 | RAB15 | -1,84 | 0,0091 |
| SMAGP | -1,48 | 0,0126 | SAE1 | 4,81 | 0,0768 | RAB27B | 2,57 | 0,1668 |
| SMAP2 | 2,3 | 0,1614 | SAMD9 | -3,6 | 0,1269 | RAB35 | -2,38 | 0,0666 |
| SMC4 | -2,58 | 0,3819 | SAMD9L | -1,96 | 0,0368 | RAB38 | -2,18 | 0,0037 |
| SMG6 | -2,16 | 0,9304 | SAP130 | 2,5 | 0,1419 | RAB3IP | -2,29 | 0,1399 |
| SMIM22 | -1,56 | 0,0109 | SART3 | -1,49 | 0,0486 | RABGAP1L | 1,2 | 0,0418 |
| SMIM7 | 2,64 | 0,2762 | SAT1 | -4,07 | 0,8915 | RABGGTA | 2,35 | 0,1109 |
| SMPDL3B | 1,39 | 0,0489 | SBNO1 | -1,48 | 0,0471 | RABL3 | 1,24 | 0,0123 |
| SMURF2 | 2,67 | 0,4105 | SBSN | -2,26 | 0,8015 | RACGAP1 | -2,71 | 0,172 |
| SNAI2 | 2,49 | 0,7228 | SCAPER | 1,51 | 0,0033 | RAD51 | -2,47 | 0,431 |
| SNRK | -2,69 | 0,1874 | SCARA3 | 2,7 | 0,0274 | RAD54B | -2,67 | 0,136 |
| SNRPA1 | -2,1 | 0,7369 | SCARNA18 | -1,45 | 0,0375 | RAF1 | -2,3 | 0,0743 |
| SNTB1 | 4,41 | 0,0994 | SCARNA2 | 2,19 | 0,0774 | RALGAPA2 | -2,27 | 0,0892 |
| SNX16 | 3,03 | 0,1044 | SCD | 3,94 | 0,1321 | RALGDS | -2,05 | 0,0465 |
| SNX25 | 1,56 | 0,0309 | SCFD2 | 2,65 | 0,3335 | RANBP17 | 2,23 | 0,3265 |
| SOCS2 | -2,06 | 0,6544 | SCG2 | -1,63 | 0,0302 | RANGAP1 | -2,3 | 0,1001 |
| SOCS5 | 2,11 | 0,0434 | SCN2B | -1,35 | 0,0185 | RAP1GAP2 | -6,18 | 0,0016 |
| SOCS7 | -2,15 | 0,5627 | SCPEP1 | 2,32 | 0,0707 | RAP2A | 2,06 | 0,2727 |
| SOD2 | 3,06 | 0,2115 | SCRG1 | -2,51 | 0,0683 | RAP2B | -1,78 | 0,0256 |
| SORBS2 | 6,17 | 0,8563 | SCRN1 | 10,07 | 0,3866 | RAPH1 | 2,05 | 0,0395 |
| SORT1 | 2,69 | 0,0573 | SCYL1 | -2,09 | 0,7272 | RARB | 4,08 | 0,9389 |
| SPACA7 | -1,65 | 0,0424 | SCYL2 | 1,36 | 0,0326 | RARRES1 | 2,59 | 0,1719 |
| SPANXN4 | 1,28 | 0,0371 | SDC1 | 2,32 | 0,1928 | RARS2 | 1,71 | 0,0439 |
| SPAST | -2,04 | 0,3416 | SDC2 | -3,78 | 0,0169 | RASA1 | 1,89 | 0,0384 |
| SPATA5L1 | -2,09 | 0,0073 | SEC11A | -1,6 | 0,0429 | RASGRP3 | -2,12 | 0,3623 |
| SPC25 | -5,87 | 0,6058 | SEC11C | -1,97 | 0,0300 | RBBP4 | -1,88 | 0,0141 |
| SPCS2 | -2,03 | 0,3348 | SEC14L1 | -2,72 | 0,6605 | RBM12 | -2,11 | 0,025 |
| SPDYA | -1,36 | 0,0438 | SEC24B | -1,42 | 0,0420 | RBM46 | 1,23 | 0,0419 |
| SPINK14 | -1,41 | 0,0282 | SEC61A2 | 3,77 | 0,1285 | RBP3 | -1,18 | 0,0416 |
| SPINT2 | -9,0 | 0,1845 | SELPLG | 2,13 | 0,2493 | RBPJ | 3,46 | 0,0267 |
| SPINT4 | 1,41 | 0,0460 | SEMA3D | -4,71 | 0,6251 | RCC1 | -2,5 | 0,0816 |
| SPOCD1 | 2,8 | 0,2264 | SENP5 | -1,32 | 0,0285 | RCC2 | -1,93 | 0,03 |
| SPRY4 | -2,52 | 0,8278 | SENP8 | 2,04 | 0,0539 | RDH10 | -3,22 | 0,4493 |
| SREBF2 | 2,44 | 0,2219 | SEPHS1 | 1,55 | 0,0253 | RDH5 | 2,29 | 0,1492 |
| SRGAP2C | -2,08 | 0,7817 | SERPINB2 | -3,57 | 0,8505 | RECQL | -2,1 | 0,1528 |
| SRGN | 2,45 | 0,1086 | SERPINB8 | 2,24 | 0,2558 | RECQL4 | -3,09 | 0,0694 |
| SRMS | 1,5 | 0,0151 | SERPINB9 | 5,4 | 0,1353 | RECQL5 | -1,56 | 0,0109 |
| SRPX | -2,78 | 0,2636 | SERPIND1 | 2,04 | 0,1388 | REEP5 | 2,78 | 0,0101 |
| SRPX2 | 3,05 | 0,1303 | SERPINF1 | 6,36 | 0,5983 | REXO2 | 1,29 | 0,0339 |
| SRSF12 | -1,31 | 0,0459 | SERPINH1 | -2,39 | 0,5023 | RFC2 | -4,91 | 0,0381 |
| SRSF7 | -2,24 | 0,8234 | SERPINI1 | -3,15 | 0,0385 | RFC4 | -2,07 | 0,4172 |
| SSXP9 | -1,55 | 0,0044 | SERTAD3 | -4,26 | 0,6929 | RFPL1 | 1,3 | 0,0329 |
| ST13P20 | 1,51 | 0,0180 | SERTAD4 | 1 | 0,0113 | RFTN2 | 2,62 | 0,3687 |
| ST3GAL5 | -3,13 | 0,4135 | SESN1 | 1,73 | 0,0324 | RFWD3 | -2,79 | 0,0407 |
| STAB1 | -1,85 | 0,0167 | SF3A3 | 2,89 | 0,0355 | RGN | 1,83 | 0,0265 |
| STAG3L4 | 2,49 | 0,0162 | SF3B6 | -2,06 | 0,2553 | RGPD1 | 1,46 | 0,0146 |
| STAMBPL1 | 2,19 | 0,4646 | SFPQ | 2,02 | 0,0901 | RGS2 | -3,14 | 0,7445 |
| STARD4 | 2,04 | 0,0038 | SFRP1 | -3,63 | 0,5601 | RGS5 | 7,57 | 0,8198 |
| STARD5 | 2,0 | 0,6400 | SFRP4 | 6,39 | 0,9566 | RGS7BP | 2,78 | 0,7855 |
| STARD7-AS1 | 2,18 | 0,2855 | SGCD | -7,43 | 0,0177 | RGS8 | 1,24 | 0,0476 |
| STAT1 | 2,35 | 0,2307 | SGCG | 3,55 | 0,6219 | RHOB | -1,59 | 0,0466 |
| STAT6 | 2,09 | 0,3334 | SGK1 | 6,66 | 0,2498 | RHOF | -1,23 | 0,0396 |
| STATH | -4,21 | 0,0955 | SGK3 | -2,37 | 0,8925 | RHOQ | 3,21 | 0,0074 |
| STAU2 | 1,62 | 0,0053 | SGMS1 | 2,3 | 0,0097 | RIBC2 | -1,67 | 0,0247 |
| STC2 | -2,08 | 0,7451 | SGOL2 | -3,16 | 0,6360 | RIIAD1 | 1,78 | 0,0072 |
| STIL | -2,44 | 0,2366 | SGSH | 1,32 | 0,0213 | RIMS1 | 2,04 | 0,2004 |
| STMN1 | -2,86 | 0,5529 | SGSM2 | 2,47 | 0,0940 | RIN3 | 2,31 | 0,214 |
| STRADA | 1,76 | 0,0496 | SH2B3 | 2,4 | 0,1639 | RMND5A | -1,73 | 0,0318 |
| STRADB | 1,88 | 0,0253 | SH2D4A | 2,1 | 0,6944 | RN7SL1 | -2,26 | 0,2219 |
| STT3B | 1,82 | 0,0025 | SH3BGRL3 | -1,31 | 0,0249 | RN7SL2 | -2,61 | 0,1916 |
| STXBP6 | -2,16 | 0,2464 | SHF | -1,66 | 0,0327 | RNA5S1 | 1,88 | 0,0278 |
| SUB1P4 | -1,54 | 0,0439 | SIK3 | -2,33 | 0,9957 | RNA5S10 | 1,88 | 0,0278 |
| SUCO | 2,28 | 0,2272 | SIX1 | -2,76 | 0,1804 | RNA5S11 | 1,88 | 0,0278 |
| SUFU | -2,15 | 0,1653 | SKAP1 | -1,29 | 0,0214 | RNA5S17 | 1,88 | 0,0278 |
| SULT1A4 | -2,15 | 0,7890 | SLC16A2 | 3,11 | 0,7415 | RNA5S2 | 1,88 | 0,0278 |
| SULT1B1 | -3,82 | 0,0802 | SLC22A3 | -2,36 | 0,1862 | RNA5S3 | 1,88 | 0,0278 |
| SULT1E1 | -9,24 | 0,1337 | SLC22A4 | 2,43 | 0,4589 | RNA5S4 | 1,88 | 0,0278 |
| SUOX | 2,98 | 0,4297 | SLC22A5 | 3,16 | 0,0784 | RNA5S6 | 1,88 | 0,0278 |
| SUPT7L | 1,94 | 0,0437 | SLC25A10 | 4,03 | 0,4469 | RNA5S7 | 1,88 | 0,0278 |
| SUSD1 | 2,32 | 0,1228 | SLC25A23 | 2,35 | 0,1972 | RNASE9 | 1,21 | 0,0399 |
| SUSD5 | 1,37 | 0,0285 | SLC25A26 | -3,03 | 0,6500 | RNASEH2A | -3,19 | 0,0643 |
| SUSD6 | 2,2 | 0,1490 | SLC25A27 | -2,28 | 0,1013 | RNASET2 | 2,77 | 0,0333 |
| SVEP1 | 2,04 | 0,1985 | SLC25A40 | 3,33 | 0,2119 | RNF122 | -2,22 | 0,1504 |
| SYNE1 | 1,72 | 0,0325 | SLC27A3 | 3,67 | 0,1751 | RNF125 | -1,51 | 0,0219 |
| SYNGAP1 | 2,34 | 0,3829 | SLC2A1 | -2,62 | 0,9412 | RNF135 | -1,12 | 0,0348 |
| SYS1 | 2,32 | 0,1030 | SLC2A14 | 2,26 | 0,3368 | RNF150 | 10,3 | 0,031 |
| SYT1 | -6,29 | 0,0902 | SLC2A3 | 2,47 | 0,3157 | RNF180 | 2,2 | 0,2946 |
| SYT11 | 2,77 | 0,1433 | SLC30A8 | -1,65 | 0,0446 | RNF4 | -2,05 | 0,2275 |
| SYT15 | -2,28 | 0,7263 | SLC35D1 | -1,54 | 0,0161 | RNF7 | 2,12 | 0,0509 |
| SYTL2 | 1,79 | 0,0220 | SLC39A10 | 2,18 | 0,0500 | RNPS1 | -2,52 | 0,127 |
| TAAR9 | 1,74 | 0,0391 | SLC39A8 | -6,65 | 0,2109 | RNU4ATAC | -2,05 | 0,3368 |
| TARDBPP1 | -1,64 | 0,0374 | SLC43A3 | 3,19 | 0,3031 | RNVU1-15 | 2,03 | 0,0307 |
| TATDN3 | -1,43 | 0,0346 | SLC44A2 | -2,79 | 0,9115 | RNY5 | 1,28 | 0,0462 |
| TBC1D9 | 2,6 | 0,1828 | SLC48A1 | 2,43 | 0,4282 | ROBO1 | 2,14 | 0,0041 |
| TBC1D9B | -3,25 | 0,8960 | SLC4A3 | 1,47 | 0,0227 | ROR2 | -4,03 | 0,1328 |
| TBRG4 | -2,28 | 0,8309 | SLC4A5 | -1,56 | 0,0142 | RP11-11N9.4 | 4,48 | 0,0376 |
| TBX3 | -2,61 | 0,3336 | SLC5A3 | -3,72 | 0,2259 | RP11-278J6.5 | 2,98 | 0,058 |
| TCAIM | 3,89 | 0,1454 | SLC6A6 | 6,39 | 0,0032 | RP11-349F21.5 | 2,11 | 0,656 |
| TCEA1 | -2,69 | 0,0017 | SLC7A11 | 6,11 | 0,0814 | RP11-44N21.3 | -2,47 | 0,0004 |
| TCEA3 | 2,86 | 0,6667 | SLC8B1 | 3 | 0,1115 | RPA1 | -2,95 | 0,306 |
| TCF21 | 2,14 | 0,0512 | SLC9A3R2 | -2 | 0,6417 | RPAP1 | -2,41 | 0,0707 |
| TCF4 | 2,04 | 0,5994 | SLN | -4,54 | 0,1969 | RPEL1 | 1,45 | 0,004 |
| TEAD2 | -2,12 | 0,2684 | SLTM | -2,39 | 0,0606 | RPGR | -3,46 | 0,0051 |
| TECPR1 | 1,38 | 0,0322 | SMAP1 | -2,2 | 0,0013 | RPGRIP1L | 2,28 | 0,0084 |
| TET3 | -2,03 | 0,4653 | SMAP2 | 2,16 | 0,2051 | RPRD1A | -1,61 | 0,0356 |
| TEX30 | -3,93 | 0,3038 | SMARCA1 | -2,05 | 0,0889 | RPS6KA5 | -4,27 | 0,0391 |
| TFCP2 | 2,24 | 0,3365 | SMC5 | -2,53 | 0,0856 | RRAS | 2,34 | 0,8224 |
| TFPI | 3,15 | 0,4951 | SMIM19 | -2,55 | 0,0841 | RRM2B | 2,12 | 0,4571 |
| TFPI2 | 3,29 | 0,1216 | SMIM4 | -2,16 | 0,1136 | RSPH3 | 1,44 | 0,0166 |
| TGFBR3 | 5,95 | 0,0178 | SMR3B | -1,41 | 0,0491 | RSPH9 | 1,63 | 0,0294 |
| TGIF1 | -2,42 | 0,9879 | SNAP23 | 2,09 | 0,2529 | RSPO2 | 1,53 | 0,0094 |
| TGM2 | 3,69 | 0,1907 | SNRPA | 2,68 | 0,0279 | RTTN | 2,18 | 0,9275 |
| THNSL2 | 4,39 | 0,0188 | SNX16 | 3,91 | 0,0003 | RUNX1T1 | -2,57 | 0,0268 |
| THSD4 | -2,06 | 0,3157 | SNX24 | -2,58 | 0,0162 | RWDD2A | 3,49 | 0,0699 |
| TIAM1 | -3,48 | 0,0443 | SNX4 | 1,83 | 0,0156 | S100A3 | 2,71 | 0,0478 |
| TIGAR | 1,71 | 0,0435 | SNX5 | 2,66 | 0,1840 | S100A4 | 2,17 | 0,2834 |
| TIMM21 | -2,15 | 0,0859 | SON | -1,93 | 0,0486 | S1PR1 | -15,79 | 0,0592 |
| TIMM9P2 | -1,34 | 0,0390 | SORBS2 | 3,35 | 0,6434 | SAA1 | -2,24 | 0,6362 |
| TINAGL1 | 3,02 | 0,0665 | SP3 | 2,01 | 0,0074 | SAAL1 | -2,15 | 0,3826 |
| TIPARP | 2,75 | 0,1088 | SPAG9 | 1,42 | 0,0349 | SAMD12 | 1,95 | 0,0302 |
| TIPIN | -3,69 | 0,3138 | SPATA6 | -1,71 | 0,0280 | SAR1A | 1,36 | 0,0103 |
| TLE4 | 1,85 | 0,0318 | SPC25 | -2,15 | 0,7048 | SAT1 | 3,02 | 0,7263 |
| TM4SF1 | 2,18 | 0,0340 | SPDYA | -1,95 | 0,0170 | SCAPER | -2,29 | 0,108 |
| TM9SF4 | 2,68 | 0,0125 | SPHK1 | 2,22 | 0,2489 | SCARA3 | -4,58 | 0,0451 |
| TMC3 | -4,7 | 0,7406 | SPINK1 | -2,58 | 0,0083 | SCARF1 | -1,08 | 0,0478 |
| TMCO4 | 1,7 | 0,0284 | SPINT2 | -10,1 | 0,1182 | SCARNA24 | -2,05 | 0,5621 |
| TMEM106A | 2,16 | 0,2790 | SPIRE1 | -1,37 | 0,0491 | SCD | -2,05 | 0,5076 |
| TMEM109 | -2,69 | 0,8864 | SPRY3 | 3,35 | 0,0456 | SCG5 | 2,71 | 0,0781 |
| TMEM117 | -2,19 | 0,0639 | SPRYD3 | -2,27 | 0,5829 | SCN1A | 1,99 | 0,0069 |
| TMEM133 | -2,86 | 0,1030 | SPRYD4 | 2,02 | 0,0173 | SCN2A | 2,37 | 0,1634 |
| TMEM135 | -1,64 | 0,0181 | SRD5A3 | -1,48 | 0,0379 | SCN3A | 3,69 | 0,0186 |
| TMEM184B | -2,23 | 0,4372 | SREBF2 | 2,99 | 0,2498 | SCN9A | 2,81 | 0,0397 |
| TMEM206 | -2,21 | 0,2236 | SRRT | 2,58 | 0,0162 | SCPEP1 | -2,24 | 0,1525 |
| TMEM217 | 2,67 | 0,0407 | SSBP2 | 2,5 | 0,3166 | SCRG1 | 2,28 | 0,0876 |
| TMEM233 | -3,02 | 0,0327 | SSPN | -5,92 | 0,1062 | SCUBE3 | 51,99 | 0,0986 |
| TMEM255B | -3,86 | 0,0195 | ST3GAL5 | -4,13 | 0,5225 | SDC2 | 3,74 | 0,0118 |
| TMEM263 | 1,58 | 0,0430 | STAMBP | -2,18 | 0,0635 | SDPR | -1,23 | 0,0363 |
| TMEM50A | 1,71 | 0,0054 | STARD13 | 2,43 | 0,0417 | SEC22B | 2,62 | 0,0002 |
| TMEM54 | -2,36 | 0,6543 | STAT6 | 1,63 | 0,0362 | SEL1L3 | -3,63 | 0,0926 |
| TMEM87B | 1,62 | 0,0111 | STATH | -5,56 | 0,1618 | SEMA3A | 2,08 | 0,942 |
| TMEM9 | -2,14 | 0,9020 | STC1 | 3,17 | 0,7741 | SEMA3D | 6,46 | 0,1131 |
| TMEM98 | 2,15 | 0,5253 | STK26 | -2,37 | 0,0407 | SEMA6C | -2,33 | 0,0493 |
| TMPRSS11E | -1,84 | 0,0129 | STMN3 | 1,31 | 0,0359 | SEPTIN8 | -2,09 | 0,0753 |
| TNFAIP2 | 2,39 | 0,4036 | STOM | 2,13 | 0,0536 | SERINC2 | 2,48 | 0,2084 |
| TNFAIP6 | -2,14 | 0,1005 | STRADA | 2,01 | 0,0838 | SERPINA9 | 5,21 | 0,0253 |
| TNFRSF10C | 2,34 | 0,1374 | STRAP | -1,47 | 0,0288 | SERPINB2 | 2,58 | 0,7854 |
| TNFRSF11A | 2,05 | 0,5333 | STRIP2 | 2,01 | 0,0754 | SERPINB9 | -5,05 | 0,0393 |
| TNFRSF11B | 3,06 | 0,0779 | STX17 | -2,01 | 0,0708 | SERPIND1 | -2,01 | 0,0373 |
| TNFRSF14 | 1,28 | 0,0424 | STX3 | 3,06 | 0,3060 | SERPINF1 | -3,71 | 0,8392 |
| TNFRSF8 | 1,4 | 0,0387 | STX6 | 2,24 | 0,1255 | SERPINI1 | 3,02 | 0,0366 |
| TNPO1 | 1,52 | 0,0100 | STXBP6 | -3,52 | 0,0774 | SERTAD4 | 2,74 | 0,1416 |
| TOB1 | 2,0 | 0,1395 | SUCNR1 | -2,74 | 0,0604 | SESN2 | -2,09 | 0,0854 |
| TOMM40L | -1,51 | 0,0168 | SUDS3 | 1,52 | 0,0473 | SETSIP | -1,35 | 0,0379 |
| TOP2A | -4,45 | 0,7717 | SULT1E1 | -5,25 | 0,2092 | SF3A2 | -3,62 | 0,0082 |
| TOPAZ1 | -1,72 | 0,0432 | SUPT20H | 1,38 | 0,0427 | SFI1 | -1,34 | 0,032 |
| TP53I11 | 1,34 | 0,0257 | SUPT7L | 1,74 | 0,0489 | SFRP4 | -3,87 | 0,9014 |
| TP53I3 | 2,06 | 0,2044 | SUSD1 | 2,94 | 0,0989 | SGCG | -6,7 | 0,1219 |
| TP53INP1 | 4,07 | 0,4634 | SVEP1 | 2,21 | 0,6407 | SGIP1 | 12,49 | 0,3069 |
| TPD52 | 2,12 | 0,0385 | SWT1 | 1,49 | 0,0419 | SGK1 | -4,42 | 0,1359 |
| TPD52L1 | -3,42 | 0,4006 | SYCP3 | -1,54 | 0,0065 | SGMS1 | -1,86 | 0,0205 |
| TPM2 | 1,63 | 0,0439 | SYNC | 2,07 | 0,5568 | SGOL1 | -2,85 | 0,3258 |
| TRA2A | 1,64 | 0,0382 | SYT1 | -9,39 | 0,0515 | SH2B3 | -2,11 | 0,2636 |
| TRAJ16 | -1,39 | 0,0382 | TACC3 | 23,16 | 0,1767 | SH3BGRL | 1,52 | 0,044 |
| TRAJ59 | 2,07 | 0,7814 | TAGLN | -3,19 | 0,8691 | SH3D21 | 2,06 | 0,1733 |
| TRAP1 | -2,62 | 0,8972 | TANC1 | 2,31 | 0,3455 | SHPK | -1,51 | 0,0342 |
| TRAPPC12 | 2,02 | 0,1198 | TANC2 | -2,03 | 0,0901 | SHROOM3 | 1,32 | 0,0303 |
| TRAPPC2L | 1,42 | 0,0290 | TAPBP | -2,26 | 0,5625 | SIAH1 | -2,54 | 0,1065 |
| TRAV26-2 | -1,48 | 0,0197 | TARDBP | 1,7 | 0,0406 | SIL1 | 2,46 | 0,2935 |
| TRAV9-1 | -1,21 | 0,0480 | TBC1D15 | 2,12 | 0,0010 | SIM2 | 2,23 | 0,5293 |
| TREML2 | 1,3 | 0,0264 | TBC1D8 | 2,35 | 0,0744 | SIPA1L2 | -2,03 | 0,2392 |
| TRIB2 | 2,16 | 0,6526 | TBC1D9 | 2,32 | 0,1133 | SKA1 | -5,34 | 0,0848 |
| TRIM22 | 7,1 | 0,0457 | TBCK | 2,38 | 0,0068 | SKA3 | -2,08 | 0,2165 |
| TRIM37 | 2,07 | 0,0832 | TBRG1 | 1,49 | 0,0463 | SKP2 | -2,06 | 0,0694 |
| TRIM47 | 2,56 | 0,8466 | TBX2 | -2,22 | 0,2593 | SLC13A3 | 1,58 | 0,0135 |
| TRIM52 | 1,57 | 0,0236 | TCEAL8 | -1,93 | 0,0112 | SLC16A1 | -2,13 | 0,1725 |
| TRIOBP | 2,1 | 0,2651 | TCF19 | 2,59 | 0,1791 | SLC16A2 | -2,16 | 0,8844 |
| TRIP13 | 2,24 | 0,2990 | TCF4 | 2,05 | 0,2944 | SLC17A5 | 2,74 | 0,0348 |
| TRIP4 | -1,53 | 0,0484 | TCONS_l2_00020055 | 1,37 | 0,0088 | SLC18A3 | 1,2 | 0,0398 |
| TRMU | -2,26 | 0,9845 | TCP10 | -2,09 | 0,0092 | SLC19A1 | -4,05 | 0,1304 |
| TRPC1 | 2,14 | 0,0634 | TCP11L1 | -1,64 | 0,0021 | SLC1A1 | 2,11 | 0,1626 |
| TRPC4 | 6,66 | 0,0335 | TECR | 2,02 | 0,1230 | SLC1A5 | -4,19 | 0,1291 |
| TRPV2 | 50,76 | 0,0060 | TECTA | 1,73 | 0,0394 | SLC22A15 | -2,14 | 0,0389 |
| TSC2 | 2,68 | 0,7114 | TEFM | -1,4 | 0,0272 | SLC25A16 | 1,84 | 0,0008 |
| TSEN15 | -1,36 | 0,0355 | TEP1 | 1,66 | 0,0438 | SLC25A24 | -1,47 | 0,0497 |
| TSPAN14 | 2,12 | 0,7172 | TERF2IP | -2,01 | 0,0282 | SLC25A26 | 2,16 | 0,176 |
| TSPAN5 | 2,87 | 0,1339 | TEX35 | -1,45 | 0,0166 | SLC25A51 | -2,33 | 0,1424 |
| TSPAN8 | -8,12 | 0,0406 | TFAP2B | 1,33 | 0,0281 | SLC25A52 | 1,96 | 0,0235 |
| TSR2 | 2,17 | 0,4797 | TFDP1 | 3,24 | 0,2812 | SLC26A2 | 1,48 | 0,0065 |
| TSSC2 | 1,4 | 0,0386 | TGFB2 | -2,07 | 0,4352 | SLC27A3 | -2,17 | 0,0168 |
| TTBK2 | -2,72 | 0,8804 | TGFBI | -2,88 | 0,6214 | SLC2A13 | 2,61 | 0,2297 |
| TTC39B | 1,96 | 0,0088 | TGFBR3 | 2,63 | 0,0145 | SLC2A14 | -3,99 | 0,3167 |
| TTC7B | 2,53 | 0,2949 | TGIF2 | 5,22 | 0,6726 | SLC2A3 | -3,51 | 0,3814 |
| TTK | -3,03 | 0,4769 | TGM2 | 2,07 | 0,2769 | SLC30A8 | 1,25 | 0,037 |
| TTLL12 | -2,13 | 0,7884 | THAP6 | 2,37 | 0,3646 | SLC35D1 | 2,24 | 0,0019 |
| TTPA | 1,3 | 0,0204 | THSD1 | 2,58 | 0,4240 | SLC38A5 | -2,14 | 0,5426 |
| TTTY23 | -1,18 | 0,0421 | TIAM1 | -5,11 | 0,0666 | SLC39A10 | -1,63 | 0,0162 |
| TUBB6 | -2,76 | 0,8943 | TIMELESS | 2,04 | 0,3256 | SLC39A8 | 3,4 | 0,0864 |
| TULP2 | -1,36 | 0,0281 | TIMM23B | 2,06 | 0,0064 | SLC3A2 | -3,52 | 0,0321 |
| TVP23B | 1,79 | 0,0040 | TIPARP | 3,99 | 0,0827 | SLC40A1 | 2,28 | 0,5116 |
| TVP23C | 1,55 | 0,0053 | TIPIN | -3,06 | 0,3619 | SLC41A2 | 4,01 | 0,1832 |
| TWSG1 | 1,95 | 0,0019 | TIPRL | -1,47 | 0,0347 | SLC43A3 | -5,14 | 0,1912 |
| TXNP2 | 1,71 | 0,0124 | TK1 | 3,7 | 0,0613 | SLC44A1 | 1,39 | 0,036 |
| TXNRD1 | 2,9 | 0,0688 | TK2 | -2,84 | 0,0292 | SLC44A2 | 2,01 | 0,4729 |
| UACA | 4,48 | 0,0289 | TLCD1 | -2,13 | 0,6754 | SLC4A5 | 1,54 | 0,0373 |
| UBE2C | -7,39 | 0,5729 | TM4SF1 | 2,37 | 0,1709 | SLC5A3 | 2,89 | 0,221 |
| UBE2D2 | -2,1 | 0,2234 | TM7SF2 | 1,48 | 0,0315 | SLC6A11 | 1,32 | 0,0274 |
| UBE2D4 | -1,86 | 0,0376 | TMA16 | -1,62 | 0,0485 | SLC6A12 | -1,22 | 0,0235 |
| UBE2F | 1,2 | 0,0485 | TMC3 | -2,77 | 0,8120 | SLC6A2 | -1,39 | 0,0045 |
| UBP1 | 1,35 | 0,0364 | TMCO4 | 2,66 | 0,0446 | SLC6A3 | 1,3 | 0,0199 |
| UFM1 | 1,42 | 0,0303 | TMEM110-MUSTN1 | -1,32 | 0,0378 | SLC7A11 | -6,78 | 0,0028 |
| UGCG | 2,04 | 0,0712 | TMEM128 | -3,12 | 0,1318 | SLC7A11-AS1 | -2,5 | 0,2864 |
| UGDH | 1,99 | 0,0159 | TMEM133 | -3,18 | 0,0526 | SLC7A14 | 2,11 | 0,6184 |
| UHRF2 | 2,03 | 0,3814 | TMEM134 | -2,3 | 0,5539 | SLC7A2 | 2,37 | 0,7563 |
| UMAD1 | 2,35 | 0,0563 | TMEM136 | -1,55 | 0,0047 | SLC7A5 | -5,96 | 0,4368 |
| UNC5B | -2,51 | 0,1556 | TMEM154 | 3,23 | 0,1050 | SLC7A8 | 2,02 | 0,7802 |
| UNC93B1 | -2,04 | 0,4506 | TMEM171 | 2,99 | 0,4972 | SLC8A1 | 2,09 | 0,0505 |
| UPK1B | -2,35 | 0,1184 | TMEM173 | 2,34 | 0,0387 | SLC8B1 | -2,74 | 0,0034 |
| UQCC1 | 1,63 | 0,0294 | TMEM181 | -2,33 | 0,2709 | SLC9A6 | 2,3 | 0,432 |
| UROC1 | 1,21 | 0,0345 | TMEM184B | -2,8 | 0,7285 | SLCO3A1 | -2,44 | 0,6905 |
| USP21 | -2,07 | 0,4753 | TMEM19 | 3,41 | 0,1291 | SLFN11 | -2,26 | 0,0218 |
| USP25 | -1,89 | 0,0438 | TMEM233 | -4,03 | 0,0481 | SLX4IP | -2,06 | 0,9539 |
| USP9Y | -3,12 | 0,5952 | TMEM243 | -1,84 | 0,0108 | SMAD6 | -2 | 0,2703 |
| USP9YP21 | 1,47 | 0,0226 | TMEM25 | -2,4 | 0,6424 | SMAD7 | -2,26 | 0,1952 |
| UTP20 | 2,34 | 0,5033 | TMEM263 | -2,09 | 0,1832 | SMAD9 | -1,74 | 0,0038 |
| UTP3 | 1,61 | 0,0174 | TMEM42 | 1 | 0,0174 | SMAP1 | 1,88 | 0,0063 |
| UXS1 | 2,78 | 0,1401 | TMEM45A | -2 | 0,2019 | SMC3 | -2,34 | 0,3455 |
| VAMP1 | 5,55 | 0,0626 | TMEM59 | -1,82 | 0,0315 | SMC4 | -4,01 | 0,7069 |
| VDR | 3,36 | 0,0659 | TMEM68 | 2,76 | 0,1620 | SMIM14 | 1,94 | 0,0113 |
| VEGFC | 2,89 | 0,1375 | TMF1 | -2,65 | 0,0055 | SMIM19 | 2,34 | 0,0952 |
| VGLL3 | 5,82 | 0,0874 | TMPO | 3,49 | 0,1095 | SMIM22 | -1,33 | 0,0183 |
| VIT | -3,32 | 0,3935 | TMTC1 | -3,62 | 0,5435 | SMIM4 | 2,61 | 0,0625 |
| VKORC1L1 | 1,4 | 0,0218 | TMTC2 | -2,18 | 0,2531 | SNORA19 | -2,11 | 0,485 |
| VLDLR | -2,4 | 0,4835 | TNC | -3,11 | 0,7205 | SNORA24 | 2,45 | 0,0154 |
| VPS13B | 2,45 | 0,3314 | TNFAIP2 | 2,27 | 0,7817 | SNORA25 | -2,2 | 0,1524 |
| VPS41 | 2,55 | 0,1850 | TNFAIP3 | -2,65 | 0,4096 | SNORA40 | 2,4 | 0,1557 |
| VRK1 | -3,27 | 0,2346 | TNFRSF1A | 2,13 | 0,1215 | SNORA5A | -3 | 0,0368 |
| WARS | -2,83 | 0,5932 | TNFSF13B | -2,22 | 0,0363 | SNORA70 | -2,03 | 0,0446 |
| WARSP1 | -1,94 | 0,0370 | TNFSF4 | -2,78 | 0,3770 | SNORA71C | -2,61 | 0,0837 |
| WDPCP | 2,89 | 0,0141 | TOE1 | 2,41 | 0,2109 | SNORA75 | -2,43 | 0,002 |
| WDR19 | 2,89 | 0,0137 | TOMM7 | -2,23 | 0,0179 | SNORA81 | -3,22 | 0,0513 |
| WDR20 | 1,2 | 0,0337 | TOPAZ1 | -1,5 | 0,0048 | SNORA9 | -2,44 | 0,0431 |
| WDR33 | 2,54 | 0,4035 | TP53 | 3,22 | 0,4273 | SNORD104 | -2,83 | 0,0359 |
| WDR35 | -1,67 | 0,0089 | TP53INP1 | 2,53 | 0,2451 | SNORD113 | -2,22 | 0,842 |
| WDR73 | 2,31 | 0,2054 | TP73-AS1 | 1,92 | 0,0382 | SNORD113-4 | 2,92 | 0,1142 |
| WDR74 | -2,06 | 0,7348 | TPCN1 | -2,93 | 0,7751 | SNORD114-30 | 2,22 | 0,1095 |
| WLS | 2,69 | 0,7187 | TPM1 | -3,32 | 0,2006 | SNORD117 | -2,34 | 0,0551 |
| WNT5A | 6,3 | 0,2413 | TRAF3 | 1,76 | 0,0298 | SNORD123 | 3,62 | 0,0384 |
| WRNIP1 | -2,04 | 0,9185 | TRAF3IP1 | -2,06 | 0,0045 | SNORD15B | -2,26 | 0,0873 |
| WSB1 | -2,0 | 0,7379 | TRAIP | 1,62 | 0,0083 | SNORD20 | -2,89 | 0,0118 |
| WWTR1 | -2,18 | 0,1485 | TRANK1 | -1,71 | 0,0022 | SNORD22 | 2,43 | 0,6552 |
| XAGE2 | -1,36 | 0,0268 | TRBV6-8 | 1,43 | 0,0321 | SNORD41 | -2,32 | 0,3736 |
| XIAP | 2,14 | 0,4520 | TREM1 | -1,6 | 0,0150 | SNORD4A | -3,22 | 0,0216 |
| XPNPEP3 | -2,18 | 0,0731 | TRIM22 | 2,74 | 0,2328 | SNORD59A | -3,23 | 0,3421 |
| XPOT | -2,92 | 0,0204 | TRIM26 | 1,41 | 0,0346 | SNORD63 | -2,8 | 0,1401 |
| XRCC4 | -2,83 | 0,0413 | TRIM31 | -1,33 | 0,0353 | SNORD69 | -4,06 | 0,0055 |
| XYLT1 | -2,22 | 0,8277 | TRIM37 | 2,33 | 0,6355 | SNORD70 | -2,99 | 0,5326 |
| YPEL3 | 2,35 | 0,7333 | TRIM49B | -1,24 | 0,0499 | SNORD93 | -2,54 | 0,6378 |
| YPEL5 | 1,53 | 0,0323 | TRIM49C | -1,39 | 0,0328 | SNORD99 | -3,03 | 0,2138 |
| ZBTB10 | -2,29 | 0,1287 | TRIP13 | 3,6 | 0,2137 | snoU109 | 2 | 0,1071 |
| ZBTB7C | 1,28 | 0,0314 | TRIP4 | -2,17 | 0,0808 | SNRK | -1,86 | 0,0472 |
| ZC3H6 | -1,98 | 0,0066 | TRMT2B | 2,46 | 0,1611 | SNRNP40 | -2,56 | 0,0097 |
| ZCCHC8 | -2,01 | 0,0622 | TRPC7 | -1,37 | 0,0194 | SNRPA | -2,7 | 0,0212 |
| ZDHHC16 | 2,52 | 0,3785 | TRPV2 | -2 | 0,2332 | SNRPB | -2,93 | 0,018 |
| ZFAND4 | 2,85 | 0,0283 | TSC1 | 2,11 | 0,1498 | SNRPC | -1,41 | 0,0393 |
| ZFP64 | -2,3 | 0,9253 | TSEN2 | 2,26 | 0,2762 | SNTB1 | 5,13 | 0,1207 |
| ZKSCAN1 | 2,5 | 0,1078 | TSGA10 | -1,34 | 0,0242 | SNX13 | 1,58 | 0,016 |
| ZKSCAN7 | 1 | 0,0059 | TSPAN4 | 2,16 | 0,0816 | SOAT1 | -1,93 | 0,0077 |
| ZMIZ1 | -2,17 | 0,3326 | TSPAN6 | 2,06 | 0,1492 | SOCS5 | 3,68 | 0,0102 |
| ZNF106 | 2,26 | 0,0108 | TSPAN8 | -8,46 | 0,0063 | SOGA3 | 1,68 | 0,0183 |
| ZNF124 | 1,59 | 0,0444 | TSPO | -2,6 | 0,6621 | SORCS2 | -1,22 | 0,0406 |
| ZNF165 | -1,73 | 0,0250 | TSPYL4 | 2,01 | 0,0597 | SORT1 | 2,61 | 0,1423 |
| ZNF185 | 3,27 | 0,1640 | TSR2 | 2,09 | 0,2000 | SPAG17 | 1,56 | 0,0414 |
| ZNF25 | 4,93 | 0,0990 | TTC3 | -2,58 | 0,0571 | SPC25 | -2,51 | 0,2938 |
| ZNF284 | -2,43 | 0,1330 | TTC6 | 1,67 | 0,0031 | SPECC1 | -2,19 | 0,7633 |
| ZNF319 | -1,59 | 0,0473 | TTC9 | -1,76 | 0,0374 | SPIRE1 | 1,39 | 0,0295 |
| ZNF383 | -3,01 | 0,0576 | TTLL3 | 2,93 | 0,0682 | SPTA1 | 1,34 | 0,0252 |
| ZNF385B | 1,39 | 0,0446 | TTLL7 | -2,07 | 0,0213 | SPTLC2 | -1,56 | 0,0294 |
| ZNF385D | -4,09 | 0,2517 | TUBB4B | 3,87 | 0,2037 | SRC | -2,16 | 0,1135 |
| ZNF391 | -2,75 | 0,0238 | TUBG1 | 3,43 | 0,1290 | SRGAP2 | -2,27 | 0,0378 |
| ZNF404 | 1,41 | 0,0462 | TUFT1 | -2,49 | 0,7477 | SRGAP2B | -2,06 | 0,0524 |
| ZNF410 | -3,02 | 0,9833 | TUSC3 | -2,53 | 0,3800 | SRGAP2C | -2 | 0,0199 |
| ZNF423 | 2,68 | 0,3309 | TWSG1 | 2,08 | 0,0091 | SRGN | 2,35 | 0,3356 |
| ZNF438 | -1,81 | 0,0020 | TXNDC2 | 1,43 | 0,0402 | SRRT | -2,2 | 0,0123 |
| ZNF519P4 | 1,57 | 0,0169 | TXNIP | 3,05 | 0,2758 | SRSF11 | -1,28 | 0,0304 |
| ZNF561 | 1,45 | 0,0337 | TXNRD1 | 2,9 | 0,0907 | SRSF3 | -2,08 | 0,1787 |
| ZNF567 | 2,0 | 0,0239 | TYMS | 6 | 0,2362 | SRSF4 | -2,1 | 0,1273 |
| ZNF577 | -1,5 | 0,0423 | UBA6 | 2,19 | 0,0709 | SSBP2 | -2,21 | 0,789 |
| ZNF596 | -2,45 | 0,0305 | UBA7 | 2,15 | 0,2704 | SSBP4 | -2,55 | 0,0161 |
| ZNF606 | 1,23 | 0,0416 | UBE2I | -1,52 | 0,0137 | SSPN | 6,37 | 0,1545 |
| ZNF608 | 1,5 | 0,0163 | UBE3B | -2,11 | 0,2785 | SSRP1 | -2,15 | 0,0605 |
| ZNF626 | 1,46 | 0,0362 | UGDH | 1,7 | 0,0324 | SSX3 | -1,56 | 0,0127 |
| ZNF670 | 3,15 | 0,1117 | UHRF1 | 4,64 | 0,2661 | ST3GAL6 | 2,44 | 0,4476 |
| ZNF680 | -2,18 | 0,0969 | ULK3 | 2,29 | 0,1651 | ST6GALNAC2 | 1,26 | 0,0307 |
| ZNF717 | 2,36 | 0,0920 | UMAD1 | 2,09 | 0,2291 | ST6GALNAC3 | -2,33 | 0,0524 |
| ZNF732 | 3,11 | 0,1557 | UNC13C | -1,25 | 0,0482 | STAMBPL1 | 3,66 | 0,1896 |
| ZNF781 | 1,41 | 0,0464 | UNKL | -1,5 | 0,0497 | STAT1 | 2,24 | 0,163 |
| ZNF791 | 1,67 | 0,0158 | UQCC2 | -2,14 | 0,1195 | STAT5A | -1,6 | 0,0486 |
| ZNF804A | -2,31 | 0,3537 | URGCP | 1,88 | 0,0239 | STAT5B | -2,02 | 0,1632 |
| ZNF852 | -2,33 | 0,1487 | USP21 | -2,25 | 0,9398 | STEAP1B | 2,2 | 0,4278 |
| ZP3 | -3,25 | 0,0730 | UTP20 | 2,06 | 0,5350 | STIL | -2,64 | 0,2114 |
|  |  |  | VAC14 | 2,16 | 0,2249 | STK26 | 2,9 | 0,12 |
|  |  |  | VAPA | 2,12 | 0,0131 | STK32B | -2,17 | 0,5584 |
|  |  |  | VASH1 | 2,97 | 0,5050 | STK39 | 2,18 | 0,013 |
|  |  |  | VEGFC | 2,46 | 0,8837 | STMN1 | -4,26 | 0,1089 |
|  |  |  | VEPH1 | 3,74 | 0,5582 | STRIP2 | -3,24 | 0,0298 |
|  |  |  | VIT | -3,82 | 0,8606 | STX11 | -1,39 | 0,0419 |
|  |  |  | VPS33B | 1,88 | 0,0240 | STX17 | 1,53 | 0,0444 |
|  |  |  | VSNL1 | -1,3 | 0,0337 | STX3 | -2,35 | 0,4785 |
|  |  |  | VWDE | -1,36 | 0,0146 | STX6 | -2 | 0,0256 |
|  |  |  | WDR17 | -2,43 | 0,3271 | STXBP6 | 1,87 | 0,033 |
|  |  |  | WDR19 | 3,51 | 0,0017 | SUFU | -1,35 | 0,019 |
|  |  |  | WDR34 | 3,21 | 0,1304 | SULF2 | -3,25 | 0,0518 |
|  |  |  | WDR36 | 2,23 | 0,1869 | SULT1B1 | -2,12 | 0,2781 |
|  |  |  | WDR63 | -2,02 | 0,7329 | SUPV3L1 | -2,61 | 0,1046 |
|  |  |  | WDR7 | -1,35 | 0,0290 | SUSD5 | 1,74 | 0,0142 |
|  |  |  | WDR73 | 2,37 | 0,0777 | SUV39H2 | -3,24 | 0,0778 |
|  |  |  | WDR76 | 2,9 | 0,1808 | SWAP70 | -1,29 | 0,0427 |
|  |  |  | WFDC3 | -3 | 0,0367 | SYBU | -1,33 | 0,0301 |
|  |  |  | WHSC1 | 2,91 | 0,1323 | SYNGR2 | 2,04 | 0,8461 |
|  |  |  | WISP1 | 2,03 | 0,1269 | SYS1 | 2,29 | 0,0118 |
|  |  |  | WIZ | -1,45 | 0,0135 | SYT15 | -2,47 | 0,5126 |
|  |  |  | WNT2B | -2,11 | 0,0969 | SYT3 | 1,25 | 0,0274 |
|  |  |  | WNT5A | 3,04 | 0,6896 | SYTL4 | -1,39 | 0,0396 |
|  |  |  | WRN | 4,55 | 0,0497 | SZT2 | -1,81 | 0,0273 |
|  |  |  | WWC1 | -2,07 | 0,0416 | TACC3 | -18,84 | 0,1573 |
|  |  |  | WWC2 | -2,15 | 0,3868 | TADA2A | -3,33 | 0,0712 |
|  |  |  | XKR5 | 1,29 | 0,0409 | TAF7L | -1,45 | 0,0445 |
|  |  |  | XKR9 | -2,46 | 0,0451 | TAGLN | 2,34 | 0,4594 |
|  |  |  | XPNPEP3 | -3,01 | 0,0252 | TANC2 | 2,16 | 0,2074 |
|  |  |  | XRCC2 | 3,45 | 0,4802 | TANK | 1,58 | 0,0349 |
|  |  |  | XRCC4 | -2,66 | 0,0105 | TARDBP | -1,81 | 0,0265 |
|  |  |  | XRCC6BP1 | 2,15 | 0,9380 | TBC1D14 | -1,47 | 0,0406 |
|  |  |  | XYLT1 | -2,59 | 0,6483 | TBC1D15 | -2,22 | 0,0046 |
|  |  |  | YWHAE | 1,51 | 0,0476 | TBC1D3 | -1,66 | 0,0014 |
|  |  |  | YY1AP1 | 1,51 | 0,0084 | TBC1D8 | -1,83 | 0,0292 |
|  |  |  | ZADH2 | 2,11 | 0,0567 | TBC1D9B | -2,07 | 0,1354 |
|  |  |  | ZBTB14 | 3,8 | 0,0288 | TBCD | 2,21 | 0,272 |
|  |  |  | ZCCHC17 | -1,5 | 0,0377 | TBRG1 | -1,83 | 0,0275 |
|  |  |  | ZCWPW2 | -2,09 | 0,0006 | TBRG4 | -2,4 | 0,1809 |
|  |  |  | ZDHHC18 | 2,19 | 0,2170 | TBX2 | 3,49 | 0,1505 |
|  |  |  | ZDHHC2 | -2,08 | 0,9536 | TBX20 | -2,32 | 0,0199 |
|  |  |  | ZEB1 | 2,58 | 0,0396 | TCAIM | 2,67 | 0,7859 |
|  |  |  | ZFAND2A | -3,61 | 0,0096 | TCEA1 | -3,09 | 0,1871 |
|  |  |  | ZFAND4 | 3,51 | 0,0557 | TCEAL1 | 2 | 0,115 |
|  |  |  | ZFAND6 | 2 | 0,0832 | TCEAL3 | 2,18 | 0,0357 |
|  |  |  | ZGRF1 | 2,57 | 0,0326 | TCEAL8 | 1,89 | 0,0188 |
|  |  |  | ZKSCAN7 | 1 | 0,0368 | TCF15 | -1,36 | 0,0466 |
|  |  |  | ZMYND8 | 2,52 | 0,4443 | TCN1 | 1,18 | 0,0243 |
|  |  |  | ZNF100 | 2,1 | 0,1539 | TCP11 | 1,22 | 0,0351 |
|  |  |  | ZNF142 | 2,49 | 0,1071 | TCP11L1 | 2,28 | 0,1591 |
|  |  |  | ZNF143 | 2,03 | 0,2969 | TCTA | 2,45 | 0,4195 |
|  |  |  | ZNF184 | 2,35 | 0,1814 | TCTEX1D4 | 1,29 | 0,0465 |
|  |  |  | ZNF215 | 2,77 | 0,0459 | TDRD7 | -2,12 | 0,0441 |
|  |  |  | ZNF223 | 1,97 | 0,0209 | TEAD2 | -2,04 | 0,0838 |
|  |  |  | ZNF227 | -1,8 | 0,0249 | TEAD3 | 2,02 | 0,1296 |
|  |  |  | ZNF239 | -1,42 | 0,0254 | TEX30 | -3,19 | 0,6586 |
|  |  |  | ZNF25 | 2,8 | 0,2750 | TEX35 | 1,32 | 0,0183 |
|  |  |  | ZNF280B | 2,32 | 0,0515 | TFAM | -2,11 | 0,2103 |
|  |  |  | ZNF287 | -2,69 | 0,0147 | TFDP1 | -2,25 | 0,2574 |
|  |  |  | ZNF304 | 2,86 | 0,6203 | TFPI | 3,64 | 0,5153 |
|  |  |  | ZNF331 | 3,01 | 0,3486 | TFPI2 | 3,01 | 0,261 |
|  |  |  | ZNF383 | -3,51 | 0,0006 | TGFB2 | 3,57 | 0,1697 |
|  |  |  | ZNF415 | -2,34 | 0,2771 | TGFBR1 | 2,29 | 0,907 |
|  |  |  | ZNF433 | -1,87 | 0,0292 | TGFBR3 | 2,29 | 0,6431 |
|  |  |  | ZNF454 | -3,22 | 0,0210 | TGIF1 | -2,48 | 0,2406 |
|  |  |  | ZNF467 | -1,29 | 0,0458 | TGIF2 | -4,03 | 0,327 |
|  |  |  | ZNF512 | 2,13 | 0,0245 | THNSL2 | 4,72 | 0,0445 |
|  |  |  | ZNF514 | -1,72 | 0,0136 | THOC3 | -2,02 | 0,623 |
|  |  |  | ZNF530 | -1,32 | 0,0407 | THOC6 | -2,23 | 0,0481 |
|  |  |  | ZNF532 | 2,64 | 0,4236 | THRAP3 | -1,75 | 0,0303 |
|  |  |  | ZNF559-ZNF177 | -2,57 | 0,3021 | THSD1 | -2,27 | 0,148 |
|  |  |  | ZNF564 | -1,24 | 0,0401 | TICRR | -2,09 | 0,0062 |
|  |  |  | ZNF584 | -2,17 | 0,0440 | TIGAR | 1,87 | 0,0198 |
|  |  |  | ZNF605 | -2,06 | 0,5209 | TIMP1 | 2,59 | 0,535 |
|  |  |  | ZNF608 | 2,03 | 0,0114 | TINAG | 1,42 | 0,0121 |
|  |  |  | ZNF610 | -2,21 | 0,0285 | TINAGL1 | 4,01 | 0,9332 |
|  |  |  | ZNF652 | -2,22 | 0,0052 | TK1 | -2,71 | 0,1051 |
|  |  |  | ZNF654 | -2,21 | 0,0648 | TK2 | 2 | 0,1532 |
|  |  |  | ZNF667 | -2,41 | 0,0176 | TLCD1 | 2,12 | 0,1314 |
|  |  |  | ZNF684 | 2,18 | 0,1793 | TLK1 | 1,56 | 0,0076 |
|  |  |  | ZNF708 | 2,22 | 0,0576 | TM9SF4 | 1,34 | 0,0462 |
|  |  |  | ZNF709 | -1,58 | 0,0019 | TMED7-TICAM2 | 1,32 | 0,0316 |
|  |  |  | ZNF713 | 2,29 | 0,4781 | TMEFF1 | -2,22 | 0,1658 |
|  |  |  | ZNF717 | -2,13 | 0,9974 | TMEM106A | 2,82 | 0,0558 |
|  |  |  | ZNF718 | 3,1 | 0,4691 | TMEM106B | 2,02 | 0,0682 |
|  |  |  | ZNF729 | -1,52 | 0,0298 | TMEM107 | 2,01 | 0,1154 |
|  |  |  | ZNF772 | 2,24 | 0,0836 | TMEM109 | -2,6 | 0,0909 |
|  |  |  | ZNF773 | -2,26 | 0,2163 | TMEM128 | 2,54 | 0,0465 |
|  |  |  | ZNF776 | 1,56 | 0,0301 | TMEM150B | -1,22 | 0,0363 |
|  |  |  | ZNF780A | 1 | 0,0306 | TMEM154 | -6,61 | 0,0347 |
|  |  |  | ZNF782 | 2,13 | 0,0993 | TMEM165 | 1,69 | 0,0492 |
|  |  |  | ZNF846 | -2,21 | 0,0149 | TMEM167A | 1,52 | 0,0429 |
|  |  |  | ZNF852 | -3,63 | 0,1394 | TMEM173 | -2,46 | 0,0399 |
|  |  |  | ZNRD1 | 2,01 | 0,0211 | TMEM181 | 1,72 | 0,0443 |
|  |  |  | ZSCAN29 | -2,08 | 0,4774 | TMEM183B | -1,34 | 0,0274 |
|  |  |  | ZWINT | 3,54 | 0,2234 | TMEM184A | 1,33 | 0,0244 |
|  |  |  |  |  |  | TMEM19 | -2,57 | 0,2504 |
|  |  |  |  |  |  | TMEM210 | -1,35 | 0,0145 |
|  |  |  |  |  |  | TMEM217 | 1,99 | 0,0429 |
|  |  |  |  |  |  | TMEM240 | 1,35 | 0,0216 |
|  |  |  |  |  |  | TMEM255B | -4,47 | 0,0341 |
|  |  |  |  |  |  | TMEM263 | 2,28 | 0,0076 |
|  |  |  |  |  |  | TMEM265 | -1,27 | 0,0284 |
|  |  |  |  |  |  | TMEM31 | -1,34 | 0,0198 |
|  |  |  |  |  |  | TMEM54 | -2,16 | 0,0159 |
|  |  |  |  |  |  | TMEM59 | 1,85 | 0,003 |
|  |  |  |  |  |  | TMEM64 | 1,36 | 0,021 |
|  |  |  |  |  |  | TMF1 | 1,62 | 0,0312 |
|  |  |  |  |  |  | TMOD3 | -1,68 | 0,0076 |
|  |  |  |  |  |  | TMPO | -2,86 | 0,0064 |
|  |  |  |  |  |  | TMTC1 | 2,79 | 0,2116 |
|  |  |  |  |  |  | TMTC2 | 2,01 | 0,6865 |
|  |  |  |  |  |  | TMX3 | 2,39 | 0,1436 |
|  |  |  |  |  |  | TNC | 3,29 | 0,2093 |
|  |  |  |  |  |  | TNFAIP3 | 3,58 | 0,0172 |
|  |  |  |  |  |  | TNFAIP8 | 2,1 | 0,1054 |
|  |  |  |  |  |  | TNFRSF11B | 3,35 | 0,2088 |
|  |  |  |  |  |  | TNFRSF21 | -2,97 | 0,1252 |
|  |  |  |  |  |  | TNFRSF9 | -2,44 | 0,3219 |
|  |  |  |  |  |  | TNNC2 | 1,95 | 0,0277 |
|  |  |  |  |  |  | TOM1 | 1,93 | 0,0337 |
|  |  |  |  |  |  | TOMM7 | 1,69 | 0,0135 |
|  |  |  |  |  |  | TOP2A | -3,31 | 0,2742 |
|  |  |  |  |  |  | TOP3B | -2,25 | 0,1493 |
|  |  |  |  |  |  | TOR1AIP1 | 2,33 | 0,3076 |
|  |  |  |  |  |  | TOX | 3,29 | 0,6813 |
|  |  |  |  |  |  | TP53I3 | 2,32 | 0,691 |
|  |  |  |  |  |  | TPCN1 | 4,35 | 0,4506 |
|  |  |  |  |  |  | TPD52 | 2,42 | 0,5702 |
|  |  |  |  |  |  | TPM1 | 2,72 | 0,1641 |
|  |  |  |  |  |  | TPM2 | 2,87 | 0,0606 |
|  |  |  |  |  |  | TRABD2A | 1,24 | 0,0225 |
|  |  |  |  |  |  | TRAF3IP1 | 1,46 | 0,0324 |
|  |  |  |  |  |  | TRAIP | -1,46 | 0,0417 |
|  |  |  |  |  |  | TRAV9-1 | -1,25 | 0,0314 |
|  |  |  |  |  |  | TRBJ1-3 | 2,01 | 0,165 |
|  |  |  |  |  |  | TRBV6-6 | -1,76 | 0,0077 |
|  |  |  |  |  |  | TRIB2 | 2,51 | 0,6601 |
|  |  |  |  |  |  | TRIM25 | -2,16 | 0,0107 |
|  |  |  |  |  |  | TRIM28 | -1,75 | 0,0361 |
|  |  |  |  |  |  | TRMT61B | -2,72 | 0,9195 |
|  |  |  |  |  |  | TRMU | -2,51 | 0,0393 |
|  |  |  |  |  |  | TRPC1 | 2,37 | 0,0797 |
|  |  |  |  |  |  | TRPC4 | 2,74 | 0,0665 |
|  |  |  |  |  |  | TRPV2 | 108,64 | 0,0007 |
|  |  |  |  |  |  | TRRAP | -1,45 | 0,0371 |
|  |  |  |  |  |  | TSEN2 | -2,38 | 0,4725 |
|  |  |  |  |  |  | TSGA10 | 1,18 | 0,0155 |
|  |  |  |  |  |  | TSHZ3 | -2 | 0,8265 |
|  |  |  |  |  |  | TSPAN13 | 2,66 | 0,0309 |
|  |  |  |  |  |  | TSPAN4 | -2,75 | 0,1484 |
|  |  |  |  |  |  | TSPAN5 | 2,43 | 0,1916 |
|  |  |  |  |  |  | TSSC4 | -1,39 | 0,0479 |
|  |  |  |  |  |  | TSTD3 | 1,49 | 0,0288 |
|  |  |  |  |  |  | TTC3 | 2,05 | 0,0209 |
|  |  |  |  |  |  | TTC33 | 1,56 | 0,0134 |
|  |  |  |  |  |  | TTC39C | -2,09 | 0,1987 |
|  |  |  |  |  |  | TTK | -5,94 | 0,3155 |
|  |  |  |  |  |  | TTL | -1,58 | 0,0356 |
|  |  |  |  |  |  | TTLL12 | -2,17 | 0,2131 |
|  |  |  |  |  |  | TTPA | 1,17 | 0,0354 |
|  |  |  |  |  |  | TUBB4B | -3,03 | 0,0838 |
|  |  |  |  |  |  | TUBB8 | -2,17 | 0,3332 |
|  |  |  |  |  |  | TUBG1 | -3,08 | 0,1533 |
|  |  |  |  |  |  | TUSC3 | 3,48 | 0,0819 |
|  |  |  |  |  |  | TVP23B | 1,75 | 0,0008 |
|  |  |  |  |  |  | TVP23C | 1,45 | 0,0426 |
|  |  |  |  |  |  | TXNDC5 | -2,23 | 0,0708 |
|  |  |  |  |  |  | TXNIP | -3,1 | 0,1651 |
|  |  |  |  |  |  | TYK2 | -2,04 | 0,058 |
|  |  |  |  |  |  | TYMS | -5,21 | 0,2943 |
|  |  |  |  |  |  | U1 | 2,02 | 0,0634 |
|  |  |  |  |  |  | U2AF1 | -1,71 | 0,0213 |
|  |  |  |  |  |  | U4 | -2,69 | 0,0023 |
|  |  |  |  |  |  | UACA | 5,38 | 0,0227 |
|  |  |  |  |  |  | UBA2 | -1,9 | 0,0445 |
|  |  |  |  |  |  | UBE2C | -2,66 | 0,4656 |
|  |  |  |  |  |  | UBE2T | -2,4 | 0,4181 |
|  |  |  |  |  |  | UBE3B | 2,03 | 0,0568 |
|  |  |  |  |  |  | UBIAD1 | -2,08 | 0,2092 |
|  |  |  |  |  |  | UBLCP1 | 1,7 | 0,0085 |
|  |  |  |  |  |  | UBQLNL | 1,2 | 0,0291 |
|  |  |  |  |  |  | UBXN2B | 1,6 | 0,001 |
|  |  |  |  |  |  | UCHL1 | 2,63 | 0,1104 |
|  |  |  |  |  |  | UFC1 | 1,51 | 0,0208 |
|  |  |  |  |  |  | UFM1 | 1,34 | 0,0477 |
|  |  |  |  |  |  | UGGT2 | 2,67 | 0,3813 |
|  |  |  |  |  |  | UHRF1 | -2,96 | 0,1824 |
|  |  |  |  |  |  | UHRF2 | 2,64 | 0,2393 |
|  |  |  |  |  |  | UMPS | -2,15 | 0,1082 |
|  |  |  |  |  |  | UNC50 | 1,51 | 0,0087 |
|  |  |  |  |  |  | UNC5B | -2,49 | 0,8135 |
|  |  |  |  |  |  | UPK3BL | 2,36 | 0,1106 |
|  |  |  |  |  |  | UQCR11 | 1,17 | 0,0262 |
|  |  |  |  |  |  | UQCRB | 1,28 | 0,0421 |
|  |  |  |  |  |  | USP17L7 | 1,16 | 0,0462 |
|  |  |  |  |  |  | USP30 | -1,9 | 0,0313 |
|  |  |  |  |  |  | USP53 | -2,62 | 0,7295 |
|  |  |  |  |  |  | USP6NL | -2,67 | 0,0024 |
|  |  |  |  |  |  | UTP3 | 1,31 | 0,0384 |
|  |  |  |  |  |  | UTRN | -1,48 | 0,0261 |
|  |  |  |  |  |  | VAMP1 | 4,83 | 0,0716 |
|  |  |  |  |  |  | VAMP2 | 1,64 | 0,0361 |
|  |  |  |  |  |  | VANGL1 | -1,91 | 0,0061 |
|  |  |  |  |  |  | VAPA | -1,64 | 0,0087 |
|  |  |  |  |  |  | VASH1 | -2,49 | 0,0368 |
|  |  |  |  |  |  | VAT1 | -2,45 | 0,18 |
|  |  |  |  |  |  | VDR | 3,16 | 0,1987 |
|  |  |  |  |  |  | VEPH1 | -2,43 | 0,7185 |
|  |  |  |  |  |  | VGLL3 | 5,59 | 0,3227 |
|  |  |  |  |  |  | VPS41 | 2,28 | 0,172 |
|  |  |  |  |  |  | VPS45 | -2,19 | 0,2158 |
|  |  |  |  |  |  | VRK1 | -2,08 | 0,2967 |
|  |  |  |  |  |  | VSNL1 | 2,23 | 0,1258 |
|  |  |  |  |  |  | VWA9 | -2,08 | 0,0118 |
|  |  |  |  |  |  | WARS | -4,32 | 0,0402 |
|  |  |  |  |  |  | WASF1 | -2,05 | 0,0016 |
|  |  |  |  |  |  | WASH1 | -1,79 | 0,0203 |
|  |  |  |  |  |  | WBP5 | 1,78 | 0,0297 |
|  |  |  |  |  |  | WDHD1 | -2,02 | 0,1707 |
|  |  |  |  |  |  | WDR17 | 2,07 | 0,5354 |
|  |  |  |  |  |  | WDR27 | -1,71 | 0,0195 |
|  |  |  |  |  |  | WDR34 | -4,43 | 0,0442 |
|  |  |  |  |  |  | WDR35 | -1,58 | 0,0405 |
|  |  |  |  |  |  | WDR36 | -2,56 | 0,2168 |
|  |  |  |  |  |  | WDR54 | -2,13 | 0,1578 |
|  |  |  |  |  |  | WDR63 | 2,24 | 0,0755 |
|  |  |  |  |  |  | WDR76 | -3,41 | 0,1044 |
|  |  |  |  |  |  | WDR82 | -2,55 | 0,0911 |
|  |  |  |  |  |  | WDR83 | -1,57 | 0,0352 |
|  |  |  |  |  |  | WDYHV1 | 1,52 | 0,0479 |
|  |  |  |  |  |  | WFDC3 | 2,1 | 0,0591 |
|  |  |  |  |  |  | WFDC8 | -1,2 | 0,034 |
|  |  |  |  |  |  | WHSC1 | -3,22 | 0,1192 |
|  |  |  |  |  |  | WIPI1 | 2,45 | 0,2138 |
|  |  |  |  |  |  | WNT5A | 2,83 | 0,4557 |
|  |  |  |  |  |  | WNT9B | -1,15 | 0,0337 |
|  |  |  |  |  |  | WRAP53 | -2,93 | 0,0188 |
|  |  |  |  |  |  | WRN | -2,97 | 0,2548 |
|  |  |  |  |  |  | WSB1 | -2,61 | 0,0836 |
|  |  |  |  |  |  | WWC1 | 2,12 | 0,0267 |
|  |  |  |  |  |  | WWC3 | -2,01 | 0,4641 |
|  |  |  |  |  |  | XPOT | -2,18 | 0,1012 |
|  |  |  |  |  |  | XRCC1 | -2,77 | 0,0474 |
|  |  |  |  |  |  | XRCC2 | -2,61 | 0,1491 |
|  |  |  |  |  |  | XRRA1 | 1,47 | 0,0408 |
|  |  |  |  |  |  | YAF2 | 2,09 | 0,0353 |
|  |  |  |  |  |  | YIPF4 | 1,39 | 0,037 |
|  |  |  |  |  |  | YPEL5 | 2,33 | 0,0023 |
|  |  |  |  |  |  | YY1AP1 | -1,25 | 0,0451 |
|  |  |  |  |  |  | ZBTB12 | -1,34 | 0,0218 |
|  |  |  |  |  |  | ZBTB14 | -2,56 | 0,2085 |
|  |  |  |  |  |  | ZBTB39 | -1,78 | 0,0439 |
|  |  |  |  |  |  | ZBTB42 | 1,32 | 0,0215 |
|  |  |  |  |  |  | ZBTB7C | 1,25 | 0,0379 |
|  |  |  |  |  |  | ZBTB8B | -1,22 | 0,039 |
|  |  |  |  |  |  | ZC3H12A | 2,35 | 0,3734 |
|  |  |  |  |  |  | ZCCHC8 | -1,76 | 0,022 |
|  |  |  |  |  |  | ZCCHC9 | 2,02 | 0,0228 |
|  |  |  |  |  |  | ZCWPW2 | 2,07 | 0,0386 |
|  |  |  |  |  |  | ZDHHC11B | 1,38 | 0,0033 |
|  |  |  |  |  |  | ZDHHC6 | -1,64 | 0,0113 |
|  |  |  |  |  |  | ZEB1 | -2,09 | 0,0084 |
|  |  |  |  |  |  | ZFAND2A | 3,97 | 0,0151 |
|  |  |  |  |  |  | ZFHX4 | 2,51 | 0,227 |
|  |  |  |  |  |  | ZFP30 | 1,48 | 0,0282 |
|  |  |  |  |  |  | ZFP82 | 2,21 | 0,0000506 |
|  |  |  |  |  |  | ZGRF1 | -2,57 | 0,3947 |
|  |  |  |  |  |  | ZHX2 | 2,18 | 0,6021 |
|  |  |  |  |  |  | ZMIZ1 | -1,51 | 0,0322 |
|  |  |  |  |  |  | ZMYM1 | -1,74 | 0,0246 |
|  |  |  |  |  |  | ZMYND8 | -2,52 | 0,4891 |
|  |  |  |  |  |  | ZNF100 | -2,2 | 0,1816 |
|  |  |  |  |  |  | ZNF142 | -2,16 | 0,0775 |
|  |  |  |  |  |  | ZNF182 | -1,42 | 0,0028 |
|  |  |  |  |  |  | ZNF185 | 3,95 | 0,1309 |
|  |  |  |  |  |  | ZNF207 | -1,16 | 0,0378 |
|  |  |  |  |  |  | ZNF215 | -4,3 | 0,0000103 |
|  |  |  |  |  |  | ZNF22 | -1,73 | 0,0377 |
|  |  |  |  |  |  | ZNF239 | 1,98 | 0,0035 |
|  |  |  |  |  |  | ZNF268 | -1,78 | 0,0231 |
|  |  |  |  |  |  | ZNF273 | -1,5 | 0,0082 |
|  |  |  |  |  |  | ZNF280B | -1,68 | 0,0241 |
|  |  |  |  |  |  | ZNF282 | -2,18 | 0,0324 |
|  |  |  |  |  |  | ZNF284 | -3,6 | 0,1579 |
|  |  |  |  |  |  | ZNF319 | -1,26 | 0,0197 |
|  |  |  |  |  |  | ZNF331 | -3,79 | 0,0919 |
|  |  |  |  |  |  | ZNF385D | -6,59 | 0,0638 |
|  |  |  |  |  |  | ZNF415 | 2,63 | 0,1721 |
|  |  |  |  |  |  | ZNF416 | 1,41 | 0,0385 |
|  |  |  |  |  |  | ZNF423 | 2,06 | 0,2449 |
|  |  |  |  |  |  | ZNF430 | -1,69 | 0,0128 |
|  |  |  |  |  |  | ZNF454 | 3,4 | 0,1176 |
|  |  |  |  |  |  | ZNF491 | 1,36 | 0,0068 |
|  |  |  |  |  |  | ZNF492 | -2,17 | 0,001 |
|  |  |  |  |  |  | ZNF506 | 1,41 | 0,032 |
|  |  |  |  |  |  | ZNF512 | -2,52 | 0,059 |
|  |  |  |  |  |  | ZNF529 | -1,56 | 0,0018 |
|  |  |  |  |  |  | ZNF530 | 1,26 | 0,0328 |
|  |  |  |  |  |  | ZNF559-ZNF177 | 2,9 | 0,1001 |
|  |  |  |  |  |  | ZNF561 | 1,48 | 0,0315 |
|  |  |  |  |  |  | ZNF565 | 1,55 | 0,0289 |
|  |  |  |  |  |  | ZNF566 | 1,6 | 0,0439 |
|  |  |  |  |  |  | ZNF567 | 3,26 | 0,0022 |
|  |  |  |  |  |  | ZNF583 | 1,18 | 0,0259 |
|  |  |  |  |  |  | ZNF595 | 1,5 | 0,0323 |
|  |  |  |  |  |  | ZNF606 | 1,24 | 0,0335 |
|  |  |  |  |  |  | ZNF653 | -1,26 | 0,0308 |
|  |  |  |  |  |  | ZNF672 | -1,58 | 0,0363 |
|  |  |  |  |  |  | ZNF717 | 2,89 | 0,1743 |
|  |  |  |  |  |  | ZNF782 | -1,73 | 0,0399 |
|  |  |  |  |  |  | ZNF806 | 1,33 | 0,0351 |
|  |  |  |  |  |  | ZNF821 | 1,36 | 0,0088 |
|  |  |  |  |  |  | ZNF841 | 1,63 | 0,0462 |
|  |  |  |  |  |  | ZNF846 | 3,43 | 0,0195 |
|  |  |  |  |  |  | ZNF852 | 2,31 | 0,1388 |
|  |  |  |  |  |  | ZNF883 | 3,05 | 0,0002 |
|  |  |  |  |  |  | ZNFX1 | 1,62 | 0,0135 |
|  |  |  |  |  |  | ZNRD1 | -1,66 | 0,0183 |
|  |  |  |  |  |  | ZP3 | -3,07 | 0,0412 |
|  |  |  |  |  |  | ZPLD1 | 1,41 | 0,0478 |
|  |  |  |  |  |  | ZWINT | -3,59 | 0,1845 |
